# Is Postural Control Affected in People with Patellofemoral Pain and Should it be Part of Rehabilitation? A Systematic Review with Meta-analysis

**DOI:** 10.1186/s40798-022-00538-4

**Published:** 2022-12-12

**Authors:** Guilherme S. Nunes, Diênifer Zilmer Rodrigues, Luiza Hörbe, Izabela Prates, Bruna M. Tessarin, Fábio V. Serrão, Marcos de Noronha

**Affiliations:** 1 Department of Physiotherapy and Rehabilitation, Federal University of Santa Maria, Av. Roraima, 1000, Santa Maria, RS CEP 97105-900 Brazil; 2 Department of Physiotherapy, São Carlos Federal University, São Carlos, Brazil; 3 Rural Department of Allied Health, La Trobe University, Bendigo, VIC Australia

**Keywords:** Anterior knee pain, Knee, Balance, Exercise, Treatment

## Abstract

**Background:**

Growing evidence supports that exercise therapy is effective for patellofemoral pain (PFP) rehabilitation. Nevertheless, the improvements have been reported not to be sustained in the long term, suggesting that the current protocols may not comprehend all required functional factors to provide a consistent recovery. A potential neglected factor in treatment protocols for PFP is postural control. However, it is unclear whether this population presents balance impairments or the influence of postural control on pain and function during rehabilitation programmes.

**Objective:**

To investigate whether (Q1) balance is impaired in people with PFP compared to controls, (Q2) conservative interventions are effective to improve balance in people with PFP, and (Q3) balance exercises are effective to improve pain and function in people with PFP.

**Data sources:**

Medline, Embase, CINAHL, SPORTDiscus, Web of Science and Cochrane Library, supplemented by hand searching of reference lists, citations and relevant systematic reviews in the field.

**Methods:**

A systematic review with meta-analysis was conducted according to the Cochrane recommendations and reported according to the PRISMA statement recommendations. We included cross-sectional studies comparing balance between people with and without PFP; and randomised controlled trials verifying the effect of conservative intervention on balance and the effect of balance intervention on pain and function in people with PFP. The risk of bias was assessed using the Epidemiological Appraisal Instrument for cross-sectional studies and the Physiotherapy Evidence Database scale for randomised controlled trials.

**Results:**

From 15,436 records, 57 studies (Q1 = 28, Q2 = 23, Q3 = 14) met the eligibility criteria. Meta-analyses indicated that people with PFP have worse anteroposterior (very low grade evidence, standardised mean difference [SMD] = 1.03, 95% CI 0.40–1.66) and mediolateral (moderate grade evidence, SMD = 0.87, 95% CI 0.31–1.42) balance compared to controls. Moderate grade evidence indicated that overall balance is not affected in people with PFP (SMD = 0.38, 95% CI − 0.05–0.82). Low to very low grade evidence indicates that interventions are ineffective for mediolateral (SMD = 0.01, 95% CI − 0.51–0.53) and overall (SMD = 0.49, 95% CI − 0.14–1.11) balance improvements, and low grade evidence indicates that interventions are effective to improve anteroposterior balance (SMD = 0.64, 95% CI 0.04–1.23). Moderate to low grade evidence indicated that balance interventions are effective to reduce pain (SMD = 0.82, 95% CI 0.26–1.38) and improve function (SMD = 0.44, 95% CI 0.09–0.80) when measured using questionnaires; and very low grade evidence indicated no efficacy for function measured via functional tests (SMD = 0.73, 95% CI − 0.16–1.61).

**Conclusion:**

People with PFP likely present balance deficits compared to asymptomatic people. There was insufficient evidence to support the efficacy of interventions to improve or modify balance in people with PFP. Also, there was insufficient evidence to support the efficacy of balance exercises to improve pain and function in people with PFP.

*Trial Registration* The present systematic review was registered in PROSPERO (CRD42018091717).

**Supplementary Information:**

The online version contains supplementary material available at 10.1186/s40798-022-00538-4.

**Key Points**

- Balance is likely impaired in people with patellofemoral pain compared to asymptomatic people.
- It is uncertain whether conservative interventions are effective in improving balance in people with patellofemoral pain.
- The efficacy of exercise programmes that included balance exercise to address pain or function in people with patellofemoral pain is arguable.

## Introduction

Patellofemoral pain (PFP) is a frequent disorder in the general population, with an annual prevalence of up to 23% [1]. In the USA, more than two million people were diagnosed with PFP between 2007 and 2011 [2]. This condition has no spontaneous recovery [3, 4] and, therefore, requires treatment [5]. Growing evidence supports that exercise therapy protocols are effective rehabilitation for people with PFP [5–8]. However, pain and function improvements have been reported not to be sustained in the long term [6, 9, 10]. This indicates that the current protocols may not comprehend all required functional factors to provide a full and consistent recovery for that population.

A potential neglected factor in treatment protocols for PFP is postural control [5, 11–14]. Postural control involves a complex integration of visual, vestibular and somatosensory systems based on reflex actions occurring to maintain balance [15–17]. Considering people with PFP have impaired H-reflex [18, 19], it is reasonable to expect that people with PFP will have alterations in other neuromuscular reflexes which may impact balance. Additionally, the presence of pain in people with PFP may also lead to impairments in postural control [20, 21]. The nociceptive information potentially impairs information from mechanoreceptors [20, 21], and consequently, may delay reflexes and actions required to maintain balance [20–22]. Some studies have evaluated balance in people with PFP; however, the respective results are conflicting [23–27]. For example, Saad et al. [27] reported that females with PFP have a greater centre of pressure (CoP) displacement during a stair ascent task compared to asymptomatic females. Contrastingly, Silva et al. [26] reported that females with PFP have decreased CoP displacement during the same task compared to asymptomatic females. Therefore, there is uncertainty regarding balance impairments in the population with PFP. Interestingly, some research investigating the efficacy of interventions for PFP has used balance measures as outcomes, e.g. CoP behaviour during different tasks [28, 29] and Star Excursion Balance Test (SEBT) [30], although it is unclear if people with PFP actually present balance deficits and if balance is a modifiable outcome in this population. Also, some research studies included balance exercises in their protocols [28, 31, 32] and activities challenging the control of centre of gravity inside the base of support [33, 34], and their importance for PFP rehabilitation remains unknown.

Excessive dynamic knee valgus, including excessive movements at the hip [35, 36] and at the ankle [37], during activities is thought to be an important biomechanical factor for PFP due to a potential increase in the lateral force acting on the patella [35, 36, 38]. Although hip and ankle kinematic alterations are not risk factors for PFP development [39], people with PFP have presented excessive hip and ankle movements during activities [11, 40, 41]. As a consequence, interventions targeting hip and ankle joints have been suggested and reported to be effective for PFP rehabilitation, such as hip muscle strengthening [6] and foot orthoses [42]. Interestingly, adjustment movements at the ankle and hip, also called “ankle strategy” and “hip strategy”, are adopted to keep the centre of gravity close to the support base in order to maintain balance in asymptomatic people [43, 44]. Perhaps, excessive hip and ankle movements observed in people with PFP [11] may be compensations for potential impairments in postural control, and therefore, interventions targeting balance could also be beneficial for people with PFP. However, little is known about the addition of balance exercises in protocols for PFP treatment.

To understand the importance of postural control for the management of PFP, this systematic review aimed to answer three questions: (Q1) Is balance impaired in people with PFP compared to asymptomatic people? (Q2) Are conservative interventions effective to improve potential balance impairments in people with PFP? (Q3) Are balance exercises effective to improve pain and function in people with PFP?

## Methods

### Design

The review was conducted according to the Cochrane recommendations [45], reported according to PRISMA statement recommendations [46] and a priori registered at PROSPERO (CRD42018091717).

### Deviation from Protocol

We assessed the evidence quality using GRADE (Grading of Recommendations Assessment, Development and Evaluation), in order to perform a broader assessment of evidence quality [47, 48], instead of a modified version of the van Tulder criteria. [49]. Searches in the Cochrane library were also included.

### Eligibility Criteria

- *Q1*: studies were included if (i) investigation was conducted with people with PFP and asymptomatic people; (ii) evaluated balance impairments in people with PFP compared to asymptomatic people using any instrument or tool; and (iii) used a cross-sectional design or other design that permitted cross-sectional data for PFP and control groups to be extracted. We considered as balance evaluations those assessing postural stability during standing (static balance) or performing activities (dynamic balance) by using spatial–temporal measures of sway, i.e. assessments related to the centre of gravity behaviour, including displacement, velocity, area, etc., via force platform or computerised posturography; or using clinical tests, e.g. SEBT [50].
- *Q2*: studies were included if (i) investigation was conducted with people with PFP; (ii) investigated the effect of any conservative intervention for PFP; (iii) compared the experimental intervention to any alternative, control or no intervention; (iv) outcomes included balance assessed using any instrument; and (v) used a randomised controlled trial design, including crossover design. Conservative interventions were defined as any non-pharmacological and/or non-surgical interventions, including (but not limited to) exercise therapy, taping or braces [51].
- *Q3*: studies were included if (i) investigation was conducted with people with PFP; (ii) investigated the effect of balance exercises or programmes which include balance exercises targeting people with PFP; (iii) compared the experimental intervention to any intervention without balance exercises; (iv) evaluated intervention effects on pain and/or physical function using patient-reported outcome measures, e.g. visual analogue scale or questionnaires, or applying clinical tests, e.g. hop tests; and (v) used a randomised controlled trial design. Balance exercises were defined as activities which induce difficulties in controlling an adequate alignment between the centre of gravity and the base of support with the aim of improving postural control [22, 33, 34, 52]. These activities include exercises that reduce the base of support, e.g. single-legged stance; or challenge the control of the centre of gravity, e.g. exercises performed on unstable surfaces or with participants closing their eyes [22, 33, 34, 52]. We included studies that clearly described the presence of balance exercises or when it was possible to identify exercises that were specifically prescribed to improve postural control. Activities in which balance is a potential component, such as single-legged squat or landing tasks, were considered balance exercise if the study clearly stated that the exercise targeted postural control. These tasks may target different aspects of functionality, such as movement control, muscle capacity or impact absorption, and participants could be allowed to make use of varied external support elements to remain balanced, and therefore, the exercise would not target balance improvements.

We considered as PFP those participants who were clinically diagnosed with at least the presence of retropatellar or peripatellar pain, not related to traumatic events, which was aggravated during activities that overload the patellofemoral joint [13]. Protocols, reviews, letters, academic theses, congress abstracts, and case series studies were excluded. Only English-language publications were considered. No restriction on publication period was adopted.

### Search Strategy

**Fig. 1:**
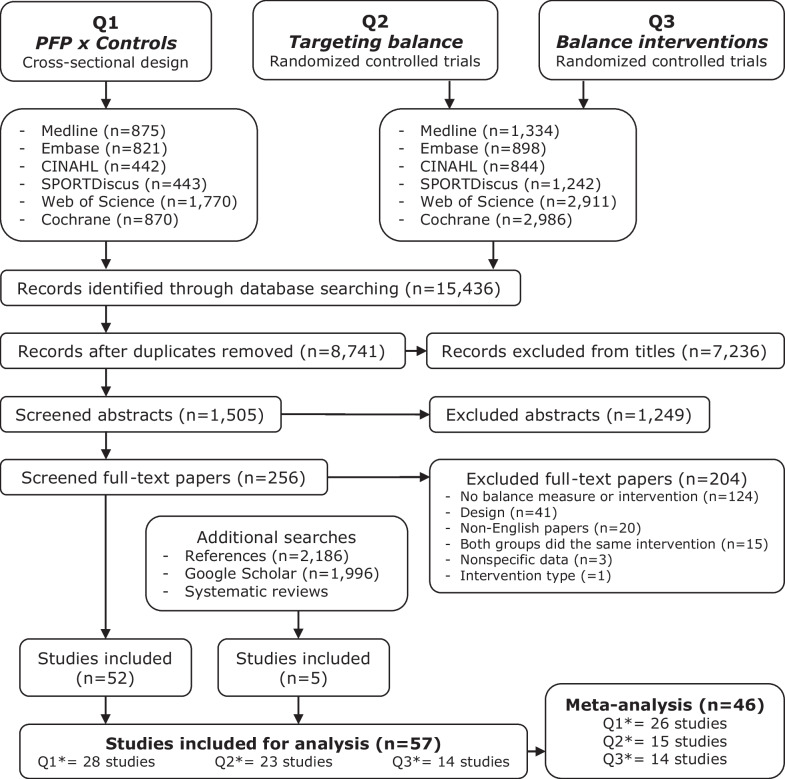
Flow diagram (*some studies fulfilled the criterion for more than one question)

Electronic searches were conducted in six databases: Medline via OVID, Embase via Elsevier, CINAHL and SPORTDiscus via EBSCO, Web of Science, and Cochrane Library from inception to August 2022. Terms related to “patellofemoral pain” and “balance” (indexed and free-text terms) were used to prepare the search strategy (Additional file 1). Reference lists of included articles, citation lists of included articles using Google Scholar, and the included studies of relevant systematic reviews [5, 7, 8, 53] were screened for eligibility (Fig. 1).

### Study Selection

First, duplicates were identified and excluded using the reference software Endnote X9 (Clarivate Analytics, Philadelphia, USA). Then, the records were exported to the software Rayyan QCRI (Qatar Computing Research Institute, Doha, Qatar) [54] which was used for the screening process. The selection was performed by two independent reviewers by titles, then by abstracts and lastly by full text (Fig. 1). The reviewers presented substantial agreement regarding the study eligibility (kappa = 0.79, based on a pilot screening with 543 records) [45, 55]. At all steps, disagreements between reviewers were resolved by consulting a third reviewer.

### Data Extraction

Data extraction was performed by two independent reviewers, and disagreements were resolved by consensus. Data extracted were: study characteristics (design, study duration, aims, sample size, funding source and settings), participant characteristics based on the REPORT-PFP Checklist (sex, age, symptoms duration, pain severity, weight, height, body mass index and recruitment settings) [56], outcomes (balance measures for Q1 and Q2; pain and function measures for Q3), analysis (data on central tendency and dispersion), and intervention characteristics (description for Q2 and Q3). Where data were missing, incompletely or unclearly reported, we contacted authors for clarification or to request the missing data. The data from two studies [57, 58] were extracted from graphs using the web-based programme WebPlotDigitizer [59, 60], as the respective authors did not respond to our request. Two studies presented the data on central tendency as median [61, 62] and one study presented the dispersion as quartiles [62]. For these cases, mean and standard deviation were estimated using the Box-Cox method [63].

### Risk of Bias Assessment

The risk of bias (RoB) was assessed by two independent reviewers, and disagreements were resolved by a third reviewer. For cross-sectional studies (Q1), 24 relevant questions from the Epidemiological Appraisal Instrument (EAI) were used [64]. The questions were selected according to our aims and relevancy for cross-sectional design [65, 66]. Each question was scored as “yes” (2 points), “partial” (1 point), “no” (0 point), or “unable to determine” (0 point). Each study received a final score calculated by dividing its total score (from 0 to 48) by 24 (total number of questions), and final scores higher than one point were considered as studies with low RoB [65, 66]. For randomised controlled trials (Q2 and Q3), specific criteria from the Physiotherapy Evidence Database scale (PEDro) were used [67]: adequacy of randomisation, allocation concealment, between-group baseline comparability, blinding of assessors, adequate follow-up, and intention-to-treat analysis. Each question was scored as “yes” (1 point) or “no” (0 point). The sum of all criteria was used in the analysis, and studies were classified as having a low RoB (≥ 5 points), moderate RoB (3–4 points) or high RoB (≤ 2 points) [67].

### Statistical Analysis

Meta-analyses for all questions were carried out using sample size, mean and standard deviation for the outcomes analysed. Only continuous data were used. Balance measures indicating postural stability indices were pooled accordingly to the direction/axis of body movement, i.e. anteroposterior, mediolateral, posteromedial, posterolateral and overall postural stability, which were measures without a defined direction, e.g. CoP area, or a composition of all measured directions, e.g. SEBT index [68–70]. Additionally, balance measures were divided into postural stability indices related to displacement, e.g. CoP area, CoP displacement or SEBT; and related to velocity, i.e. CoP velocity [68–70]. For Q2 and Q3, end-of treatment results were applied in meta-analysis. For meta-analyses related to Q3, results from studies that included more than one group with balance exercises were combined [71].

Analyses were conducted with R statistical software 4.1.1 (package meta), using random-effects modes estimated via the DerSimonian and Laird method [45]. Results are presented as standardised mean differences (SMD) with a 95% confidence interval (CI) (Hedges’ g) due to differences in instruments and/or units of measure. Pooled SMDs were categorised as trivial (< 0.2), small (≥ 0.2 to < 0.5), moderate (≥ 0.5 to < 0.8), large (≥ 0.8 to < 1.20) and very large (≥ 1.2) [72].

The statistical heterogeneity of the meta-analyses was assessed using the Higgins’ *I*^2^ measure, and analyses presenting *I*^2^ > 50% were considered as high heterogeneity [71]. Some reports suggest that meta-analysis with high heterogeneity should be omitted because the high variability among the included studies could compromise the reliability and clinical applicability [73, 74]. However, there is no consensus regarding the limit of acceptable heterogeneity for meaningful meta-analysis, and omitting meta-analysis results or excluding studies to reach homogeneity could prevent understanding of the real state of the literature [74–76]. Therefore, we reported meta-analyses with high heterogeneity and explored possible sources of heterogeneity by performing subgroup and meta-regression analyses for the meta-analyses including more than 10 studies [45].

For Q1, subgroup analyses included sex (females and male/female combined), assessment method, and task (static or dynamic); and meta-regression analysis included age as a potential moderator. For Q2, subgroup analyses included intervention characteristics (passive or exercise), comparator type (sham/no-intervention or exercise), design (parallel or crossover) and intervention characteristics (intervention targeting balance or non-specific for balance); and meta-regression analyses included age and treatment duration (weeks) as potential moderators. For Q3, subgroup analyses included comparator type (sham/no-intervention or exercise), and study aim (effect of balance or not specific for balance effects); and meta-regression analyses included age and treatment duration (weeks) as potential moderators.

The test proposed by Egger [45, 77] was used to evaluate the presence of publication bias for the meta-analyses including more than 10 studies [45]. When publication bias was detected (Egger’s test *p* ≤ 0.05), two sensitivity analyses were conducted to verify the impact of this bias: (i) trim-and-fill analyses in which effect sizes are imputed to balance the influence of small-study effects until funnel plot symmetry is reached [78]; and (ii) considering the limitations of the trim-and-fill approach [79–81], we also performed analyses by removing the outliers detected in the trim-and-fill analysis [82, 83].

### Level of Evidence

The level of evidence was assessed by two independent reviewers using the GRADE tool, and disagreements were resolved by a third reviewer. The level of GRADE evidence was downgraded if meta-analysis: (i) included > 25% of studies with high RoB (1 level) or only studies with high RoB (2 levels); (ii) were heterogeneous as assessed by *I*^2^ (> 50%) (1 level); (iii) did not include direct evidence related to the main questions, i.e. generalisation (1 level); (iv) included less than 100 participants per group (1 level); and (v) presented publication bias according to Egger’s test (*p* ≤ 0.05) (1 level) [47, 48, 84]. For analysis with less than 10 studies, publication bias was not considered [47, 48]. The evidence quality was classified as high (no downgraded level), moderate (downgraded 1 level), low (downgraded 2), or very low (downgraded ≥ 3 levels) [47, 48].

## Results

### Study Selection

From 15,436 records, 57 studies were included, of which 28 papers met the eligibility criteria for Q1, 23 papers for Q2 and 14 papers for Q3 (Fig. 1). Excluded studies during full-text screening are presented in Additional file 2 along with the reasons for exclusion. Meta-analyses for Q1 included 26 papers, for Q2 included 15 papers and for Q3 included 14 papers. Funnel plots are presented in Additional file 3, and detailed GRADE scores are presented in Additional file 4.

### Question 1: PFP Versus Control (Asymptomatic) for Balance Measures

**Fig. 2:**
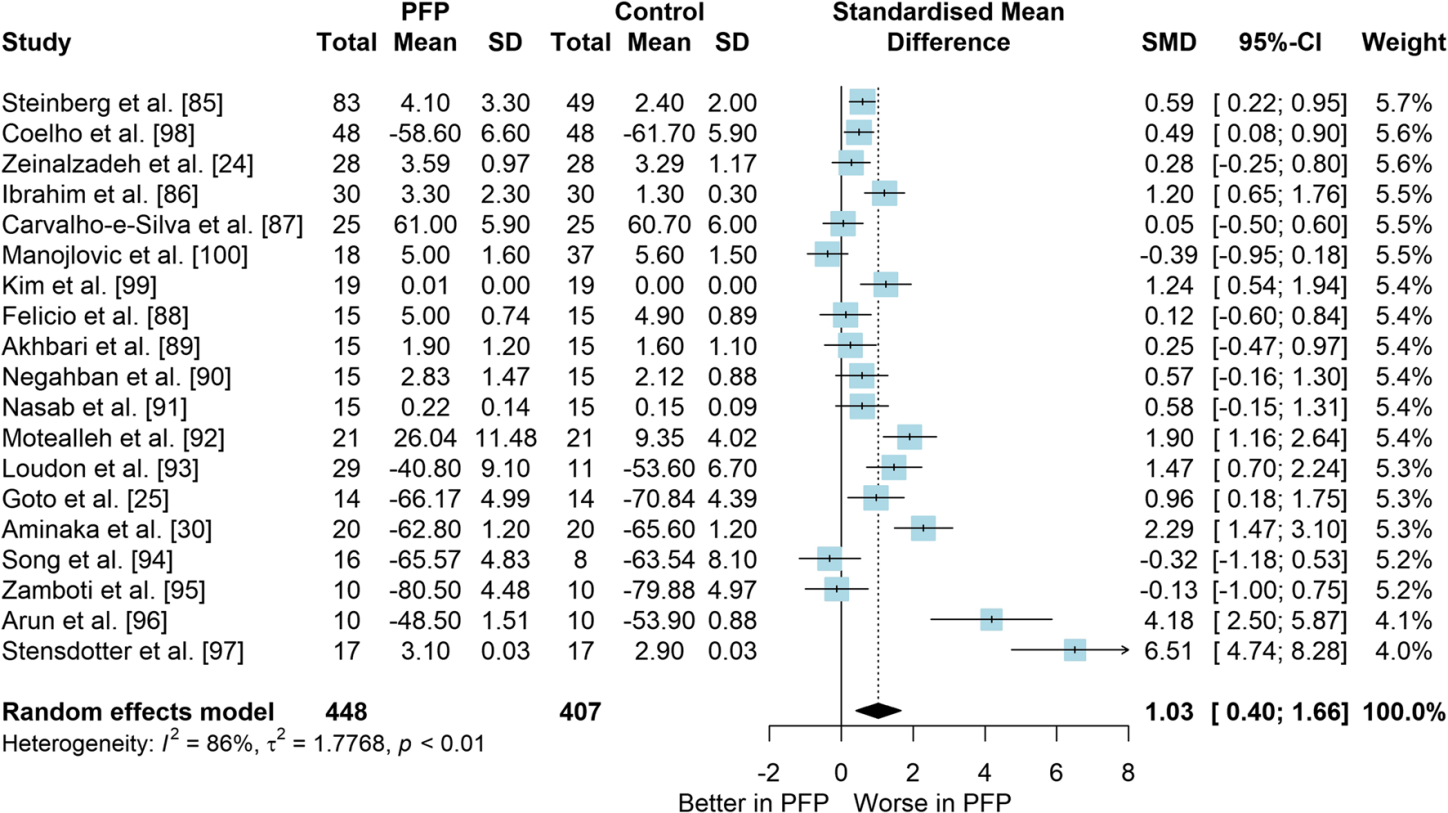
Anteroposterior postural stability indices—PFP versus Controls

**Fig. 3:**
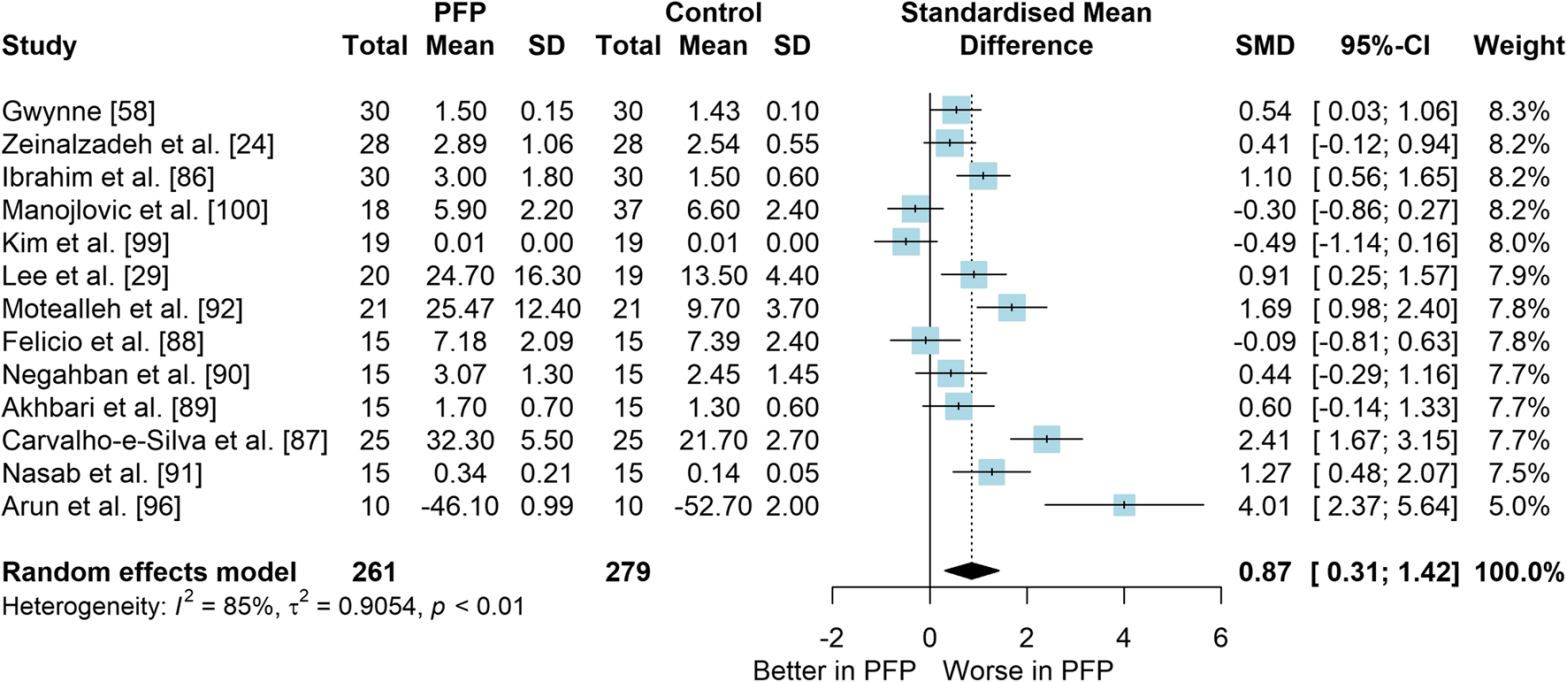
Mediolateral postural stability indices—PFP versus Controls

**Fig. 4:**
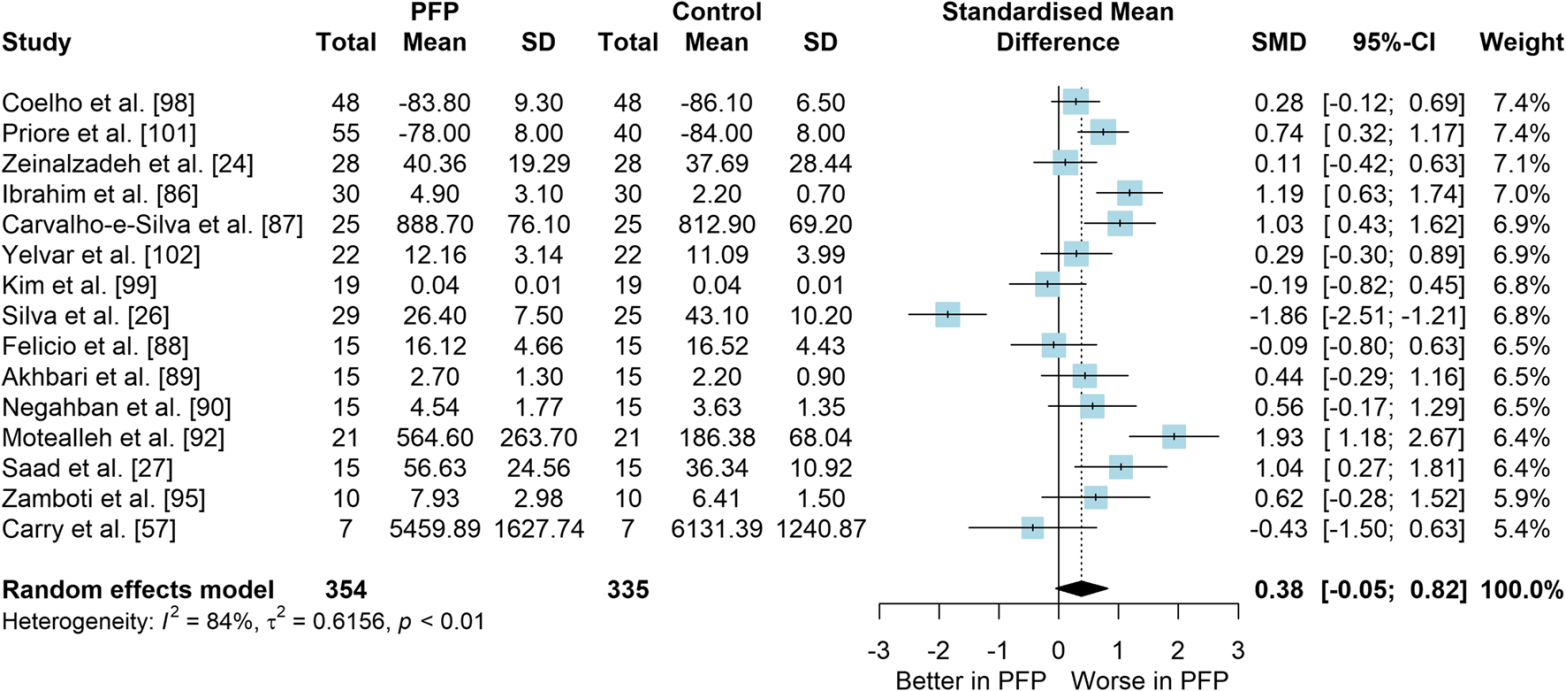
Overall postural stability indices—PFP versus Controls

**Fig. 5:**
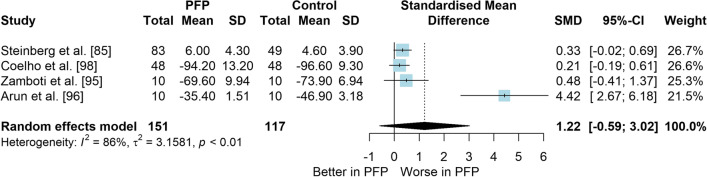
Posteromedial postural stability indices (SEBT)—PFP versus Controls

**Fig. 6:**
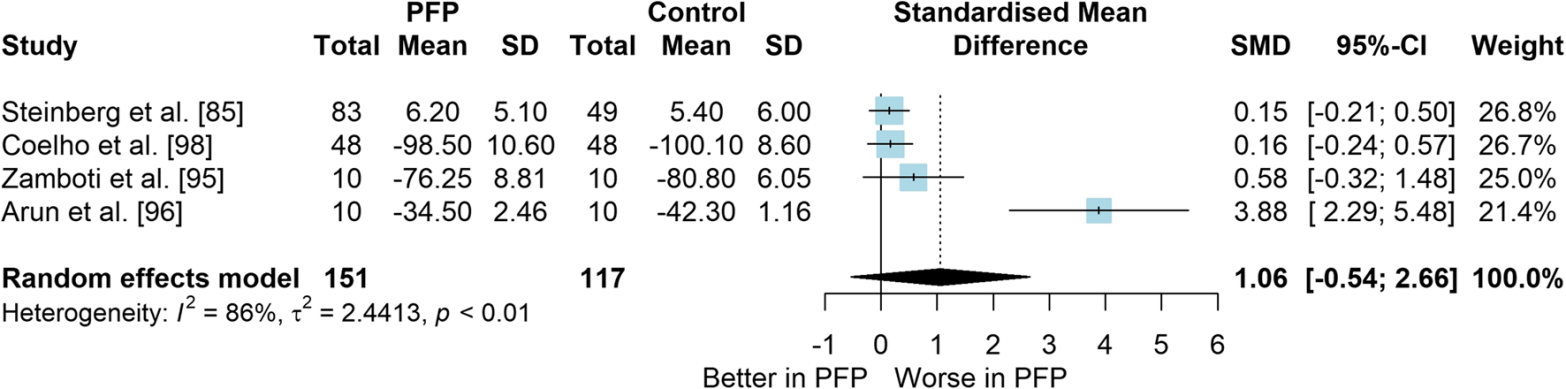
Posterolateral postural stability indices (SEBT)—PFP versus Controls

**Fig. 7:**
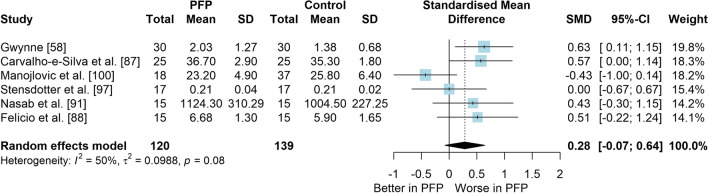
Anteroposterior centre of pressure velocity—PFP versus Controls

**Fig. 8:**
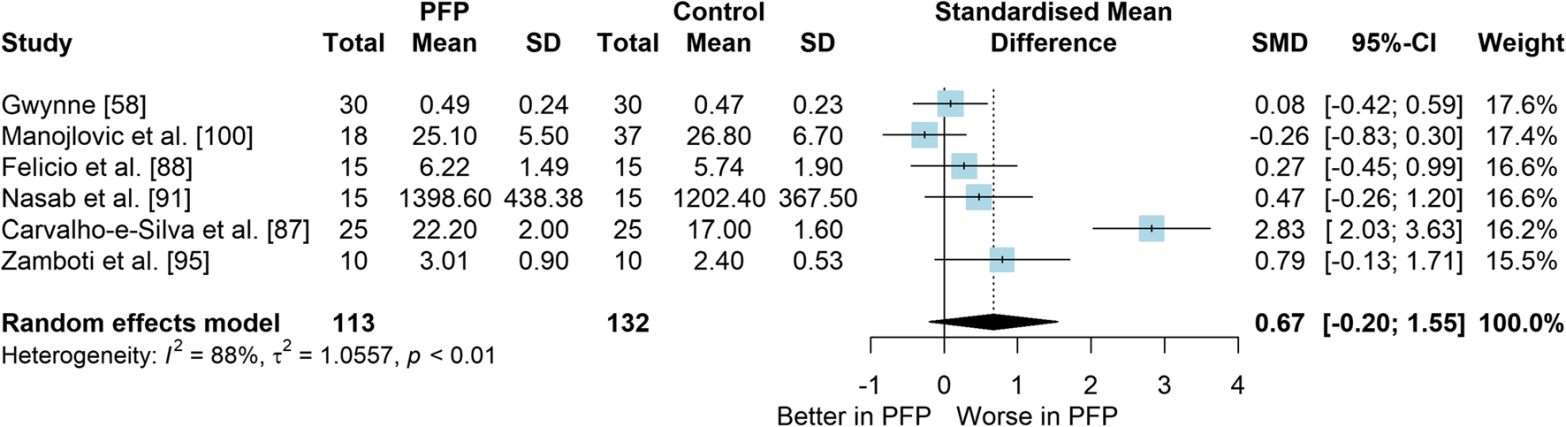
Mediolateral centre of pressure velocity—PFP versus Controls

**Fig. 9:**
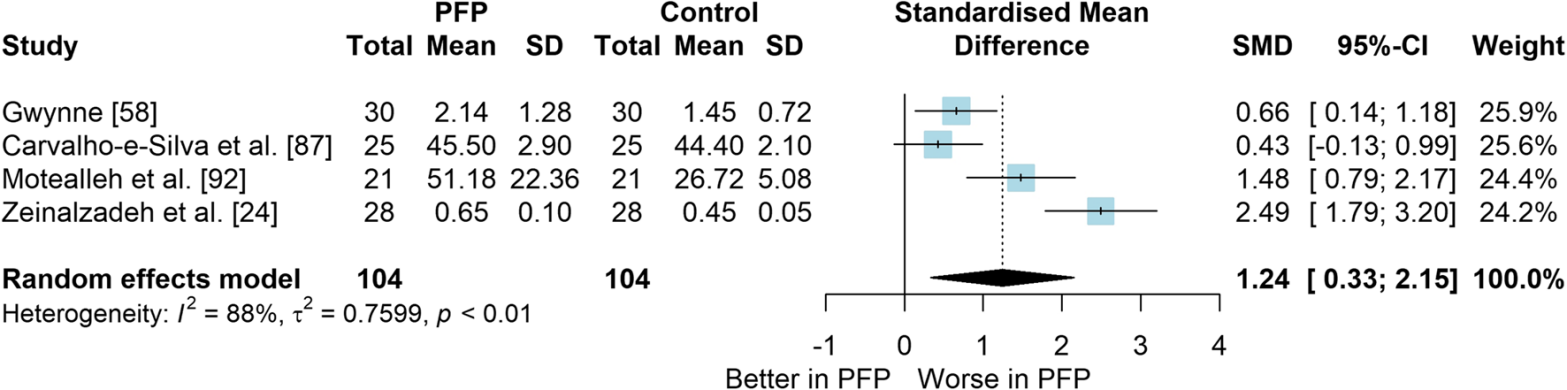
Overall centre of pressure velocity—PFP versus Controls

- *Study characteristics:* From 28 included studies, 679 people with PFP and 616 people without PFP were evaluated regarding balance performance (Additional file 5). Posturography (single- and double-legged stance), CoP behaviour, and SEBT-related tasks were used to assess balance performance; CoP behaviour was assessed during single-legged squat, step tasks, seated position, single-legged landing, single- and double-legged stance (Additional file 5).
- *Risk of bias:* The mean EIA score was 1.2 (0.3), with 75% of included studies presenting low RoB (*n* = 21), and 25% presenting high RoB (*n* = 7) (Additional file 6).
- *Anteroposterior (AP) postural stability (19 studies* [24, 25, 30, 85–100]*):* Very low level evidence indicated that people with PFP present worse AP balance with a large effect compared to controls (SMD 1.03, 95% CI 0.40–1.66; Fig. 2 and Table 1). Publication bias was detected (Egger’s test *p* = 0.018), and the sensitivity analyses indicated different results. Trim-and-fill analysis indicated a moderate and non-significant effect (low-level evidence, SMD 0.64, 95% CI − 0.23–1.52; Table 1) and by removing the outliers [96, 97], the analysis indicated a moderate and significant effect for worse balance in people with PFP (moderate level evidence, SMD 0.65, 95% CI 0.30–0.99; Table 1) (Additional file 3). Subgroup analyses indicated that the assessment method is a potential source of heterogeneity (Additional file 7). Meta-regression indicated that age is not a source of heterogeneity (Additional file 7).
- *Mediolateral (ML) postural stability (13 studies* [24, 29, 58, 86–92, 96, 99, 100]*):* Moderate level evidence indicated that people with PFP present worse ML balance with a large effect compared to controls (SMD 0.87, 95% CI 0.31–1.42; Fig. 3 and Table 1). The Egger’s test was not significant for publication bias (*p* = 0.059); however, as we found a marginal *p* value, we performed the sensitivity analyses. Trim-and-fill analysis (moderate level evidence, SMD 0.70, 95% CI 0.23–1.37; Table 1) and by removing the outlier [96] (moderate level evidence, SMD 0.69, 95% CI 0.23–1.16; Table 1) also indicated a moderate effect for worse balance in people with PFP (Additional file 3). Subgroup and meta-regression analyses did not indicate potential sources of heterogeneity (Additional file 7).
- *Overall postural stability (15 studies* [24, 26, 27, 57, 86–90, 92, 95, 98, 99, 101, 102]*):* Moderate level evidence indicated that there is no difference between people with and without PFP for overall balance (SMD 0.38, 95% CI − 0.05–0.82; Fig. 4 and Table 1). Publication bias was not detected (Egger’s test *p* = 0.813, Additional file 3). Subgroup analyses indicated that the sex is a potential source of heterogeneity (Additional file 7). Meta-regression indicated that age is not a source of heterogeneity (Additional file 7).
- *Posteromedial (PM) postural stability (4 studies* [85, 95, 96, 98]*):* Moderate level evidence indicated a very large and non-significant effect for lower reach during SEBT PM in people with PFP (SMD 1.22, 95% CI − 0.59–3.02; Fig. 5 and Table 1).
- *Posterolateral (PL) postural stability (4 studies* [85, 95, 96, 98]*):* Moderate level evidence indicated a large and non-significant effect for lower reach during SEBT PL in people with PFP (SMD 1.06, 95% CI − 0.54–2.66; Fig. 6 and Table 1).
- *AP CoP Velocity (6 studies* [58, 87, 88, 91, 97, 100]*):* Moderate level evidence indicated that there is no difference between people with and without PFP for AP CoP velocity (SMD 0.28, 95% CI − 0.07–0.64; Fig. 7 and Table 1).
- *ML CoP Velocity (6 studies* [58, 87, 88, 91, 95, 100]*):* Moderate level evidence indicated a moderate and non-significant effect for greater ML CoP velocity in people with PFP compared to controls (SMD 0.67, 95% CI − 0.20–1.55; Fig. 8 and Table 1).
- *Overall CoP Velocity (4 studies* [24, 58, 87, 92]*):* Moderate level evidence indicated that people with PFP present greater overall CoP velocity with a very large effect compared to controls (SMD 1.24, 95% CI 0.33–2.15; Fig. 9 and Table 1).
- *Influence of vision:* Felicio et al. [88] was the only study that exclusively assessed their participants with eyes closed (AP, ML and overall balance) and a sensitivity analysis was performed to verify the impact of pooling assessments performed with open and closed eyes for all meta-analyses. We concluded that the inclusion of the Felicio et al. [88] study did not impact the results.
- *Studies not included in meta-analysis*: Naserpour et al. [103] was the only study that evaluated the time to CoP stabilisation and reported statistical difference between groups (PFP group took a longer time to stabilise); and Stensdotter et al. [104] did not report their data in detail (Additional file 8).

**Table 1:** Meta-analytic results of the comparisons between people with and without patellofemoral pain on balance assessment (Q1)

Analysis	*n* PFP/Control	SMD (95% CI)	*p* value	*I*^2^ (%)	Level of Evidence	Egger’s test (*p* value)
AP direction (19 studies)	448/407	1.03 (0.40–1.66)	0.001	86	Very Low	0.018
Trim-and-fill analysis (2 imputed studies)		0.64 (− 0.23–1.52)	0.151	89	Low	0.746
Removing outliers (2 deleted studies)		0.65 (0.30–0.99)	< 0.001	77	Moderate	0.460
ML direction (13 studies)	261/279	0.87 (0.31–1.42)	0.002	85	Moderate	0.059
Trim-and-fill analysis (1 imputed study)		0.70 (0.23–1.37)	0.043	87	Moderate	0.641
Removing outliers (1 deleted study)		0.69 (0.23–1.16)	< 0.001	77	Moderate	0.292
Overall balance (15 studies)	354/335	0.38 (− 0.05–0.82)	0.083	84	Moderate	0.813
PM direction (SEBT; 4 studies)	151/117	1.22 (− 0.59–3.02)	0.187	86	Moderate	NA
PL direction (SEBT; 4 studies)	151/117	1.06 (− 0.54–2.66)	0.194	86	Moderate	NA
AP CoP velocity (6 studies)	120/139	0.28 (− 0.07–0.64)	0.121	50	Moderate	NA
ML CoP velocity (6 studies)	113/132	0.67 (− 0.20–1.55)	0.130	88	Moderate	NA
Overall CoP velocity (4 studies)	104/104	1.24 (0.33–2.15)	0.007	88	Moderate	NA

*AP* anteroposterior, *CoP* centre of pressure, *ML* mediolateral, *NA* not applicable, *PFP* patellofemoral pain, *PM* posteromedial, *PL* posterolateral, *SEBT* Star Excursion Balance Test, *SMD* standardised mean difference

### Question 2: Interventions Targeting Balance Improvements for People with PFP

**Fig. 10:**
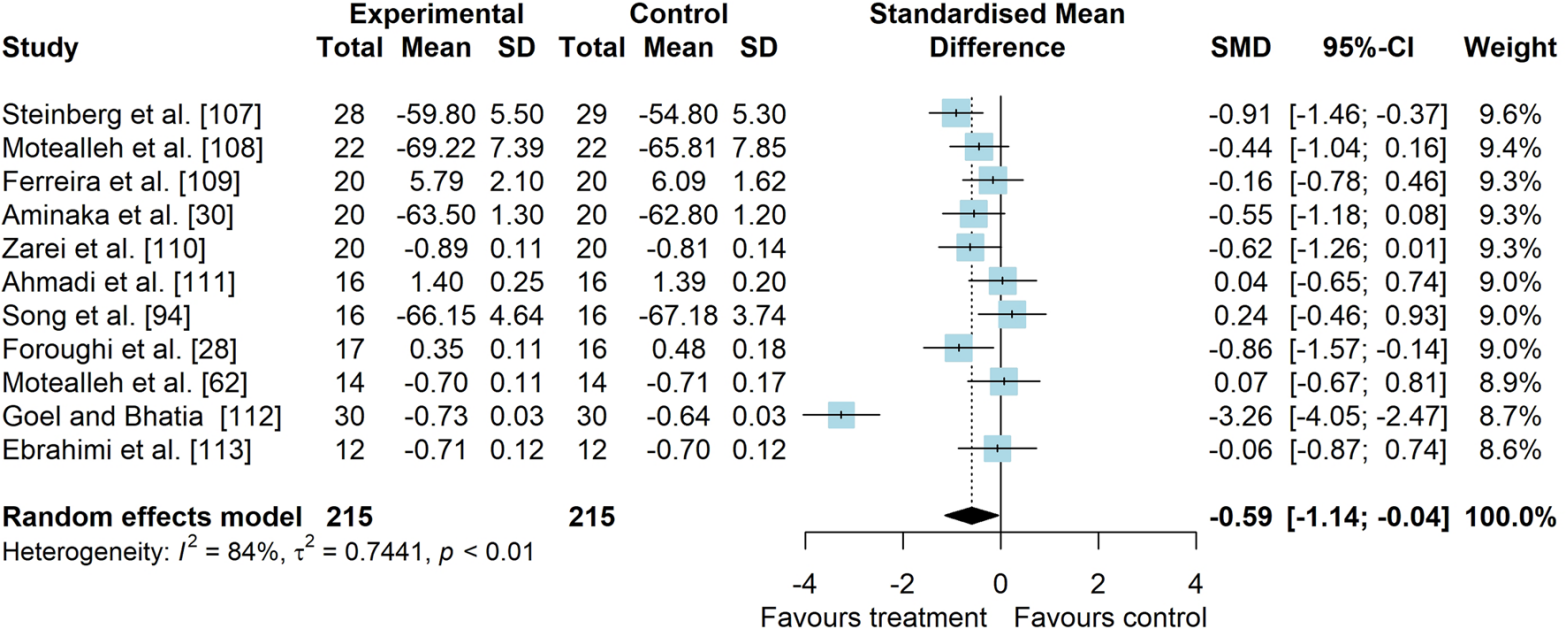
Effect of conservative interventions on the anteroposterior postural stability compared to control interventions

**Fig. 11:**
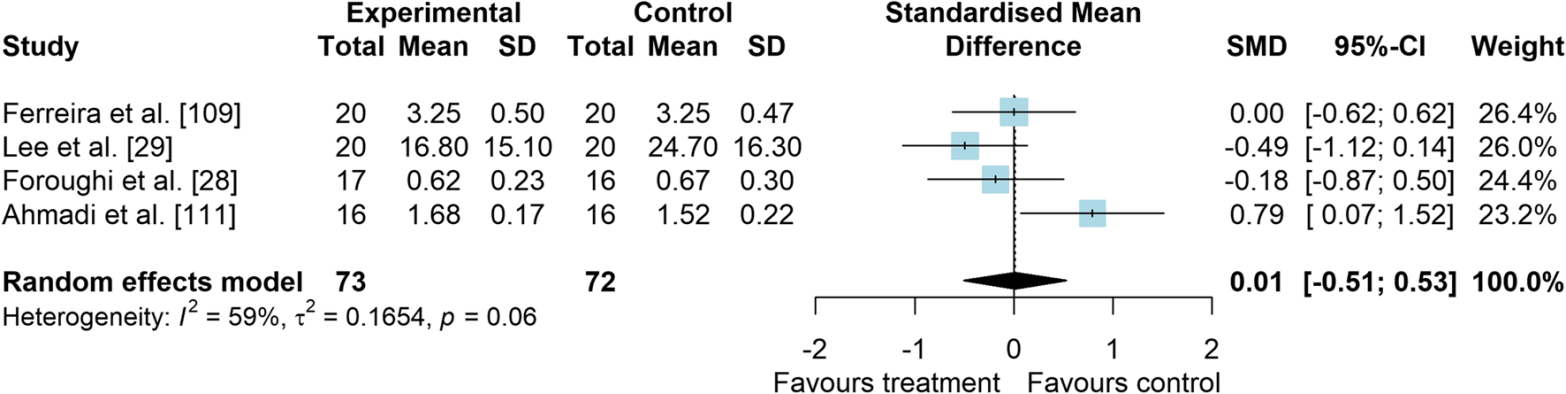
Effect of conservative interventions on the mediolateral postural stability compared to control interventions

**Fig. 12:**
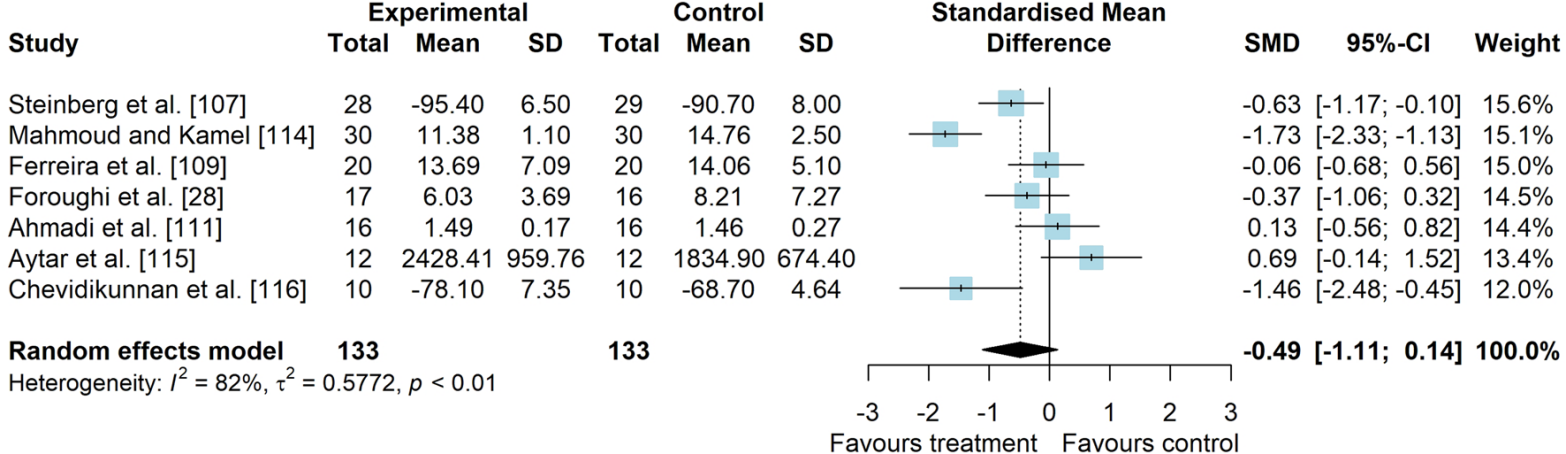
Effect of conservative interventions on the overall postural stability compared to control interventions

**Fig. 13:**
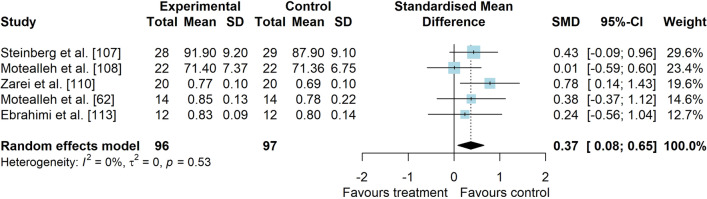
Effect of conservative interventions on the posteromedial postural stability (SEBT) compared to control interventions

**Fig. 14:**
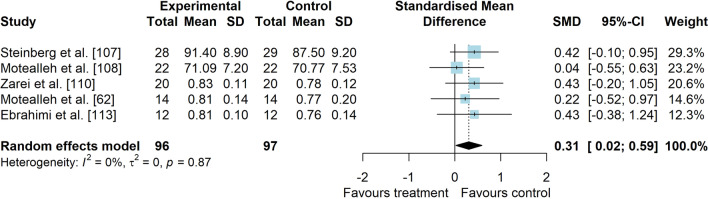
Effect of conservative interventions on the posterolateral postural stability (SEBT) compared to control interventions

- *Study characteristics:* The studies of Maryan et al. [105] and Maryan et al. [106] reported identical data and were considered as one study for analyses. From 23 included studies, 755 people with PFP were included (Additional file 5). SEBT-related tasks, posturography (single- and double-legged stance) and CoP behaviour were applied to assess balance performance. CoP behaviour was assessed during single-legged squat, step tasks, seated position and single-legged stance. Interventions included neurofeedback, taping, exercises, braces, manual therapy, dry needling, and virtual reality (Additional file 5).
- *Risk of bias:* The mean PEDro score was 2.9 (1.4), with 50% presenting high RoB (*n* = 11), 36% moderate RoB (*n* = 8), and 14% presenting low RoB (*n* = 3) (Additional file 6).
- *AP postural stability (11 studies* [28, 30, 62, 94, 107–113]*):* low-level evidence indicated that interventions are moderately effective to improve AP balance compared to control interventions (SMD 0.59, 95% CI 0.04–1.14, Fig. 10 and Table 2). Publication bias was not detected (Egger’s test *p* = 0.681, Additional file 3). Subgroup analyses indicated that the type of comparator, type of experimental intervention, and design are potential sources of heterogeneity (Additional file 7). Meta-regression indicated that age and treatment duration are not sources of heterogeneity (Additional file 7).
- *ML postural stability (4 studies* [28, 29, 109, 111]*):* very low level evidence indicated that interventions are not effective to improve ML balance compared to control interventions (SMD 0.01, 95% CI − 0.51–0.53, Fig. 11 and Table 2).
- *Overall postural stability (7 studies* [28, 107, 109, 111, 114–116]*):* low-level evidence indicated a non-significant small effect in favour of interventions compared to control interventions (SMD 0.49, 95% CI − 0.14–1.11, Fig. 12 and Table 2).
- *PM postural stability (5 studies* [62, 107, 108, 110, 113]*):* moderate level evidence indicated that interventions lead to small improvement for SEBT PM (SMD 0.37, 95% CI 0.08–0.65) compared to control interventions (Fig. 13 and Table 2).
- *PL postural stability (5 studies* [62, 107, 108, 110, 113]*):* moderate level evidence indicated that interventions lead to small improvement for SEBT PL (SMD 0.31, 95% CI 0.02–0.59) compared to control interventions (Fig. 14 and Table 2).
- *Studies not included in meta-analysis*: Loudon et al. [31] was the only study that evaluated intervention effects using the balance and reach test, and reported that exercise is able to improve balance in people with PFP; Miller et al. [117], and Maryam et al. [105, 106] did not report their data in detail; and the studies by Demirci et al. [118], Ojaghi et al. [119], Sinaei et al. [120] and Fang et al. [121] compared two types of experimental interventions without a control condition (Additional file 8).

**Table 2:** Meta-analytic results of the effects of experimental interventions versus control interventions on balance for people with patellofemoral pain (Q2)

Analysis	*n* Exp/Con	SMD (95% CI)	*p* value	*I*^2^ (%)	Level of Evidence
AP direction^a^ (11 studies)	215/215	0.59 (0.04–1.14)	0.038	84	Low
ML direction (4 studies)	73/72	0.01 (− 0.51–0.53)	0.966	59	Very Low
Overall direction (7 studies)	133/133	0.49 (− 0.12–1.09)	0.126	82	Low
PM direction (SEBT; 5 studies)	96/97	0.37 (0.08–0.65)	0.012	0	Moderate
PL direction (SEBT; 5 studies)	96/97	0.31 (0.02–0.59)	0.034	0	Moderate

*AP* anteroposterior, *Con* control group, *Exp* experimental group, *ML* mediolateral, *PM* posteromedial, *PL* posterolateral, *SEBT* Star Excursion Balance Test, *SMD* standardised mean difference

^a^No publication bias detected—Egger’s test: *p* = 0.681

### Question 3: Balance Interventions Targeting Pain and Function for People with PFP

**Fig. 15:**
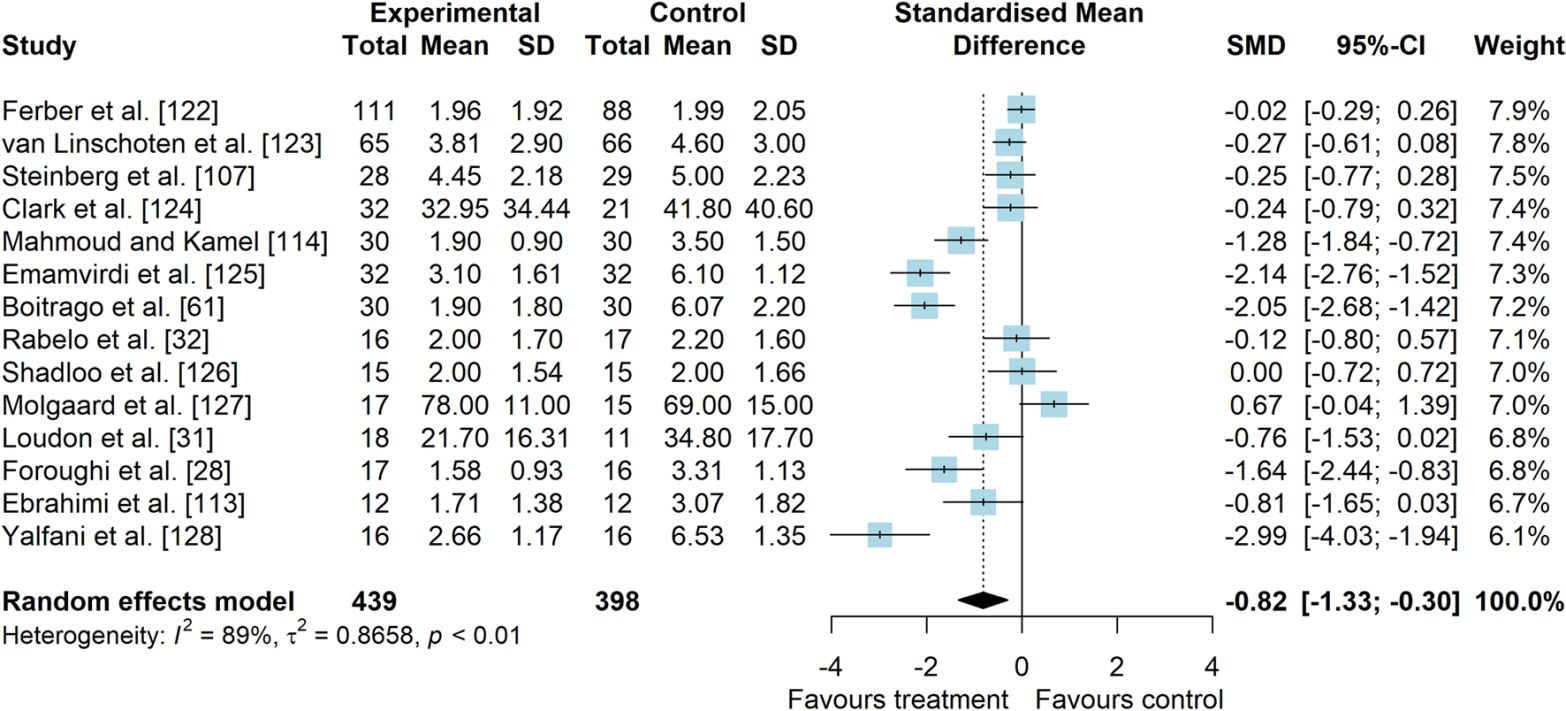
Effect of balance interventions on pain compared to non-balance interventions

**Fig. 16:**
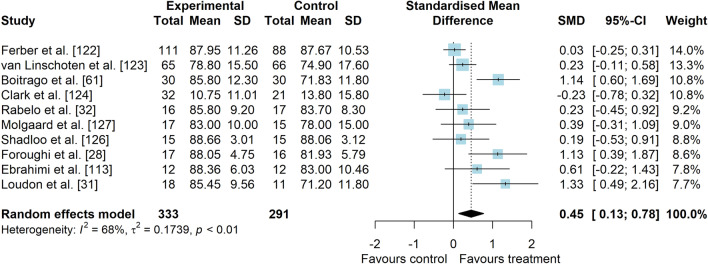
Effect of balance interventions on function (PROMs) compared to non-balance interventions

**Fig. 17:**
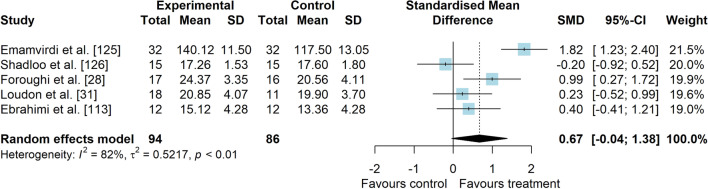
Effect of balance interventions on function (functional tests) compared to non-balance interventions

- *Study characteristics:* Of 14 included studies, 907 people with PFP were included (Additional file 5). Visual analogue scale (VAS), numeric pain rating scale (NPRS), and Knee injury and Osteoarthritis Outcome Score (KOOS) subscale pain were used to assess pain level. Anterior Knee Pain Scale (AKPS), Knee Outcome Survey—Activities of Daily Living Scale (KOS-ADLS), Western Ontario and McMaster Universities Osteoarthritis Index (WOMAC), KOOS subscales and functional tests were used to assess functional status. A description of balance interventions is presented in Additional file 5.
- *Risk of bias:* The mean PEDro score was 3.6 (1.8), with 50% presenting low RoB (*n* = 7), 14% moderate RoB (*n* = 2), and 36% presenting high RoB (*n* = 5) (Additional file 6).
- *Pain data (14 studies* [28, 31, 32, 61, 107, 113, 114, 122–128]*):* low-level evidence indicated that balance interventions were largely effective to improve pain compared to non-balance interventions (SMD 0.82, 95% CI 0.30–1.33, Fig. 15 and Table 3). The Egger’s test was not significant for publication bias (*p* = 0.054); however, as we found a marginal *p* value, we performed the sensitivity analyses which showed different results. Trim-and-fill analysis indicated that balance interventions have no effect on pain (low-level evidence, SMD 0.38, 95% CI − 0.28–1.03; Table 3) and the analysis by removing the outliers [61, 125, 128] indicated that balance interventions have a small effect in improving pain in people with PFP compared to non-balance interventions (low-level evidence, SMD 0.40, 95% CI 0.04–0.76; Table 3) (Additional file 3). Subgroup and meta-regression analyses did not indicate possible sources of heterogeneity (Additional file 7).
- *Function data*: considering patient-reported outcome measures (10 studies [28, 31, 32, 61, 113, 122–124, 126, 127]), moderate level evidence indicated that balance interventions have a small effect in improving function compared to non-balance interventions (SMD 0.45, 95% CI 0.13–0.78, Fig. 16 and Table 3). Subgroup and meta-regression analyses did not indicate possible sources of heterogeneity (Additional file 7). Considering functional tests (5 studies [28, 31, 113, 125, 126]), very low level evidence indicated that balance interventions have a moderate and non-significant effect in improving function compared to non-balance interventions (SMD 0.67, 95% CI − 0.04–1.38, Fig. 17 and Table 3).

**Table 3:** Meta-analytic results of the effects of balance exercises versus non-balance interventions on pain and function for people with patellofemoral pain (Q3)

Analysis	*n* Exp/Con	SMD (95% CI)	*p* value	*I*^2^ (%)	Level of Evidence	Egger’s test (*p* value)
Pain (14 studies)	439/398	0.82 (0.30–1.33)	0.002	89	Low	0.054
Trim-and-fill analysis (3 imputed studies)		0.38 (− 0.28–1.03)	0.259	93	Low	0.613
Removing outliers (3 deleted studies)		0.40 (0.04–0.76)	0.028	73	Low	0.242
Function (PROMs—10 studies)	333/291	0.45 (0.13–0.78)	0.006	68	Moderate	0.082
Function (tests—5 studies)	94/95	0.67 (− 0.04–1.38)	0.065	82	Very Low	NA

*Con* control group, *Exp* experimental group, *PROMs* patient-reported outcome measures, *NA* not applicable, *SMD* standardised mean difference

## Discussion

### PFP Versus Control (Asymptomatic) on Balance (Q1)

The cross-sectional data revealed ambiguous results because most meta-analyses reported non-significant results, but the meta-analyses with more included studies reported impairments in the balance of people with PFP. Most meta-analyses (six out of eight) reported non-significant results based on moderate-level evidence, indicating that balance is not impaired in people with PFP. However, the non-significant results raise questions regarding the actual presence of balance impairments in the population with PFP. The non-significant results for SEBT PM and PL, and ML CoP velocity were obtained from meta-analyses with few included studies (*n* = 4–6), with magnitude effects varying from moderate to very large for worse balance in people with PFP, which does not the support a consistent conclusion.

Considering the results from the three meta-analyses with more studies included (*n* = 13–19 studies), there are some circumstances which require attention for the interpretation. The results for ML postural stability reported more coherent findings that people with PFP have impairments in ML balance based on moderate-level evidence. The results for AP postural stability are based on very low level evidence and seem to be highly influenced by the small-study effect, suggesting that publication bias interfered with the results. Although the trim-and-fill analysis reported non-significant results, in the analysis by removing the outliers, we could observe a significant result as the original analysis (moderate effect), and the level of evidence increased to moderate. The findings for overall postural stability seem to be highly affected by one study which likely influenced the meta-analysis towards a non-significant result. Although the publication bias analysis did not indicate outliers, only one study presented results suggesting that people with PFP have less overall balance sway [26] with the other 18 studies presenting that people with PFP have a worse overall balance or no difference compared to controls (Fig. 4). Therefore, based on the present results, we cannot affirm that people with PFP have impairments in balance compared to asymptomatic people. However, there are some findings suggesting that balance is a physical factor likely altered in people with PFP. Our results show the need for more high-quality with large sample size investigations in order to confirm whether balance impairments are present in the PFP population.

Different aspects affecting people with PFP may compromise postural control, such as impairments in central control [129], proprioception [130], muscle activity [131] or muscle capacity [65, 132]. Previous studies reported that postural control in people with PFP is correlated with knee muscle strength [23], hip muscle strength [87, 100] and knee proprioception [98]. In contrast, studies reported controversial results on the correlation between postural control and pain [23, 101, 102]. Nevertheless, little is known about the cause–consequence relationship between PFP and postural control. A recent study indicated that balance impairments could be a risk factor for PFP development [133]. Contrarily, some experimental research reported that induced knee pain impairs balance and quadriceps coordination [134, 135]. Therefore, a better understanding of how balance is affected as PFP develops could help clinicians in their decisions regarding possible interventions aiming at treating or preventing PFP [136].

### Interventions Addressing Balance in People with PFP (Q2)

This is the first review pooling information on balance from people with PFP and interestingly, 23 randomised controlled trials used balance measures as an outcome to determine the efficacy of their interventions. Based on the meta-analyses for Q2, we cannot affirm that balance is a modifiable outcome in people with PFP. The results present a number of issues which makes the conclusion fragile. The positive results for improvements on AP, PM and PL balance include a confidence interval which does not warrant concluding a clinical significance of these interventions [137]. Additionally, the AP balance result was likely influenced by a study with a very large effect in favour of the experimental intervention (SMD = 3.26) [112]. The non-significant results for ML and overall balance and the low to very low level evidence provide further information to question the effectiveness of interventions to improve balance in people with PFP.

We can affirm that the inconclusive results are related to the diversity of interventions, including neurofeedback [111], taping [30, 94, 105, 106, 109, 112, 115, 117–120], exercises [28, 31, 62, 107, 114, 116, 121], braces [29], manual therapy [108, 117, 118], dry needling [110] and virtual reality [113]. Few studies included exercises addressing specifically balance deficits [28, 31, 107, 114]. The study by Steinberg et al. [107] included single-legged ballet-related exercises, and the study by Foroughi et al. [28] included an exercise in which the participants were required to maintain balance on an unstable seat apparatus; and the subgroup analysis including these two studies showed a large effect in favour of the interventions on AP balance (very low level evidence). The study by Mahmoud and Kamel [114] included a progressive balance exercise programme and presented a very large effect in favour of interventions in improving overall balance (study with moderate RoB). The study by Loudon et al. [31] included single-legged stance and reach tasks and reported improvements in the balance and reach test (study with high RoB). Therefore, we may infer that balance is a potential modifiable factor in people with PFP, but the lack of specific and high-quality studies does not allow a clear conclusion. Further investigation is needed to ascertain whether interventions are effective to improve balance in people with PFP.

### Balance Interventions on Pain and Function in People with PFP (Q3)

The results for Q3 suggest that interventions which included balance exercises are not clearly effective for function improvement or pain reduction. The effect on function measured using patient-reported outcome measures was small and included a confidence interval which does not justify concluding clinical relevance [137]. Additionally, no significant effect was observed for function measured using functional tests. For pain reduction, the meta-analysis reported a large effect in favour of interventions including balance exercises, based on low-level evidence and including a confidence interval with the lower limit that most clinicians and researchers would be considered to be not clinically significant [137].

The literature reports strong evidence supporting the effectiveness of some interventions addressing pain and function in people with PFP, such as hip- and knee-targeted exercise therapy [7, 12]. Comparing the evidence level of previous results and our findings, we could suggest that balance exercises are less essential for PFP rehabilitation. Nonetheless, beyond the fact that subgroup analyses were performed for heterogeneity investigation, their results suggest the importance of multimodal exercise programmes, including balance exercises as one component to reduce pain in people with PFP. Multimodal programmes that included balance exercises moderately reduced pain compared to control interventions without balance exercises. Additionally, the subgroup analysis pooling studies that specifically aimed to verify the effects of balance reported a very large effect in favour of interventions. However, we should consider these results with caution; if the pain explains impairments in balance, targeting balance on its own might not be relevant. Therefore, the inconclusive results about the effects of balance intervention on pain and function do not justify clinical application, but the results encourage further investigations in the field.

### Limitations

An important limitation of the findings is the heterogeneity which was present in all meta-analyses. It suggests that the diversity in participants’ characteristics, interventions or methodological aspects exceeds the diversity expected by chance and likely influences the results [45]. The subgroup analysis indicated some potential factors which may explain the heterogeneity, such as the assessment method and sex for Q1; and the type of comparator, the design and specificity of interventions for Q2. Even with these factors, we could not conclude which factors strongly influence the statistical heterogeneity. Therefore, other factors should be explored, such as pain [20, 21] and body mass index [138, 139] which may have an important role in moderating postural control. The study by Yelvar et al. [102] reported that pain and body mass index are moderately correlated with postural control in people with PFP. We intended to perform meta-regression analyses to verify whether pain and body mass index could explain the heterogeneity; however, the included studies poorly reported these variables (Additional file 5) which prevented such analysis. We may speculate that the heterogeneity reflects the multifactorial aspect of PFP, along with a possible high level of heterogeneity in many characteristics among this population that could affect results. Additionally, other systematic reviews investigating the influence of balance on different populations, such as older people [34, 140] and people with chronic ankle instability [141, 142], also reported heterogeneity in their results, which suggest that heterogeneity may be inherent in this topic. Nevertheless, the high heterogeneity of the present findings is important and should be considered for the interpretation of the results. Another limitation is that some results are based on low or very low level evidence which compromises the trustworthiness of the reported effects. Also, for some meta-analyses, we could not assess the presence of publication bias due to the number of included studies. Therefore, the present results should be interpreted with caution and additional studies with low RoB and homogeneous data may change our conclusions.

## Conclusions

People with PFP likely present balance impairments compared to asymptomatic people. There was insufficient evidence to support the efficacy of interventions to improve or modify balance in people with PFP. Also, there was insufficient evidence to support the efficacy of balance exercises to improve pain and function in people with PFP.

## Supplementary Information

**Additional file 1.** Strategy searches.

**Additional file 2.** Excluded studies.

**Additional file 3.** Funnel plots.

**Additional file 4.** GRADE description.

**Additional file 5.** Study characteristics.

**Additional file 6.** Risk of bias assessment.

**Additional file 7.** Subgroup and meta-regression analysis.

**Additional file 8.** Data of the included studies.

## Data Availability

The data sets that support the findings of this study are presented in the supplementary materials and are also available upon request.

## Abbreviations

AP: Anteroposterior
CI: Confidence interval
CoP: Centre of pressure
EIA: Epidemiological appraisal instrument
GRADE: Grading of recommendations assessment, development and evaluation
ML: Mediolateral
PEDro: Physiotherapy evidence database
PFP: Patellofemoral pain
PL: Posterolateral
PM: Posteromedial
RoB: Risk of bias
SEBT: Star excursion balance test
SMD: Standardised mean differences
