# Supplementary material for: Is Postural Control Affected in People with Patellofemoral Pain and Should it be Part of Rehabilitation? A Systematic Review with Meta-analysis
Source: Sports Med Open. 2022 Dec 12;8:144. doi: 10.1186/s40798-022-00538-4 (PMC9742077; doi:10.1186/s40798-022-00538-4)
Supplement: Supplementary file 1 — Additional file 1. Strategy searches. [file 40798_2022_538_MOESM1_ESM.pdf]

Additional file 1. Strategy seaches for Medline (Ovid), Embase (Elsevier), CINAHL (EBSCO), SPORTDiscuss (EBSCO), Web of Science and Cochrane Library.

Medline for Question 1 (via Ovid)

1.	Patellofemoral Joint/
2.	Patella/
3.	Patellofemoral Pain Syndrome/
4.	Chondromalacia Patellae/
5.	(anterior knee pain).tw
6.	((patell* or femoropatell* or femoro-patell* or retropatell*) adj2 (pain or syndrome or dysfunction)).tw
7.	((chondromalac* or chondropath* or chondrosis) adj2 (knee* or patell* or femoropatell* or femoro-patell* or retropatell*)).tw
8.	((lateral compression or lateral facet or lateral pressure or odd facet) adj2 (syndrome or pain)).tw
9.	or/1-8
10.	Postural Balance/
11.	Proprioception/
12.	(balanc* or equilibrium or postur*).mp
13.	((cent*) adj2 (pressure)).mp
14.	((postur* or $motor or neuromuscular) adj (sway or stabili* or instabili* or control* or oscillat* or perturbation*)).mp
15.	((single or one) adj2 (balance or stance or stabili*)).mp
16.	or/10-15
17.	9 and 16
18.	limit 17 to human

CINAHL for Question 1 (via EBSCO)

1.	MM “Patella”
2.	MM “Patellofemoral Pain Syndrome”
3.	MM “Chondromalacia Patella”
4.	(anterior knee pain)
5.	((patell* OR femoropatell* OR femoro-patell* OR retropatell*) N2 (pain OR syndrome OR dysfunction))
6.	((chondromalac* OR chondropath* OR chondrosis) N2 (knee* OR patell* OR femoropatell* OR femoro-patell* OR retropatell*))
7.	((lateral compression OR lateral facet OR lateral pressure OR odd facet) N2 (syndrome OR pain))
8.	or/1-7
9.	MM “Balance, Postural”
10.	MM “Proprioception”
11.	(balanc* OR equilibrium OR postur*)
12.	((cent*) N2 (pressure))
13.	((postur* OR $motor OR neuromuscular) N1 (sway OR stabili* OR instabili* OR control* OR oscillat* OR perturbation))
14.	((single OR one) N2 (balance OR stance OR stabili*))
15.	or/9-14

16.	8 and 15
17.	limit 16 to human

SPORTDiscus for Question 1 (via EBSCO)

1.	DE “PATELLOFEMORAL joint”
2.	DE “PATELLA”
3.	DE “PLICA syndrome”
4.	DE “PATELLOFEMORAL joint injuries”
5.	DE “CHONDROMALACIA patellae”
6.	(anterior knee pain)
7.	((patell* OR femoropatell* OR femoro-patell* OR retropatell*) N2 (pain OR syndrome OR dysfunction))
8.	((chondromalac* OR chondropath* OR chondrosis) N2 (knee* OR patell* OR femoropatell* OR femoro-patell* OR retropatell*))
9.	((lateral compression OR lateral facet OR lateral pressure OR odd facet) N2 (syndrome OR pain))
10.	OR/1-9
11.	DE “POSTURE disorders”
12.	DE “BALANCE disorders”
13.	DE “PROPRIOCEPTION”
14.	(balanc* OR equilibrium OR postur*)
15.	((cent*) N2 (pressure))
16.	((postur* OR $motor OR neuromuscular) N1 (sway OR stabili* OR instabili* OR control* OR oscillat* OR perturbation))
17.	((single OR one) N2 (balance OR stance OR stabili*))
18.	or/11-17
19.	10 and 18

Web of Science for Question 1

1.	(patellofemoral OR patella)
2.	(anterior knee pain)
3.	((patell* OR femoropatell* OR femoro-patell* OR retropatell*) NEAR/2 (pain OR syndrome OR dysfunction))
4.	((chondromalac* OR chondropath* OR chondrosis) NEAR/2 (knee * OR patell* OR femoropatell* OR femoro-patell* OR retropatell*))
5.	((compression OR facet OR pressure OR odd) NEAR/2 (syndrome OR pain))
6.	or/1-5
7.	(balanc* OR equilibrium OR postur* OR proprioception)
8.	((cent*) NEAR/2 (pressure))
9.	((postur* OR motor OR neuromuscular) NEAR/1 (sway OR stabili* OR instabili* OR control* OR oscillat* OR perturbation))
10.	((single OR one) NEAR/2 (balance OR stance OR stabili*))
11.	or/7-10
12.	#6 and #11
13.	TS=Animal
14.	#12 not #13

Embase for Question 1 (via Elsevier)

1.	‘Patellofemoral Joint’:de
2.	‘Patella’:de
3.	‘Patellofemoral Pain Syndrome’:de
4.	‘Patella chondromalacia’:de
5.	‘anterior knee pain’:ti,ab
6.	((patell* OR femoropatell* OR femoro-patell* OR retropatell*) NEAR/2 (pain OR syndrome OR dysfunction)): ti,ab
7.	((chondromalac* OR chondropath* OR chondrosis) NEAR/2 (knee* OR patell* OR femoropatell* OR femoro-patell* OR retropatell*)):ti,ab
8.	((‘lateral compression’ OR ‘lateral facet’ OR ‘lateral pressure’ OR ‘odd facet’) NEAR/2 (syndrome OR pain)):tw
9.	or/1-8
10.	‘Body equilibrium’:de
11.	‘Proprioception’:de
12.	(balanc* OR equilibrium OR postur*):ti,ab
13.	((cent*) NEAR/2 (pressure)):ti,ab
14.	((postur* OR $motor OR neuromuscular) NEAR/1 (sway OR stabili* OR instabili* OR control* OR oscillat* OR perturbation*)):ti,ab
15.	((single OR one) NEAR/2 (balance OR stance OR stabili*)):ti,ab
16.	or/10-15
17.	9 and 16
18.	limit 17 to human

Cochrane Library for Question 1

1.	MeSH descriptor: [Patellofemoral Joint] this term only
2.	MeSH descriptor: [Patella] this term only
3.	MeSH descriptor: [Patellofemoral Pain Syndrome] this term only
4.	MeSH descriptor: [Chondromalacia Patellae] this term only
5.	(anterior knee pain)
6.	((patell* or femoropatell* or femoro-patell* or retropatell*) NEAR/2 (pain or syndrome or dysfunction))
7.	((chondromalac* or chondropath* or chondrosis) NEAR/2 (knee* or patell* or femoropatell* or femoro-patell* or retropatell*))
8.	((lateral compression or lateral facet or lateral pressure or odd facet) NEAR/2 (syndrome or pain))
9.	#1 OR #2 OR #3 OR #4 OR #5 OR #6 OR #7 OR #8
10.	MeSH descriptor: [Postural Balance] this term only
11.	MeSH descriptor: [Proprioception] this term only
12.	(balanc* or equilibrium or postur*)
13.	((cent*) NEAR/2 (pressure))
14.	((postur* or $motor or neuromuscular) NEAR/1 (sway or stabili* or instabili* or control* or oscillat* or perturbation*))
15.	((single or one) NEAR/2 (balance or stance or stabili*))
16.	#10 OR #11 OR #12 OR #13 OR #14 OR #15
17.	#9 AND #16

Medline for Questions 2 and 3 (via Ovid)

1.	Patellofemoral Joint/
2.	Patella/
3.	Patellofemoral Pain Syndrome/
4.	Chondromalacia Patellae/
5.	(anterior knee pain).tw
6.	((patell* or femoropatell* or femoro-patell* or retropatell*) adj2 (pain or syndrome or dysfunction)).tw
7.	((chondromalac* or chondropath* or chondrosis) adj2 (knee* or patell* or femoropatell* or femoro-patell* or retropatell*)).tw
8.	((lateral compression or lateral facet or lateral pressure or odd facet) adj2 (syndrome or pain)).tw
9.	or/1-8
10.	Postural Balance/
11.	Proprioception/
12.	Rehabilitation/
13.	(balanc* or equilibrium or postur*).mp
14.	((cent*) adj2 (pressure)).mp
15.	((postur* or $motor or neuromuscular) adj (sway or stabili* or instabili* or control* or oscillat* or perturbation*)).mp
16.	((single or one) adj2 (balance or stance or stabili*)).mp
17.	(exercis* or train* or program* or protocol or physiotherapy or physical therap*).mp
18.	or/10-17
19.	Clinical trials as topic/
20.	Randomized Controlled Trial/
21.	Controlled Clinical Trials as Topic/
22.	Randomized controlled trial.pt
23.	Controlled clinical trial.pt
24.	(randomi#ed or randomly or placebo or sham).ab
25.	trial.ti
26.	((clinical or controlled or comparative or placebo or sham or prospective* or randomi#ed) adj3 (trial or study)).tw
27.	(random* adj7 (allocat* or allot* or assign* or basis* or divid* or order*)).tw
28.	((singl* or doubl* or trebl* or tripl*) adj7 (blind* or mask*)).tw
29.	(cross?over* or (cross adj1 over*)).tw
30.	((allocat* or allot* or assign* or divid*) adj3 (condition* or experiment* or intervention* or treatment* or therap* or control* or group*)).tw
31.	Or/19-30
32.	9 and 18 and 31
33.	limit 32 to human

CINAHL for Questions 2 and 3 (via EBSCO)

1.	MM “Patella”
2.	MM “Patellofemoral Pain Syndrome”
3.	MM “Chondromalacia Patella”
4.	(anterior knee pain)
5.	((patell* OR femoropatell* OR femoro-patell* OR retropatell*) N2 (pain OR

	syndrome OR dysfunction))
6.	((chondromalac* OR chondropath* OR chondrosis) N2 (knee* OR patell* OR femoropatell* OR femoro-patell* OR retropatell*))
7.	((lateral compression OR lateral facet OR lateral pressure OR odd facet) N2 (syndrome OR pain))
8.	or/1-7
9.	MM “Balance, Postural”
10.	MM “Proprioception”
11.	MM “Rehabilitation”
12.	(balanc* OR equilibrium OR postur*)
13.	((cent*) N2 (pressure))
14.	((postur* OR $motor OR neuromuscular) N1 (sway OR stabili* OR instabili* OR control* OR oscillat* OR perturbation))
15.	((single OR one) N2 (balance OR stance OR stabili*))
16.	(exercis* OR train* OR program* OR protocol OR physiotherapy OR physical therap*)
17.	or/9-16
18.	MM “Clinical Trials”
19.	MM “Randomized Controlled Trials”
20.	PT “Randomized controlled trial”
21.	AB randomi?ed
22.	AB placebo
23.	AB sham
24.	AB randomly
25.	TI trial
26.	((clinical or controlled or comparative or placebo or prospective* or randomi?ed) N3 (trial or study))
27.	(random* N7 (allocat* or allot* or assign* or basis* or divid* or order*))
28.	((singl* or doubl* or trebl* or tripl*) N7 (blind* or mask*))
29.	(cross?over* or (cross N1 over*))
30.	((allocat* or allot* or assign* or divid*) N3 (condition* or experiment* or intervention* or treatment* or therap* or control* or group*))
31.	Or/18-30
32.	8 and 17 and 31
33.	limit 32 to human

SPORTDiscus for Questions 2 and 3 (via EBSCO)

1.	DE “PATELLOFEMORAL joint”
2.	DE “PATELLA”
3.	DE “PLICA syndrome”
4.	DE “PATELLOFEMORAL joint injuries”
5.	DE “CHONDROMALACIA patellae”
6.	(anterior knee pain)
7.	((patell* OR femoropatell* OR femoro-patell* OR retropatell*) N2 (pain OR syndrome OR dysfunction))
8.	((chondromalac* OR chondropath* OR chondrosis) N2 (knee* OR patell* OR femoropatell* OR femoro-patell* OR retropatell*))
9.	((lateral compression OR lateral facet OR lateral pressure OR odd facet) N2

	(syndrome OR pain))
10.	OR/1-9
11.	DE “POSTURE disorders”
12.	DE “BALANCE disorders”
13.	DE “PROPRIOCEPTION”
14.	DE “REHABILITATION”
15.	(balanc* OR equilibrium OR postur*)
16.	((cent*) N2 (pressure))
17.	((postur* OR $motor OR neuromuscular) N1 (sway OR stabili* OR instabili* OR control* OR oscillat* OR perturbation))
18.	((single OR one) N2 (balance OR stance OR stabili*))
19.	(exercis* OR train* OR program* OR protocol OR physiotherapy OR physical therap*)
20.	or/11-19
21.	AB Clinical
22.	AB randomi?ed
23.	AB placebo
24.	AB sham
25.	AB randomly
26.	TI trial
27.	TX ((clinical or controlled or comparative or placebo or prospective* or randomi?ed) N3 (trial or study))
28.	TX (random* N7 (allocat* or allot* or assign* or basis* or divid* or order*))
29.	TX ((singl* or doubl* or trebl* or tripl*) N7 (blind* or mask*))
30.	TX (cross?over* or (cross N1 over*))
31.	TX ((allocat* or allot* or assign* or divid*) N3 (condition* or experiment* or intervention* or treatment* or therap* or control* or group*))
32.	Or/21-31
33.	10 and 20 and 32

Web of Science for Questions 2 and 3

1.	TS=(patellofemoral OR patella)
2.	TS=(anterior knee pain)
3.	TS=((patell* OR femoropatell* OR femoro-patell* OR retropatell*) NEAR/2 (pain OR syndrome OR dysfunction))
4.	TS=((chondromalac* OR chondropath* OR chondrosis) NEAR/2 (knee * OR patell* OR femoropatell* OR femoro-patell* OR retropatell*))
5.	TS=((compression OR facet OR pressure OR odd) NEAR/2 (syndrome OR pain))
6.	or/1-5
7.	TS=(balanc* OR equilibrium OR postur* OR proprioception)
8.	TS=((cent*) NEAR/2 (pressure))
9.	TS=((postur* OR motor OR neuromuscular) NEAR/1 (sway OR stabili* OR instabili* OR control* OR oscillat* OR perturbation))
10.	TS=((single OR one) NEAR/2 (balance OR stance OR stabili*))
11.	TS=(rehabilitation OR physical therapy OR exercise OR physiotherapy OR training OR program OR protocol)
12.	or/7-11
13.	TS=Clinical trials

14.	TS=Randomized Controlled Trial
15.	TI=(randomized OR randomised OR trial)
16.	TS=(placebo OR sham OR randomly)
17.	TS=((clinical OR controlled OR comparative OR placebo OR prospective* OR randomized OR randomised) NEAR/3 (trial OR study))
18.	TS=(random* NEAR/7 (allocat* OR allot* or assign* OR basis* OR divid* OR order*))
19.	TS=((singl* OR doubl* OR trebl* OR tripl*) NEAR/7 (blind* OR mask*))
20.	TS=(cross?over* OR (cross NEAR/1 over*))
21.	TS=((allocat* OR allot* OR assign* OR divid*) NEAR/3 (condition* OR experiment* OR intervention* OR treatment* OR therap* OR control* OR group*))
22.	or/13-21
23.	#6 and #12 and #22
24.	TS=Animal
25.	#23 not #24

Embase for Questions 2 and 3 (via Elsevier)

1.	‘Patellofemoral Joint’:de
2.	‘Patella’:de
3.	‘Patellofemoral Pain Syndrome’:de
4.	‘Patella chondromalacia’:de
5.	‘anterior knee pain’:ab,ti
6.	((patell* OR femoropatell* OR femoro-patell* OR retropatell*) NEAR/2 (pain OR syndrome OR dysfunction)):ab,ti
7.	((chondromalac* OR chondropath* OR chondrosis) NEAR/2 (knee* OR patell* OR femoropatell* OR femoro-patell* OR retropatell*)):ab,ti
8.	((‘lateral compression’ OR ‘lateral facet’ OR ‘lateral pressure’ OR ‘odd facet’) NEAR/2 (syndrome OR pain)):ab,ti
9.	or/1-8
10.	‘Body equilibrium’:de
11.	‘Proprioception’:de
12.	‘rehabilitation’:de
13.	(balanc* OR equilibrium OR postur*):ab,ti
14.	((cent*) NEAR/2 (pressure)):ab,ti
15.	((postur* OR $motor OR neuromuscular) NEAR/1 (sway OR stabili* OR instabili* OR control* OR oscillat* OR perturbation*)):ab,ti
16.	((single OR one) NEAR/2 (balance OR stance OR stabili*)):ab,ti
17.	(exercis* OR train* OR program* OR protocol OR physiotherapy OR ‘physical therap*’):ab,ti
18.	or/10-17
19.	‘clinical trial (topic)’:de
20.	‘randomized controlled trial (topic)’:de
21.	‘controlled clinical trial (topic)’:de
22.	(randomi?ed OR randomly OR placebo OR sham):ab
23.	Trial:ti
24.	((clinical OR controlled OR comparative OR placebo OR sham OR prospective* OR randomi?ed) NEAR/3 (trial OR study)):ab,ti
25.	(random* NEAR/7 (allocat* OR allot* OR assign* OR basis* OR divid* OR

	order*)):ab,ti
26.	((singl* or doubl* or trebl* or tripl*) NEAR/7 (blind* or mask*)):ab,ti
27.	(cross?over* or (cross NEAR/1 over*)):ab,ti
28.	((allocat* or allot* or assign* or divid*) NEAR/3 (condition* or experiment* or intervention* or treatment* or therap* or control* or group*)):ab,ti
29.	Or/19-28
30.	9 and 18 and 29
31.	limit 30 to human

Cochrane Library for Questions 2 and 3

1.	MeSH descriptor: [Patellofemoral Joint] this term only
2.	MeSH descriptor: [Patella] this term only
3.	MeSH descriptor: [Patellofemoral Pain Syndrome] this term only
4.	MeSH descriptor: [Chondromalacia Patellae] this term only
5.	(anterior knee pain)
6.	((patell* or femoropatell* or femoro-patell* or retropatell*) NEAR/2 (pain or syndrome or dysfunction))
7.	((chondromalac* or chondropath* or chondrosis) NEAR/2 (knee* or patell* or femoropatell* or femoro-patell* or retropatell*))
8.	((lateral compression or lateral facet or lateral pressure or odd facet) NEAR/2 (syndrome or pain))
9.	#1 OR #2 OR #3 OR #4 OR #5 OR #6 OR #7 OR #8
10.	MeSH descriptor: [Postural Balance] this term only
11.	MeSH descriptor: [Proprioception] this term only
12	MeSH descriptor: [Rehabilitation] this term only
13.	(balanc* or equilibrium or postur*)
14.	((cent*) NEAR/2 (pressure))
15.	((postur* or $motor or neuromuscular) NEAR/1 (sway or stabili* or instabili* or control* or oscillat* or perturbation*))
16.	((single or one) NEAR/2 (balance or stance or stabili*))
17.	(exercis* or train* or program* or protocol or physiotherapy or “physical therap*”)
18.	#10 OR #11 OR #12 OR #13 OR #14 OR #15 OR #16 OR #17
17.	#9 AND #18 limited to TRIALS
