# Supplementary material for: Is Postural Control Affected in People with Patellofemoral Pain and Should it be Part of Rehabilitation? A Systematic Review with Meta-analysis
Source: Sports Med Open. 2022 Dec 12;8:144. doi: 10.1186/s40798-022-00538-4 (PMC9742077; doi:10.1186/s40798-022-00538-4)
Supplement: Supplementary file 2 — Additional file 2. Excluded studies. [file 40798_2022_538_MOESM2_ESM.pdf]

Additional file 2. Excluded studies after full-text screening with reasons (n=206).

Abrahams S, Gulliford D, Korkia P, Prince J. The Influence of Leg Positioning in Exercise Programmes for Patellofemoral Joint Pain. Journal of Orthopaedic Medicine. 2003 Jan 1;25(3):107–13.
Reason for exclusion: *No balance measure or intervention*

Aghakeshizadeh F, Letafatkar A, Thomas AC. Internal and external focus show similar effect on the gait kinematics in patients with patellofemoral pain: A randomised controlled trial. Gait Posture. 2021 Feb;84:155–61.
Reason for exclusion: *No balance measure or intervention*

Ahmadi M, Yalfani A, Gandomi F, Rashid K. The Effect of Twelve Week Neurofeedback Training on Perceptual Pain Intensity, Fear of Pain, Pelvic Drop, and Dynamic Knee Valgus Index in Men with Patellofemoral Pain Syndrome: A Randomized Double-Blind Clinical Trial. Sadra Medical Journal. 2020 Mar 20;8(2):151–64.
Reason for exclusion: *non-English paper*

Albornoz-Cabello M, Barrios-Quinta CJ, Barrios-Quinta AM, Escobio-Prieto I, Cardero-Durán M de LA, Espejo-Antunez L. Effectiveness of Tele-Prescription of Therapeutic Physical Exercise in Patellofemoral Pain Syndrome during the COVID-19 Pandemic. Int J Environ Res Public Health. 2021 Jan 25;18(3):1048.
Reason for exclusion: *No balance measure or intervention*

Albornoz-Cabello M, Ibáñez-Vera AJ, Aguilar-Ferrándiz ME, Espejo-Antúnez L. Monopolar dielectric diathermy by emission of radiofrequency in Patellofemoral pain. A single-blind-randomized clinical trial. Electromagn Biol Med. 2020 Oct 1;39(4):282–9.
Reason for exclusion: *No balance measure or intervention*

Almeida GPL, Rodrigues HL das N, Coelho BAL, Rodrigues CAS, Lima PO de P. Anteromedial versus posterolateral hip musculature strengthening with dose-controlled in women with patellofemoral pain: A randomized controlled trial. Phys Ther Sport. 2021 May;49:149–56.
Reason for exclusion: *No balance measure or intervention*

Alonazi A, Hasan S, Anwer S, et al. Efficacy of Electromyographic-Biofeedback Supplementation Training with Patellar Taping on Quadriceps Strengthening in Patellofemoral Pain Syndrome among Young Adult Male Athletes. Int J Environ Res Public Health. 2021;18(9):4514.
Reason for exclusion: *No balance measure or intervention*

Alshaharani MS, Lohman EB, Bahjri K, Harp T, Alameri M, Jaber H, et al. Comparison of Protonics^{TM} Knee Brace With Sport Cord on Knee Pain and Function in Patients With Patellofemoral Pain Syndrome: A Randomized Controlled Trial. J Sport Rehabil. 2020 Jul 1;29(5):547–54.
Reason for exclusion: *No balance measure or intervention*

Araújo CGA, de Souza Guerino Macedo C, Ferreira D, Shigaki L, da Silva RA. Mcconnell's patellar taping does not alter knee and hip muscle activation differences during proprioceptive exercises: A randomized placebo-controlled trial in women with

patellofemoral pain syndrome. J Electromyogr Kinesiol. 2016 Dec;31:72–80.
Reason for exclusion: *No balance measure or intervention*

Arendt E. Hip-strengthening exercises before functional exercises reduced pain in women with patellofemoral pain syndrome. J Bone Joint Surg Am. 2012 May 16;94(10):940.
Reason for exclusion: *No balance measure or intervention*

Arhos EK, Lang CE, Steger-May K, Van Dillen LR, Yemm B, Salsich GB. Task-specific movement training improves kinematics and pain during the Y-balance test and hip muscle strength in females with patellofemoral pain. J ISAKOS. 2021 Sep;6(5):277–82.
Reason for exclusion: *Design*

Arif FSHSMA. Effects of strengthening of hip abductors and lateral rotators for improving pain & functional limitation in patients with patellofemoral dysfunction -. Rawal Medical Journal. 2020;45(1):236–9.
Reason for exclusion: *Both groups did the same interventions*

Arrebola LS, Teixeira de Carvalho R, Lam Wun PY, Rizzi de Oliveira P, Firmo Dos Santos J, Coutinho de Oliveira VG, et al. Investigation of different application techniques for Kinesio Taping® with an accompanying exercise protocol for improvement of pain and functionality in patients with patellofemoral pain syndrome: A pilot study. J Bodyw Mov Ther. 2020 Jan;24(1):47–55.
Reason for exclusion: *No balance measure or intervention*

Ashraf MJ, Ghasemi G, Fallah AR. The effect of combined training (hip abductor and external rotators strengthening + balance) on pain and performance in the patients with patellofemoral pain syndrome. 2017 Jan 1;22:82–92.
Reason for exclusion: *Non-English paper*

Asif M, Sajjad Y, Naeem AA, et al. Comparison of Specific Hip Strengthening Exercises and Conventional Knee Exercises on Pain, Muscle Strength and Function in Sprinters with Patello-Femoral Pain Syndrome. Pakistan Journal of Medical & Health Sciences. 2022;16(05):686-686.
Reason for exclusion: *No balance measure or intervention*

Atkins LT, James CR, Yang HS, et al. Immediate Improvements in Patellofemoral Pain Are Associated With Sagittal Plane Movement Training to Improve Use of Gluteus Maximus Muscle During Single Limb Landing. Phys Ther. 2021;101(10):pzab165.
Reason for exclusion: *Design*

Avraham F, Aviv S, Ya'akobi P, Faran H, Fisher Z, Goldman Y, et al. The efficacy of treatment of different intervention programs for patellofemoral pain syndrome--a single blinded randomized clinical trial. Pilot study. ScientificWorldJournal. 2007 Aug 24;7:1256–62.
Reason for exclusion: *No balance measure or intervention*

Azab AR, Abdelbasset WK, Basha MA, et al. Incorporation of Pilates-based core strengthening exercises into the rehabilitation protocol for adolescents with patellofemoral pain syndrome: a randomized clinical trial. Eur Rev Med Pharmacol

Sci. 2022;26(4):1091-1100.
Reason for exclusion: *No balance measure or intervention*

Baellow A, Glaviano NR, Hertel J, Saliba SA. Lower Extremity Biomechanics During a Drop-Vertical Jump and Muscle Strength in Women With Patellofemoral Pain. J Athl Train. 2020 Jun 23;55(6):615–22.
Reason for exclusion: *No balance measure or intervention*

Bagheri S, Hosseini SH, Saki F, Nikoo MR, Yarahmadi A. The effects of water-based posterolateral hip muscle strengthening on pain intensity and function in females with patellofemoral pain syndrome. Scientific Journal of Kurdistan University of Medical Sciences. 2017 Jan 10;21(6):99–109.
Reason for exclusion: *Non-English paper*

Bagheri S, Naderi A, Mirali S, Calmeiro L, Brewer BW. Adding Mindfulness Practice to Exercise Therapy for Female Recreational Runners With Patellofemoral Pain: A Randomized Controlled Trial. J Athl Train. 2021 Aug 1;56(8):902–11.
Reason for exclusion: *No balance measure or intervention*

Bagheri S, Shojaedin SS, Jamshidi AA, Letafatkar A, Nikoo MR. Electromyographic responses of vastus muscles to dynamic postural perturbations in athletes with patellofemoral pain syndrome. 2016 Jan 1;21:49–60.
Reason for exclusion: *Non-English paper*

Bakhtiary AH, Fatemi E. Open versus closed kinetic chain exercises for patellar chondromalacia. Br J Sports Med. 2008 Feb;42(2):99–102; discussion 102.
Reason for exclusion: *No balance measure or intervention*

Balci P, Tunay V, Baltaci G, Atay A. The effects of two different closed kinetic chain exercises on muscle strength and proprioception in patients with patellofemoral pain syndrome. Acta orthopaedica et traumatologica turcica. 2004 Jan 1;43:419–25.
Reason for exclusion: *No balance measure or intervention*

Baldon R de M, Serrão FV, Scattone Silva R, Piva SR. Effects of functional stabilization training on pain, function, and lower extremity biomechanics in women with patellofemoral pain: a randomized clinical trial. J Orthop Sports Phys Ther. 2014 Apr;44(4):240–51, A1–8.
Reason for exclusion: *Both groups did the same interventions*

Banan SM, Oliyaie GR, Mir M, Talebi GA. The Effectiveness of Exercise Therapy Based on Sahrmann Approach in Patients with Patella-Femoral Pain Syndrome. Journal of Babol University of Medical Sciences. 2016 Aug 10;18(8):7–13.
Reason for exclusion: *No balance measure or intervention*

Basbug P, Kilic RT, Atay AO, Bayrakcı Tunay V. The effects of progressive neuromuscular exercise program and taping on muscle strength and pain in patellofemoral pain. A randomized controlled blind study. Somatosens Mot Res. 2022;39(1):39-45.
Reason for exclusion: *No balance measure or intervention*

Begum R, Tassadaq N, Ahmad S, Qazi WA, Javed S, Murad S. Effects of McConnell taping

combined with strengthening exercises of vastus medialis oblique in females with patellofemoral pain syndrome. J Pak Med Assoc. 2020 Apr;70(4):728–30.
Reason for exclusion: *No balance measure or intervention*

Bily W, Trimmel L, Mödlin M, Kaider A, Kern H. Training program and additional electric muscle stimulation for patellofemoral pain syndrome: a pilot study. Arch Phys Med Rehabil. 2008 Jul;89(7):1230–6.
Reason for exclusion: *Both groups did the same interventions*

Bolgla LA, Earl-Boehm J, Emery C, Hamstra-Wright K, Ferber R. Pain, function, and strength outcomes for males and females with patellofemoral pain who participate in either a hip/core- or knee-based rehabilitation program. Int J Sports Phys Ther. 2016 Dec;11(6):926–35.
Reason for exclusion: *Design*

Bolgla LA, Malone TR, Umberger BR, Uhl TL. Comparison of hip and knee strength and neuromuscular activity in subjects with and without patellofemoral pain syndrome. Int J Sports Phys Ther. 2011 Dec;6(4):285–96.
Reason for exclusion: *No balance measure or intervention*

Boling MC, Bolgla LA, Mattacola CG, Uhl TL, Hosey RG. Outcomes of a weight-bearing rehabilitation program for patients diagnosed with patellofemoral pain syndrome. Arch Phys Med Rehabil. 2006 Nov;87(11):1428–35.
Reason for exclusion: *Design*

Botta AFB, Waiteman MC, Perez VO, et al. Trunk muscle endurance in individuals with and without patellofemoral pain: Sex differences and correlations with performance tests. *Phys Ther Sport*. 2021;52:248-255.
Reason for exclusion: *No balance measure or intervention*

Celik D, Argut SK, Türker N, Kilicoglu OI. The effectiveness of superimposed neuromuscular electrical stimulation combined with strengthening exercises on patellofemoral pain: A randomized controlled pilot trial. J Back Musculoskelet Rehabil. 2020;33(4):693–9.
Reason for exclusion: *No balance measure or intervention*

Citaker S, Kaya D, Yuksel I, Yosmaoglu B, Nyland J, Atay OA, et al. Static Balance in Patients With Patellofemoral Pain Syndrome. Sports Health. 2011 Nov;3(6):524–7.
Reason for exclusion: *Design*

Claudon B, Poussel M, Billon-Grumillier C, Beyaert C, Paysant J. Knee kinetic pattern during gait and anterior knee pain before and after rehabilitation in patients with patellofemoral pain syndrome. Gait Posture. 2012 May;36(1):139–43.
Reason for exclusion: *No balance measure or intervention*

Collins N, Crossley K, Beller E, Darnell R, McPoil T, Vicenzino B. Foot orthoses and physiotherapy in the treatment of patellofemoral pain syndrome: randomised clinical trial. BMJ. 2008 Oct 24;337:a1735.
Reason for exclusion: *No balance measure or intervention*

Constantinou A, Mamais I, Papathanasiou G, Lamnisos D, Stasinopoulos D. Comparing hip and knee focused exercises versus hip and knee focused exercises with the use of blood flow restriction training in adults with patellofemoral pain. Eur J Phys Rehabil Med. 2022;58(2):225-235.
Reason for exclusion: *No balance measure or intervention*

Corum M, Basoglu C, Yakal S, Sahinkaya T, Aksoy C. Effects of whole body vibration training on isokinetic muscular performance, pain, function, and quality of life in female patients with patellofemoral pain: a randomized controlled trial. J Musculoskelet Neuronal Interact. 2018 Dec 1;18(4):473–84.
Reason for exclusion: *No balance measure or intervention*

Cowan SM, Bennell KL, Crossley KM, Hodges PW, McConnell J. Physical therapy alters recruitment of the vasti in patellofemoral pain syndrome. Med Sci Sports Exerc. 2002a Dec;34(12):1879–85.
Reason for exclusion: *No balance measure or intervention*

Cowan SM, Bennell KL, Hodges PW, Crossley KM, McConnell J. Simultaneous feedforward recruitment of the vasti in untrained postural tasks can be restored by physical therapy. J Orthop Res. 2003 May;21(3):553–8.
Reason for exclusion: *No balance measure or intervention*

Cowan SM, Hodges PW, Bennell KL, Crossley KM. Altered vastii recruitment when people with patellofemoral pain syndrome complete a postural task. Arch Phys Med Rehabil. 2002b Jul;83(7):989–95.
Reason for exclusion: *No balance measure or intervention*

Crossley K, Bennell K, Green S, Cowan S, McConnell J. Physical therapy for patellofemoral pain: a randomized, double-blinded, placebo-controlled trial. Am J Sports Med. 2002 Dec;30(6):857–65.
Reason for exclusion: *No balance measure or intervention*

Crossley KM, Cowan SM, McConnell J, Bennell KL. Physical therapy improves knee flexion during stair ambulation in patellofemoral pain. Med Sci Sports Exerc. 2005 Feb;37(2):176–83.
Reason for exclusion: *No balance measure or intervention*

Çubukçu D, Sarsan A, Topuz O, Ardic F. Efficacy of open versus closed kinetic chain exercises for patients with patellofemoral pain syndrome. Journal of Rheumatology and Medical Rehabilitation. 2004 Mar 1;15:16–25.
Reason for exclusion: *Non-English paper*

Demirci S, Bayrakci Tunay. Comparison of effect of mobilization with movement and taping on balance and function in patellofemoral pain syndrome. Fizyoterapi rehabilitasyon. 2015;26(2).
Reason for exclusion: *Design*

Denton J, Willson JD, Ballantyne BT, Davis IS. The addition of the Protonics brace system to a rehabilitation protocol to address patellofemoral joint syndrome. J Orthop Sports Phys Ther. 2005 Apr;35(4):210–9.
Reason for exclusion: *No balance measure or intervention*

Dolak KL, Silkman C, Medina McKeon J, Hosey RG, Lattermann C, Uhl TL. Hip strengthening prior to functional exercises reduces pain sooner than quadriceps strengthening in females with patellofemoral pain syndrome: a randomized clinical trial. J Orthop Sports Phys Ther. 2011 Aug;41(8):560–70.
Reason for exclusion: *Both groups did the same interventions*

Drew BT, Conaghan PG, Smith TO, Selfe J, Redmond AC. The effect of targeted treatment on people with patellofemoral pain: a pragmatic, randomised controlled feasibility study. BMC Musculoskeletal Disorders. 2017 Aug 4;18(1):338.
Reason for exclusion: *No balance measure or intervention*

Dursun N, Dursun E, Kiliç Z. Electromyographic biofeedback-controlled exercise versus conservative care for patellofemoral pain syndrome. Arch Phys Med Rehabil. 2001 Dec;82(12):1692–5.
Reason for exclusion: *Both groups did the same interventions*

Eburne J, Bannister G. The McConnell regimen versus isometric quadriceps exercises in the management of anterior knee pain. A randomised prospective controlled trial. The Knee. 1996 Aug 1;3(3):151–3.
Reason for exclusion: *No balance measure or intervention*

EL Harrison, MS Sheppard, AM McQuarrie. A randomised controlled trial of physical therapy treatment programs in patellofemoral pain syndrome. Physiother Canada. 1999;93–100.
Reason for exclusion: *No balance measure or intervention*

Eng JJ, Pierrynowski MR. Evaluation of soft foot orthotics in the treatment of patellofemoral pain syndrome. Phys Ther. 1993 Feb;73(2):62–8; discussion 68-70.
Reason for exclusion: *No balance measure or intervention*

Erdoganoglu Y, Pepe M, Kaya D, Tagrikulu B, Aksahin E, Aktekin CN. Lower extremity alignment due to patellofemoral syndrome and dynamic postural balance. J Orthop Surg (Hong Kong). 2020 Apr;28(1):2309499019900819.
Reason for exclusion: *Design*

Erel S, Özkan H. A comparison of the effects of closed and open kinetic chain exercises on functional status in patellofemoral pain syndrome. Fizyoterapi Rehabilitasyon. 2011 Dec 1;22:217–23.
Reason for exclusion: *Non-English paper*

Esculier J-F, Bouyer LJ, Dubois B, Fremont P, Moore L, McFadyen B, et al. Is combining gait retraining or an exercise programme with education better than education alone in treating runners with patellofemoral pain?A randomised clinical trial. Br J Sports Med. 2018 May;52(10):659–66.
Reason for exclusion: *No balance measure or intervention*

Esculier J-F, Bouyer LJ, Roy J-S. The Effects of a Multimodal Rehabilitation Program on Symptoms and Ground-Reaction Forces in Runners With Patellofemoral Pain Syndrome. J Sport Rehabil. 2016 Feb;25(1):23–30.
Reason for exclusion: *Design*

Eslamian F, Aminabad F, Toopchizadeh V, Kharrazi B. The Associations of Vitamin D Deficiency with Knee Pain and Biomechanical Abnormalities in Young Iranian Patients with Patellofemoral Pain Syndrome: A Case-Control Study. Iranian Red Crescent Medical Journal. 2018 Aug 8;In Press.
Reason for exclusion: *No balance measure or intervention*

Espí-López GV, Serra-Añó P, Vicent-Ferrando J, Sánchez-Moreno-Giner M, Arias-Buría JL, Cleland J, et al. Effectiveness of Inclusion of Dry Needling in a Multimodal Therapy Program for Patellofemoral Pain: A Randomized Parallel-Group Trial. J Orthop Sports Phys Ther. 2017 Jun;47(6):392–401.
Reason for exclusion: *No balance measure or intervention*

Etemadi M, Asadi Z, Hedayati R, Salavati M, Aminianfar A. Effects of the surface instability degrees on dynamic postural stability in anterior knee pain patients and healthy subjects. Koomesh. 2013 Jan 1;15:67–77.
Reason for exclusion: *Non-English paper*

Evcik D. Home-Based Exercise and Patellar Bracig in the Treatment of Patellofemoral Pain Syndrome. Türkiye Fiziksel Tıp ve Rehabilitasyon Dergisi. 2010 Oct 4;56:100–4.
Reason for exclusion: *No balance measure or intervention*

Fukuda TY, Rossetto FM, Magalhães E, Bryk FF, Lucareli PRG, de Almeida Aparecida Carvalho N. Short-term effects of hip abductors and lateral rotators strengthening in females with patellofemoral pain syndrome: a randomized controlled clinical trial. J Orthop Sports Phys Ther. 2010 Nov;40(11):736–42.
Reason for exclusion: *No balance measure or intervention*

Gavish L, Spitzer E, Friedman I, Lowe J, Folk N, Zarbiv Y, et al. Photobio modulation in addition to physiotherapy for overuse anterior knee pain in combat soldiers: a double-blind, randomized, sham-controlled trial. Lasers in Medical Science. 2020;35(1):284.
Reason for exclusion: *Design*

Gerrard B. The Patello-Femoral Pain Syndrome: A Clinical Trial of the McConnell Programme. Australian Journal of Physiotherapy. 1989 Jan 1;35(2):71–80.
Reason for exclusion: *No balance measure or intervention*

Ghourbanpour A, Talebi GA, Hosseinzadeh S, Janmohammadi N, Taghipour M. Effects of patellar taping on knee pain, functional disability, and patellar alignments in patients with patellofemoral pain syndrome: A randomized clinical trial. J Bodyw Mov Ther. 2018 Apr;22(2):493–7.
Reason for exclusion: *No balance measure or intervention*

Glaviano NR, Marshall AN, Mangum LC, Hart JM, Hertel J, Russell S, et al. Impairment-Based Rehabilitation With Patterned Electrical Neuromuscular Stimulation and Lower Extremity Function in Individuals With Patellofemoral Pain: A Preliminary Study. J

Athl Train. 2019 Mar;54(3):255–69.
Reason for exclusion: *Both groups did the same interventions*

Golpayegani M, Emami S. The effect of proprioceptive neuromuscular facilities (PNF) stretching exercise on patellofemoral pain syndrome (PFPS). Scientific Journal of Kurdistan University of Medical Sciences. 2017 Apr 10;22(1):36–42.
Reason for exclusion: *Non-English paper*

Greaves H, Comfort P, Liu A, Lee Herrington, Richard Jones. How effective is an evidence-based exercise intervention in individuals with patellofemoral pain? Physical Therapy in Sport. 2021 Sep 1;51:92–101.
Reason for exclusion: *Design*

Günay E, Sarıkaya S, Özdolap Ş, Büyükuysal Ç. Effectiveness of the kinesiotaping in the patellofemoral pain syndrome. Turk J Phys Med Rehabil. 2017 Nov 27;63(4):299–306.
Reason for exclusion: *No balance measure or intervention*

Güney H, Yüksel İ, Kaya D, Doral MN. Effects of Different Isokinetic Training Programs on Hamstring/Quadriceps Ratio and Proprioception in Patients with Patellofemoral Pain. Orthop J Sports Med. 2014 Dec 1;2(3 Suppl):2325967114S00147.
Reason for exclusion: *Design*

Hafez A, Zakaria A, Buragadda S. Eccentric versus Concentric Contraction of Quadriceps Muscles in Treatment of Chondromalacia Patellae. World Journal of Medical Sciences. 2012 Jan 1;7:197–203, 2012.
Reason for exclusion: *No balance measure or intervention*

Halabchi F, Mazaheri R, Mansournia MA, Hamedi Z. Additional Effects of an Individualized Risk Factor-Based Approach on Pain and the Function of Patients With Patellofemoral Pain Syndrome: A Randomized Controlled Trial. Clin J Sport Med. 2015 Nov;25(6):478–86.
Reason for exclusion: *No balance measure or intervention*

Hamstra-Wright KL, Aydemir B, Earl-Boehm J, Bolgla L, Emery C, Ferber R. Lasting Improvement of Patient-Reported Outcomes 6 Months After Patellofemoral Pain Rehabilitation. J Sport Rehabil. 2017a Jul;26(4):223–33.
Reason for exclusion: *Design*

Hamstra-Wright KL, Earl-Boehm J, Bolgla L, Emery C, Ferber R. Individuals With Patellofemoral Pain Have Less Hip Flexibility Than Controls Regardless of Treatment Outcome. Clin J Sport Med. 2017b Mar;27(2):97–103.
Reason for exclusion: *Design*

Hart L. Supervised exercise versus usual care for patellofemoral pain syndrome. Clin J Sport Med. 2010 Mar;20(2):133.
Reason for exclusion: *Design*

Heydari Armaki R, Abbasnia K, Motealleh A. Comparison of Trunk Flexion Proprioception Between Healthy Athletes and Athletes With Patellofemoral Pain. J Sport Rehabil.

2020;30(3):430-436.
Reason for exclusion: *No balance measure or intervention*

Holden S, Rathleff MS, Thorborg K, Holmich P, Graven-Nielsen T. Mechanistic pain profiling in young adolescents with patellofemoral pain before and after treatment: a prospective cohort study. Pain. 2020 May;161(5):1065–71.
Reason for exclusion: *No balance measure or intervention*

Hott A, Brox JI, Pripp AH, Juel NG, Liavaag S. Patellofemoral pain: One year results of a randomized trial comparing hip exercise, knee exercise, or free activity. Scand J Med Sci Sports. 2020 Apr;30(4):741–53.
Reason for exclusion: *No balance measure or intervention*

Hott A, Brox JI, Pripp AH, Juel NG, Paulsen G, Liavaag S. Effectiveness of Isolated Hip Exercise, Knee Exercise, or Free Physical Activity for Patellofemoral Pain: A Randomized Controlled Trial. Am J Sports Med. 2019 May;47(6):1312–22.
Reason for exclusion: *No balance measure or intervention*

Ismail MM, Gamaleldein MH, Hassa KA. Closed kinetic chain exercises with or without additional hip strengthening exercises in management of patellofemoral pain syndrome: a randomized controlled trial. Eur J Phys Rehabil Med. 2013 Oct;49(5):687–98.
Reason for exclusion: *No balance measure or intervention*

Jellad A, Kalai A, Guedria M, Jguirim M, Elmhamdi S, Salah S, et al. Combined Hip Abductor and External Rotator Strengthening and Hip Internal Rotator Stretching Improves Pain and Function in Patients With Patellofemoral Pain Syndrome: A Randomized Controlled Trial With Crossover Design. Orthop J Sports Med. 2021 Apr;9(4):2325967121989729.
Reason for exclusion: *No balance measure or intervention*

Karakuş D, Dulgeroglu D, Unsal S, Uçan H, Özel S. Patellofemoral Pain Syndrome: Results of a Home Exercise Program Versus Isokinetic Exercise Program on Functional Capacity. Türkiye Fiziksel Tip ve Rehabilitasyon Dergisi. 2014 Jul 6;60:63–7.
Reason for exclusion: *Non-English paper*

Karamiani F, Mostamand J, Rahimi A, Nasirian M. The Effect of Gluteus Medius Dry Needling on Pain and Physical Function of Non-athlete women with Unilateral Patellofemoral Pain Syndrome: A Double-Blind Randomized Clinical Trial. J Bodyw Mov Ther. 2022;30:23-29.
Reason for exclusion: *No balance measure or intervention*

Kaya D, Yuksel I, Callaghan M, Guney H, Atay O, Citaker S, et al. High Voltage Pulsed Galvanic Stimulation adjunct to rehabilitation program for Patellofemoral Pain Syndrome: A prospective randomized controlled trial. Turkish Journal of Physiotherapy and Rehabilitation. 2013 Apr 1;24:1–8.
Reason for exclusion: *Both groups did the same interventions*

KE Taylor, JW Brantingham. An investigation into the effect of exercise combined with patella mobilization/manipulation in the treatment of patellofemoral pain syndrome: a

randomized, assessor-blinded, controlled clinical pilot trial. European Journal of Chiropractic. 2003;5(1):5–17.
Reason for exclusion: *No balance measure or intervention*

Keays SL, Mason M, Newcombe PA. Individualized physiotherapy in the treatment of patellofemoral pain. Physiother Res Int. 2015 Mar;20(1):22–36.
Reason for exclusion: *Design*

Keays SL, Mason M, Newcombe PA. Three-Year Outcome After a 1-Month Physiotherapy Program of Local and Individualized Global Treatment for Patellofemoral Pain Followed by Self-Management. Clin J Sport Med. 2016 May;26(3):190–8.
Reason for exclusion: *No balance measure or intervention*

Kedroff L, Li Ko Lun A, Shimoni D, Bearne LM. Cognitive behavioural therapy-informed physiotherapy for patellofemoral pain: A feasibility study. Musculoskeletal Care. 2019 Dec;17(4):382–9.
Reason for exclusion: *No balance measure or intervention*

Keshmarzi SK, Gheitasi M, Miri H. The effects of six weeks of Core stabilization exercise on pain, Functional disability and Isometric strength of the trunk and lower extremities Muscle in women with patellofemoral pain syndrome. Advances in Nursing & Midwifery. 2018 Nov 3;27(1):8–16.
Reason for exclusion: *Non-English paper*

Khayambashi K, Mohammadkhani Z, Ghaznavi K, Lyle MA, Powers CM. The effects of isolated hip abductor and external rotator muscle strengthening on pain, health status, and hip strength in females with patellofemoral pain: a randomized controlled trial. J Orthop Sports Phys Ther. 2012 Jan;42(1):22–9.
Reason for exclusion: *No balance measure or intervention*

Kim HJ, Cho J, Lee S. Talonavicular joint mobilization and foot core strengthening in patellofemoral pain syndrome: a single-blind, three-armed randomized controlled trial. *BMC Musculoskelet Disord*. 2022;23(1):150.
Reason for exclusion: *No balance measure or intervention*

Kisacik P, Karaduman A, Tunay VB, Bek N, Atay ÖA. The effects of short foot exercise on pain, knee and foot biomechanics in patients with patellofemoral pain. Annals of the Rheumatic Diseases. 2018 Jun 1;77(Suppl 2):1851–2.
Reason for exclusion: *Design*

Kısacık P, Tunay VB, Bek N, Atay ÖA, Selfe J, Karaduman AA. Short foot exercises have additional effects on knee pain, foot biomechanics, and lower extremity muscle strength in patients with patellofemoral pain. J Back Musculoskelet Rehabil. 2021 May 14;
Reason for exclusion: *No balance measure or intervention*

Kölle T, Alt W, Wagner D. Effects of a 12-week home exercise therapy program on pain and neuromuscular activity in patients with patellofemoral pain syndrome. Arch Orthop Trauma Surg. 2020a Dec;140(12):1985–92.
Reason for exclusion: *Design*

Kölle T, Alt W, Wagner D. Immediate effects of an elastic patellar brace on pain, neuromuscular activity and knee kinematics in subjects with patellofemoral pain. Arch Orthop Trauma Surg. 2020b Jul;140(7):905–12.
Reason for exclusion: *No balance measure or intervention*

Korakakis V, Whiteley R. Blood flow restriction-induced pain reduction in patients with anterior knee pain. A pilot RCT. Journal of Science and Medicine in Sport. 2017 Nov 1;20:80.
Reason for exclusion: *Design*

Korakakis V, Whiteley R, Epameinontidis K. Blood flow restriction to lower body induces analgesia in patients with anterior knee pain. Manual Therapy. 2016 Sep 1;25:e140.
Reason for exclusion: *Design*

Korakakis V, Whiteley R, Epameinontidis K. Blood Flow Restriction induces hypoalgesia in recreationally active adult male anterior knee pain patients allowing therapeutic exercise loading. Phys Ther Sport. 2018 Jul;32:235–43.
Reason for exclusion: *No balance measure or intervention*

Kowall MG, Kolk G, Nuber GW, Cassisi JE, Stern SH. Patellar taping in the treatment of patellofemoral pain. A prospective randomized study. Am J Sports Med. 1996 Feb;24(1):61–6.
Reason for exclusion: *No balance measure or intervention*

Kuru T, Yalıman A, Dereli EE. Comparison of efficiency of Kinesio® taping and electrical stimulation in patients with patellofemoral pain syndrome. Acta Orthop Traumatol Turc. 2012;46(5):385–92.
Reason for exclusion: *No balance measure or intervention*

Lankhorst NE, van Middelkoop M, van Trier YDM, van Linschoten R, Koes BW, Verhaar JAN, et al. Can we predict which patients with patellofemoral pain are more likely to benefit from exercise therapy? A secondary exploratory analysis of a randomized controlled trial. J Orthop Sports Phys Ther. 2015 Mar;45(3):183–9.
Reason for exclusion: *Design*

Lee J, Lee H, Lee W. Effect of Weight-bearing Therapeutic Exercise on the Q-angle and Muscle Activity Onset Times of Elite Athletes with Patellofemoral Pain Syndrome: A Randomized Controlled Trial. J Phys Ther Sci. 2014 Jul;26(7):989–92.
Reason for exclusion: *No balance measure or intervention*

Levinger P, Gilleard W. Tibia and rearfoot motion and ground reaction forces in subjects with patellofemoral pain syndrome during walking. Gait Posture. 2007 Jan;25(1):2–8.
Reason for exclusion: *No balance measure or intervention*

Liew BXW, Abichandani D, De Nunzio AM. Individuals with patellofemoral pain syndrome have altered inter-leg force coordination. Gait & Posture. 2020 Jun 1;79:65–70.
Reason for exclusion: *No balance measure or intervention*

Lim E-H, Kim M-E, Kim S-H, Park K-N. Effects of Posterior X Taping on Movement Quality and Knee Pain Intensity during Forward-Step-Down in Patients with

Patellofemoral Pain Syndrome. J Sports Sci Med. 2020 Mar;19(1):224–30.
Reason for exclusion: *No balance measure or intervention*

Lun VMY, Wiley JP, Meeuwisse WH, Yanagawa TL. Effectiveness of patellar bracing for treatment of patellofemoral pain syndrome. Clin J Sport Med. 2005 Jul;15(4):235–40.
Reason for exclusion: *No balance measure or intervention*

Mason M, Keays SL, Newcombe PA. The effect of taping, quadriceps strengthening and stretching prescribed separately or combined on patellofemoral pain. Physiother Res Int. 2011 Jun;16(2):109–19.
Reason for exclusion: *No balance measure or intervention*

Matsuse H, Segal N, Rabe KG, Hu B, Shiba NN. The Effects of Electrical Stimulation of Antagonist Contraction during Walking in Comparison with Those of Sensory TENS on Pain Sensitivity in Obese Women with Knee Pain: A Randomized Controlled Trial. PM&R. 2018 Sep 1;10(9, Supplement 1):S15.
Reason for exclusion: *Design*

Matthews M, Rathleff MS, Claus A, McPoil T, Nee R, Crossley KM, et al. Does foot mobility affect the outcome in the management of patellofemoral pain with foot orthoses versus hip exercises? A randomised clinical trial. Br J Sports Med. 2020 Dec;54(23):1416–22.
Reason for exclusion: *No balance measure or intervention*

McCrory JL, Quick NE, Shapiro R, Ballantyne BT, Davis I. The effect of a single treatment of the Protonics system on lower extremity kinematics during gait and the lateral step up exercise. Gait Posture. 2007 Apr;25(4):544–8.
Reason for exclusion: *No balance measure or intervention*

McMullen W, Roncarati A, Koval P. Static and isokinetic treatments of chondromalacia patella: a comparative investigation. J Orthop Sports Phys Ther. 1990;12(6):256–66.
Reason for exclusion: *No balance measure or intervention*

Mickevičius M, Degens H, Kesminas R, Rutkauskas S, Satkunskienė D, Vadopalas K, et al. Early anterior knee pain in male adolescent basketball players is related to body height and abnormal knee morphology. Phys Ther Sport. 2018 Jul;32:273–81.
Reason for exclusion: *No balance measure or intervention*

Mobarra T, Hojjat DS, Rahimi DA. Comparison of knee functional ability and static and dynamic postural control between female handball players with patellofemoral pain and healthy female handball players. Scientific Journal of Kurdistan University of Medical Sciences. 21(4):94–106.
Reason for exclusion: *Non-English paper*

Mohan .G. Comparative Analysis of Swiss Ball and Foam Roller usage with SLR Technique for Hip Muscle Strengthening of Female Athletes with Patellofemoral Pain Syndrome. Biomedicine. 2018 May 14;38:219–24.
Reason for exclusion: *No balance measure or intervention*

Monika R, Yadav J, Kalra S. The effect of adding specific hip strengthening exercises to

conventional knee exercises in patients with patello femoral pain syndrome. International Journal of Physiotherapy. 2016 Feb 7;15–21.
Reason for exclusion: *No balance measure or intervention*

Mostafa MS, Khalil K, Shahram LN, Hadi M. The effects of kinesiotape and strength training on knee pain and quadriceps strength in people with patellofemoral pain syndrome (PFPS). 32011/01/01;29(159):1416–27.
Reason for exclusion: *Non-English paper*

Mostamand J, Shafizadegan Z, Tarrahi MJ, Hosseini ZS. Comparing the Effect of Kinesiology Taping on Dynamic Balance and Pain of Men and Women With Unilateral Patellofemoral Pain Syndrome. Archives of Rehabilitation. 2020 Dec 10;21(4):470–87.
Reason for exclusion: *Design*

Moyano FR, Valenza MC, Martin LM, Caballero YC, Gonzalez-Jimenez E, Demet GV. Effectiveness of different exercises and stretching physiotherapy on pain and movement in patellofemoral pain syndrome: a randomized controlled trial. Clin Rehabil. 2013 May;27(5):409–17.
Reason for exclusion: *No balance measure or intervention*

Moyne-Bressand S, Dhieux C, Decherchi P, Dousset E. Effectiveness of Foot Biomechanical Orthoses to Relieve Patients' Knee Pain: Changes in Neural Strategy After 9 Weeks of Treatment. J Foot Ankle Surg. 2017 Dec;56(6):1194–204.
Reason for exclusion: *Unspecific data*

Mustafa M, Fatima I, Tariq A, Fazal MI, Jamal MN, Sarfraz AH. Comparison between the Effect of Closed Kinetic Chain and Open Kinetic Chain exercises in the strengthening of Vastus Medialis Obliqus in subjects with Patello-Femoral Pain Syndrome - a randomized control trial. Pakistan Journal of Medical & Health Sciences. 2022;16(06):185-185
Reason for exclusion: *No balance measure or intervention*

Naidu SK. Effect of Weight and Non-Weight Bearing Quadriceps Exercises Versus Weight-Bearing Quadriceps Exercises on Patellofemoral Pain, Quadriceps Strength and Functional Ability: A Randomized Clinical Trial. 2018;5.
Reason for exclusion: *No balance measure or intervention*

Nakagawa TH, Muniz TB, Baldon R de M, Dias Maciel C, de Menezes Reiff RB, Serrão FV. The effect of additional strengthening of hip abductor and lateral rotator muscles in patellofemoral pain syndrome: a randomized controlled pilot study. Clin Rehabil. 2008 Dec;22(12):1051–60.
Reason for exclusion: *No balance measure or intervention*

Nakagawa TH, dos Santos AF, Lessi GC, Petersen RS, Scattone Silva R. Y-Balance Test Asymmetry and Frontal Plane Knee Projection Angle During Single-leg squat as Predictors of Patellofemoral Pain in Male Military Recruits. Physical Therapy in Sport. 2020 Jul 1;44:121–7.
Reason for exclusion: *Design*

Ng ECT, Chui MPY, Siu AYK, Yam VWN, Ng GYF. Ankle positioning and knee perturbation affect temporal recruitment of the vasti muscles in people with patellofemoral pain. Physiotherapy. 2011 Mar;97(1):65–70.
Reason for exclusion: *No balance measure or intervention*

Ng GYF, Wong PYK. Patellar taping affects vastus medialis obliquus activation in subjects with patellofemoral pain before and after quadriceps muscle fatigue. Clin Rehabil. 2009 Aug;23(8):705–13.
Reason for exclusion: *No balance measure or intervention*

Ng GYF, Zhang AQ, Li CK. Biofeedback exercise improved the EMG activity ratio of the medial and lateral vasti muscles in subjects with patellofemoral pain syndrome. J Electromyogr Kinesiol. 2008 Feb;18(1):128–33.
Reason for exclusion: *Outcomes*

de Oliveira Mianutti G, Bomtempo KK, Fachin BP, Pesenti FB, de Souza Guerino Macedo C. Analysis of postural control and core muscle endurance in female runners with anterior knee pain. Physical Therapy in Sport. 2021 Jan 1;47:e1–2.
Reason for exclusion: *Design*

Örsçelik A, Akpancar S, Seven MM, Erdem Y, Koca K. The Efficacy of Platelet Rich Plasma and Prolotherapy in Chondromalacia Patella Treatment. Spor Hekimliği Dergisi. 2020;55(1):028–37.
Reason for exclusion: *No balance measure or intervention*

Örsçelik A, Yildiz Y. Comparison of Single and Triple Platelet Rich Plasma Injections in the Treatment of Patellofemoral Pain Syndrome. Turkiye Klinikleri J Med Sci. 2015;35(2):78–87.
Reason for exclusion: *Intervention type*

Østerås B, Østerås H, Torstensen TA, Torsensen TA. Long-term effects of medical exercise therapy in patients with patellofemoral pain syndrome: results from a single-blinded randomized controlled trial with 12 months follow-up. Physiotherapy. 2013a Dec;99(4):311–6.
Reason for exclusion: *No balance measure or intervention*

Østerås B, Østerås H, Torstensen TA, Vasseljen O. Dose-response effects of medical exercise therapy in patients with patellofemoral pain syndrome: a randomised controlled clinical trial. Physiotherapy. 2013b Jun;99(2):126–31.
Reason for exclusion: *No balance measure or intervention*

Paoloni M, Fratocchi G, Mangone M, Murgia M, Santilli V, Cacchio A. Long-term efficacy of a short period of taping followed by an exercise program in a cohort of patients with patellofemoral pain syndrome. Clin Rheumatol. 2012 Mar;31(3):535–9.
Reason for exclusion: *No balance measure or intervention*

Partovi G, Ghaffari S, Mohammadpoor R ali, et al. Effect of Taping and Quadriceps Strengthening and Hamstring Stretching on Patello-Femoral Pain Syndrome: A Randomized Clinical Trial. Journal of Mazandaran University of Medical Sciences. 2021;31(197):55-64.
Reason for exclusion: *Non-English paper*

Pattyn E, Mahieu N, Selfe J, Verdonk P, Steyaert A, Witvrouw E. What predicts functional outcome after treatment for patellofemoral pain? Med Sci Sports Exerc. 2012 Oct;44(10):1827–33.
Reason for exclusion: *Design*

Persson E, Zetaruk M. The use of wii fit as a unique treatment option for patellofemoral syndrome. Clin J Sport Med. 2011;21(4):382–3.
Reason for exclusion: *Design*

Petersen W, Ellermann A, Rembitzki IV, Scheffler S, Herbort M, Brüggemann GP, et al. Evaluating the potential synergistic benefit of a realignment brace on patients receiving exercise therapy for patellofemoral pain syndrome: a randomized clinical trial. Arch Orthop Trauma Surg. 2016 Jul;136(7):975–82.
Reason for exclusion: *No balance measure or intervention*

Petersen W, Ellermann A, Rembitzki IV, Scheffler S, Herbort M, Sprenker FS, et al. The Patella Pro study - effect of a knee brace on patellofemoral pain syndrome: design of a randomized clinical trial. BMC Musculoskelet Disord. 2014 Jun 10;15:200.
Reason for exclusion: *Design*

Pocai BL, Provensi É, Serighelli F, et al. Effect of photobiomodulation in the patellofemoral pain syndrome; randomized clinical trial in young women. J Bodyw Mov Ther. 2021;26:263-267.
Reason for exclusion: *No balance measure or intervention*

Primana HR, Primadhi A, Ramdan A, Utoyo GA. Knee bracing vs Taping as an Adjunct to Rehabilitative Exercise in Patellofemoral Pain Syndrome Management among Basketball Players: A Prospective Study. Sport Mont. 19(3):3-7.
Reason for exclusion: *No balance measure or intervention*

Prohorova ES, Ponomareva AG, Ar'kov VV. Correction of the lumbosacral orthosis in the patients presenting with patellofemoral pain syndrome with the use of physical exercise therapy. Vopr Kurortol Fizioter Lech Fiz Kult. 2019;96(2):33–8.
Reason for exclusion: *Non-English paper*

Qi Z, Ng GYF. EMG Analysis of Vastus Medialis Obliquus/ Vastus Lateralis Activities in Subjects with Patellofemoral Pain Syndrome before and after a Home Exercise Program. Journal of Physical Therapy Science. 2007;19(2):131–7.
Reason for exclusion: *Both groups did the same interventions*

Qiu L, Zhang M, Zhang J, Gao L-N, Chen D, Liu J, et al. Chondromalacia patellae treated by warming needle and rehabilitation training. J Tradit Chin Med. 2009 Jun;29(2):90–4.
Reason for exclusion: *No balance measure or intervention*

Rasti E, Rojhani-Shirazi Z, Ebrahimi N, Sobhan MR. Effects of whole body vibration with exercise therapy versus exercise therapy alone on flexibility, vertical jump height, agility and pain in athletes with patellofemoral pain: a randomized clinical trial. BMC Musculoskelet Disord. 2020 Oct 26;21(1):705.
Reason for exclusion: *Both groups did the same interventions*

Rathleff MS, Graven-Nielsen T, Hölmich P, Winiarski L, Krommes K, Holden S, et al. Activity Modification and Load Management of Adolescents With Patellofemoral Pain: A Prospective Intervention Study Including 151 Adolescents. Am J Sports Med. 2019 Jun;47(7):1629–37.
Reason for exclusion: *Design*

Rathleff MS, Rathleff CR, Holden S, Thorborg K, Olesen JL. Exercise therapy, patient education, and patellar taping in the treatment of adolescents with patellofemoral pain: a prospective pilot study with 6 months follow-up. Pilot Feasibility Stud. 2018;4:73.
Reason for exclusion: *No balance measure or intervention*

Rathleff MS, Roos EM, Olesen JL, Rasmussen S. Early intervention for adolescents with patellofemoral pain syndrome- a pragmatic cluster randomised controlled trial. BMC Musculoskelet Disord. 2012 Jan 27;13:9.
Reason for exclusion: *Design*

Rathleff MS, Roos EM, Olesen JL, Rasmussen S. Exercise during school hours when added to patient education improves outcome for 2 years in adolescent patellofemoral pain: a cluster randomised trial. Br J Sports Med. 2015 Mar;49(6):406–12.
Reason for exclusion: *No balance measure or intervention*

Rathleff MS, Samani A, Olesen JL, Roos EM, Rasmussen S, Madeleine P. Effect of exercise therapy on neuromuscular activity and knee strength in female adolescents with patellofemoral pain-An ancillary analysis of a cluster randomized trial. Clin Biomech (Bristol, Avon). 2016 May;34:22–9.
Reason for exclusion: *No balance measure or intervention*

Riel H, Matthews M, Vicenzino B, Bandholm T, Thorborg K, Rathleff MS. Feedback Leads to Better Exercise Quality in Adolescents with Patellofemoral Pain. Med Sci Sports Exerc. 2018 Jan;50(1):28–35.
Reason for exclusion: *No balance measure or intervention*

Rodrigues C, Rodrigues H, Coelho B, Lima P, Almeida G. Strengthening of the hip posterolateral musculature versus hip anteromedial musculature in patients with patellofemoral pain: A randomized controlled trial. Physical Therapy in Sport. 2021 Jan 1;47:e5.
Reason for exclusion: *Design*

Roh Y, Park J. Training and Detraining Effects of a Rehabilitation Program with or without Electro-Cryotherapy in Patients with Anterior Knee Pain: A Randomized Trial. Applied Sciences. 2021 Jan;11(11):4812.
Reason for exclusion: *Both groups did the same interventions*

Rojhani Shirazi Z, Biabani Moghaddam M, Motealleh A. Comparative evaluation of core muscle recruitment pattern in response to sudden external perturbations in patients with patellofemoral pain syndrome and healthy subjects. Arch Phys Med Rehabil. 2014 Jul;95(7):1383–9.
Reason for exclusion: *No balance measure or intervention*

Roush MB, Sevier TL, Wilson JK, Jenkinson DM, Helfst RH, Gehlsen GM, et al. Anterior knee pain: a clinical comparison of rehabilitation methods. Clin J Sport Med. 2000 Jan;10(1):22–8.
Reason for exclusion: *No balance measure or intervention*

Sacco I de CN, Konno GK, Rojas GB, Arnone AC, Pássaro A de C, Marques AP, et al. Functional and EMG responses to a physical therapy treatment in patellofemoral syndrome patients. J Electromyogr Kinesiol. 2006a Apr;16(2):167–74.
Reason for exclusion: *No balance measure or intervention*

Sacco ICN, Konno GK, Rojas GB, Cabral CMN, Pássaro AC, Arnone AC, et al. EMG, functional and postural responses to a physical therapy treatment for patellofemoral syndrome. Fisioterapia e Pesquisa. 2006b;13(1):16–22.
Reason for exclusion: *Non-English paper*

Şahin M, Ayhan FF, Borman P, Atasoy H. The effect of hip and knee exercises on pain, function, and strength in patients with patellofemoral pain syndrome: a randomized controlled trial. Turk J Med Sci. 2016 Feb 17;46(2):265–77.
Reason for exclusion: *No balance measure or intervention*

Salsich GB, Brechter JH, Powers CM. Lower extremity kinetics during stair ambulation in patients with and without patellofemoral pain. Clin Biomech (Bristol, Avon). 2001 Dec;16(10):906–12.
Reason for exclusion: *No balance measure or intervention*

Scafoglieri A, Van den Broeck J, Willems S, Tamminga R, van der Hoeven H, Engelsma Y, et al. Effectiveness of local exercise therapy versus spinal manual therapy in patients with patellofemoral pain syndrome: medium term follow-up results of a randomized controlled trial. BMC Musculoskelet Disord. 2021 May 15;22(1):446.
Reason for exclusion: *No balance measure or intervention*

Schneider F, Labs K, Wagner S. Chronic patellofemoral pain syndrome: alternatives for cases of therapy resistance. Knee Surg Sports Traumatol Arthrosc. 2001 Sep;9(5):290–5.
Reason for exclusion: *No balance measure or intervention*

Selhorst M, Rice W, Degenhart T, Jackowski M, Tatman M. Evaluation of a treatment algorithm for patients with patellofemoral pain syndrome: a pilot study. Int J Sports Phys Ther. 2015 Apr;10(2):178–88.
Reason for exclusion: *Design*

Selhorst M, Rice W, Jackowski M, Degenhart T, Coffman S. A sequential cognitive and physical approach (SCOPA) for patellofemoral pain: a randomized controlled trial in adolescent patients. Clin Rehabil. 2018 Dec;32(12):1624–35.
Reason for exclusion: *No balance measure or intervention*

Senthil P, Jayaseelan V, Srinivasan R. A Study to Compare the Effectiveness of K taping with wall squats and K taping with standard exercise program in reducing pain and improving running performance in100 m female runners with patellofemoral pain syndrome. Annals of Tropical Medicine and Public Health. 2020 Jan 1;23.
Reason for exclusion: *No balance measure or intervention*

Servodio Iammarrone C, Cadossi M, Sambri A, Grosso E, Corrado B, Servodio Iammarrone F. Is there a role of pulsed electromagnetic fields in management of patellofemoral pain syndrome? Randomized controlled study at one year follow-up. Bioelectromagnetics. 2016 Feb;37(2):81–8.
Reason for exclusion: *Both groups did the same interventions*

Silva APMCCE, Leão G, Magalhães M, Marques AP. Static and dynamic postural control in individuals with chondromalacia patellae. Annals of the Rheumatic Diseases. 2013 Jun 1;72(Suppl 3):A570–A570.
Reason for exclusion: *Design*

Smith BE, Hendrick P, Bateman M, Moffatt F, Rathleff MS, Selfe J, et al. A loaded self-managed exercise programme for patellofemoral pain: a mixed methods feasibility study. BMC Musculoskelet Disord. 2019 Mar 27;20(1):129.
Reason for exclusion: *No balance measure or intervention*

Sobhani V, Mazloum V. The Comparison of Core Muscles Strength and Dynamic Balance in Patients with Patellofemoral Pain Syndrome and Healthy Individuals. Journal of Advances in Medical and Biomedical Research. 2017 Mar 10;25(108):107–19.
Reason for exclusion: *Non-English paper*

Soleimani F, Derisfard F, Negahban H, Esfandiarpour F. Effectiveness of knee exercises versus combined knee and hip exercises in treatment of patellofemoral pain: A randomized clinical trial. Koomesh. 2017 Jun 10;19(3):554–64.
Reason for exclusion: *Non-English paper*

Steinberg N, Tenenbaum S, Zeev A, Pantanowitz M, Waddington G, Dar G, et al. Generalized joint hypermobility, scoliosis, patellofemoral pain, and physical abilities in young dancers. BMC Musculoskelet Disord. 2021 Feb 9;22(1):161.
Reason for exclusion: *Unspecific data*

Stiene HA, Brosky T, Reinking MF, Nyland J, Mason MB. A comparison of closed kinetic chain and isokinetic joint isolation exercise in patients with patellofemoral dysfunction. J Orthop Sports Phys Ther. 1996 Sep;24(3):136–41.
Reason for exclusion: *No balance measure or intervention*

Strojnik V, Vengust R, Pavlovcic V. The effect of proprioceptive training on neuromuscular function in patients with patellar pain. Cell Mol Biol Lett. 2002;7(1):170–1.
Reason for exclusion: *Design*

Subasi V. Effectiveness of Platelet-Rich Plasma Treatment in Chondromalacia Patellae. JAREM. 2017 May 30;7(1):36–8.
Reason for exclusion: *Design*

Sutlive TG, Mitchell SD, Maxfield SN, McLean CL, Neumann JC, Swiecki CR, et al. Identification of Individuals With Patellofemoral Pain Whose Symptoms Improved After a Combined Program of Foot Orthosis Use and Modified Activity: A Preliminary Investigation. Physical Therapy. 2004 Jan 1;84(1):49–61.
Reason for exclusion: *No balance measure or intervention*

Swanson KJ. A clinical and biomechanical profile of female athletes with and without patellofemoral pain. University of Minnesota 2009.
Reason for exclusion: *Design*

Syme G, Rowe P, Martin D, Daly G. Disability in patients with chronic patellofemoral pain syndrome: a randomised controlled trial of VMO selective training versus general quadriceps strengthening. Man Ther. 2009 Jun;14(3):252–63.
Reason for exclusion: *No balance measure or intervention*

Talbot LA, Solomon Z, Webb L, Morrell C, Metter EJ. Electrical Stimulation Therapies for Active Duty Military with Patellofemoral Pain Syndrome: A Randomized Trial. Mil Med. 2020 Aug 14;185(7–8):e963–71.
Reason for exclusion: *No balance measure or intervention*

Tazesh B, Mansournia MA, Halabchi F. Additional effects of core stability exercises on pain and function of patients with patellofemoral pain: A randomized controlled trial. Journal of Orthopaedics, Trauma and Rehabilitation. Published online February 10, 2021:2210491721989075.
Reason for exclusion: *No balance measure or intervention*

Thomeé R. A comprehensive treatment approach for patellofemoral pain syndrome in young women. Phys Ther. 1997 Dec;77(12):1690–703.
Reason for exclusion: *Both groups did the same interventions*

Timm KE. Randomized controlled trial of Protonics on patellar pain, position, and function. Med Sci Sports Exerc. 1998 May;30(5):665–70.
Reason for exclusion: *No balance measure or intervention*

Tsai L-C, Lee S-J, Lin C, Yang A, Ren Y, Gaiger F, et al. Effectiveness of Off-Axis Training on Improving Knee Function in Individuals with Patellofemoral Pain. Archives of Physical Medicine and Rehabilitation. 2012 Oct 1;93(10):e13.
Reason for exclusion: *Design*

Tsai L-C, Lee SJ, Yang AJ, Ren Y, Press JM, Zhang L-Q. Effects of Off-Axis Elliptical Training on Reducing Pain and Improving Knee Function in Individuals With Patellofemoral Pain. Clin J Sport Med. 2015 Nov;25(6):487–93.
Reason for exclusion: *Design*

Tunay VB, Baltaci G, Tunay S, Ergun N. A comparison of different treatment approaches to patellofemoral pain syndrome. The Pain Clinic. 2003 Jun 1;15(2):179–84.
Reason for exclusion: *No balance measure or intervention*

Vahid M, Nader R. Comparison of the effects of vibration technique and resistive exercise on knee pain and proprioception in patients with chondromalacia patellae. Journal of Kerman University of Medical Sciences. 2014;437–45.
Reason for exclusion: *Non-English paper*

Vicenzino B, Collins N, Crossley K, Beller E, Darnell R, McPoil T. Foot orthoses and physiotherapy in the treatment of patellofemoral pain syndrome: a randomised clinical trial. BMC Musculoskelet Disord. 2008 Feb 27;9:27.
Reason for exclusion: *No balance measure or intervention*

Whittingham M, Palmer S, Macmillan F. Effects of taping on pain and function in patellofemoral pain syndrome: a randomized controlled trial. J Orthop Sports Phys Ther. 2004 Sep;34(9):504–10.
Reason for exclusion: *No balance measure or intervention*

Wiener-Ogilvie S, Jones R. A randomised trial of exercise therapy and foot orthoses as treatment for knee pain in primary care. British Journal of Podiatry. 2004;7(2):43–9.
Reason for exclusion: *No balance measure or intervention*

Willson JD, Davis IS. Lower extremity mechanics of females with and without patellofemoral pain across activities with progressively greater task demands. Clin Biomech (Bristol, Avon). 2008 Feb;23(2):203–11.
Reason for exclusion: *No balance measure or intervention*

Witvrouw E, Cambier D, Danneels L, Bellemans J, Werner S, Almqvist F, et al. The effect of exercise regimens on reflex response time of the vasti muscles in patients with anterior knee pain: a prospective randomized intervention study. Scand J Med Sci Sports. 2003 Aug;13(4):251–8.
Reason for exclusion: *No balance measure or intervention*

Witvrouw E, Danneels L, Van Tiggelen D, Willems TM, Cambier D. Open versus closed kinetic chain exercises in patellofemoral pain: a 5-year prospective randomized study. Am J Sports Med. 2004 Aug;32(5):1122–30.
Reason for exclusion: *No balance measure or intervention*

Witvrouw E, Lysens R, Bellemans J, Peers K, Vanderstraeten G. Open versus closed kinetic chain exercises for patellofemoral pain. A prospective, randomized study. Am J Sports Med. 2000 Oct;28(5):687–94.
Reason for exclusion: *No balance measure or intervention*

Wu Z, Zou Z, Zhong J, Fu X, Yu L, Wang J, et al. Effects of whole-body vibration plus hip-knee muscle strengthening training on adult patellofemoral pain syndrome: a randomized controlled trial. Disabil Rehabil. 2021 Aug 4;1–9.
Reason for exclusion: *No balance measure or intervention*

Yañez-Álvarez A, Bermúdez-Pulgarín B, Hernández-Sánchez S, Albornoz-Cabello M. Effects of exercise combined with whole body vibration in patients with patellofemoral pain syndrome: a randomised-controlled clinical trial. BMC Musculoskelet Disord. 2020 Aug 28;21(1):582.
Reason for exclusion: *No balance measure or intervention*

Yildiz Y, Aydin T, Sekir U, Cetin C, Ors F, Alp Kalyon T. Relation between isokinetic muscle strength and functional capacity in recreational athletes with chondromalacia patellae. Br J Sports Med. 2003 Dec;37(6):475–9.
Reason for exclusion: *Unspecific data*

Yip SLM, Ng GYF. Biofeedback supplementation to physiotherapy exercise programme for rehabilitation of patellofemoral pain syndrome: a randomized controlled pilot study. Clin Rehabil. 2006 Dec;20(12):1050–7.
Reason for exclusion: *Both groups did the same interventions*

Yılmaz Yelvar GD, Baltacı G, Bayrakcı Tunay V, Atay AÖ. The effect of postural stabilization exercises on pain and function in females with patellofemoral pain syndrome. Acta Orthop Traumatol Turc. 2015;49(2):166–74.
Reason for exclusion: *Both groups did the same interventions*

Yosmaoglu HB, Kaya D, Guney H, Nyland J, Baltaci G, Yuksel I, et al. Is there a relationship between tracking ability, joint position sense, and functional level in patellofemoral pain syndrome? Knee Surgery, Sports Traumatology, Arthroscopy. 2013;11(21):2564–71.
Reason for exclusion: *No balance measure or intervention*

Yosmaoğlu HB, Selfe J, Sonmezer E, Sahin İE, Duygu SÇ, Acar Ozkoslu M, et al. Targeted Treatment Protocol in Patellofemoral Pain: Does Treatment Designed According to Subgroups Improve Clinical Outcomes in Patients Unresponsive to Multimodal Treatment? Sports Health. 2020 Apr;12(2):170–80.
Reason for exclusion: *Design*

Yuksel I, Tuğay N, Erden Z, Leblebicioglu G, Doral M. Patellofemoral pain rehabilitation: Outcomes of a home based program. J Arthroplasty Arthroscopic Surg. 2001 Jan 1;12:56–60.
Reason for exclusion: *Design*

Zago J, Amatuzzi F, Rondinel T, Matheus JP. Osteopathic Manipulative Treatment Versus Exercise Program in Runners With Patellofemoral Pain Syndrome: A Randomized Controlled Trial. J Sport Rehabil. 2020 Dec 17;30(4):609–18.
Reason for exclusion: *No balance measure or intervention*

Zahednejad S, Goharpey S, Farokhnia M. Comparison of patellar taping versus patellar bracing with exercise therapy on pain and level of function in females with patellofemoral pain syndrome. Koomesh. 2017 Jun 10;19(3):677–87.
Reason for exclusion: *Non-English paper*

Zambarano E, Glaviano N, Murray A. Reliability and Utility of a Lumbopelvic-hip Complex Stability Assessment in Individuals with Patellofemoral Pain. Journal of Sports Medicine and Allied Health Sciences: Official Journal of the Ohio Athletic Trainers Association. 2022;8(1).
Reason for exclusion: *Design*

Zamboti CL, Marçal Camillo CA, Ricardo Rodrigues da Cunha AP, Ferreira TM, Macedo CSG. Impaired performance of women with patellofemoral pain during functional

tests. Braz J Phys Ther. 2021;25(2):156-161.
Reason for exclusion: *No balance measure or intervention*

Zeinalzadeh A, Nazary-Moghadam S, Sayyed Hoseinian SH, Ebrahimzadeh MH, Imani E, Karimpour S. Intra- and Inter-Session Reliability of Methods for Measuring Reaction Time in Participants with and without Patellofemoral Pain Syndrome. Arch Bone Jt Surg. 2021 Jan;9(1):102–9.
Reason for exclusion: *No balance measure or intervention*

Zemadanis K, Sykaras E, Athanasopoulos S, Mandalidis D. Mobilization-with-movement prior to exercise provides early pain and functionality improvements in patients with patellofemoral pain syndrome. International Musculoskeletal Medicine. 2015 Sep 1;37(3):101–7.
Reason for exclusion: *No balance measure or intervention*

Zhang J, Wang Y, Zhou M, Li X, Rehabilitation D, Peking HTU, et al. Clinical Effect of Low-intensity Focused Ultrasound Combined with Exercise Therapy on Chondromalacia Patellae. Journal of Capital University of Physical Education and Sports. 2020;(4):379–84.
Reason for exclusion: *Non-English paper*
