# Supplementary material for: Is Postural Control Affected in People with Patellofemoral Pain and Should it be Part of Rehabilitation? A Systematic Review with Meta-analysis
Source: Sports Med Open. 2022 Dec 12;8:144. doi: 10.1186/s40798-022-00538-4 (PMC9742077; doi:10.1186/s40798-022-00538-4)

Additional file 3A. Funnel Plot for Q1 main meta-analyses with ≥ 10 studies.

Comparison PFP x Control for AP postural stability

Original plot (Egger's test p=0.018; n=19 studies)

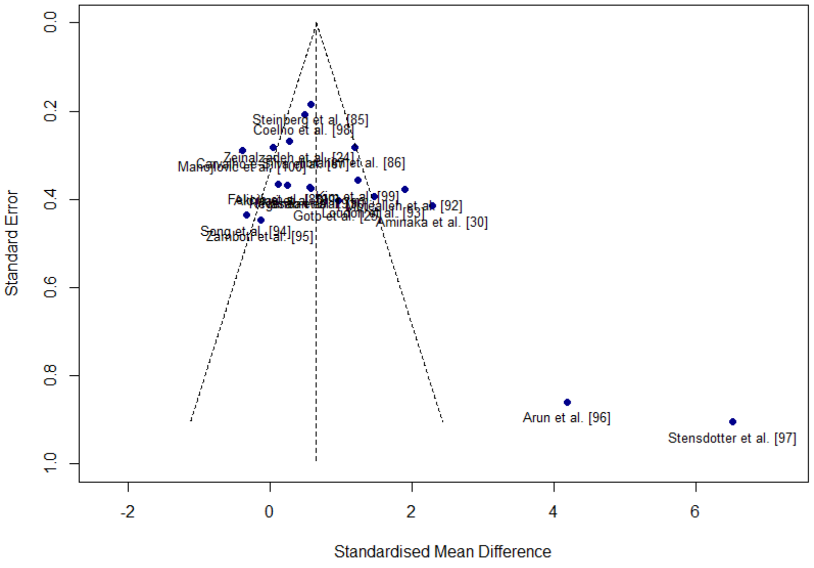

Trim and Fill analysis (Egger's test p=0.746; n=21 studies)

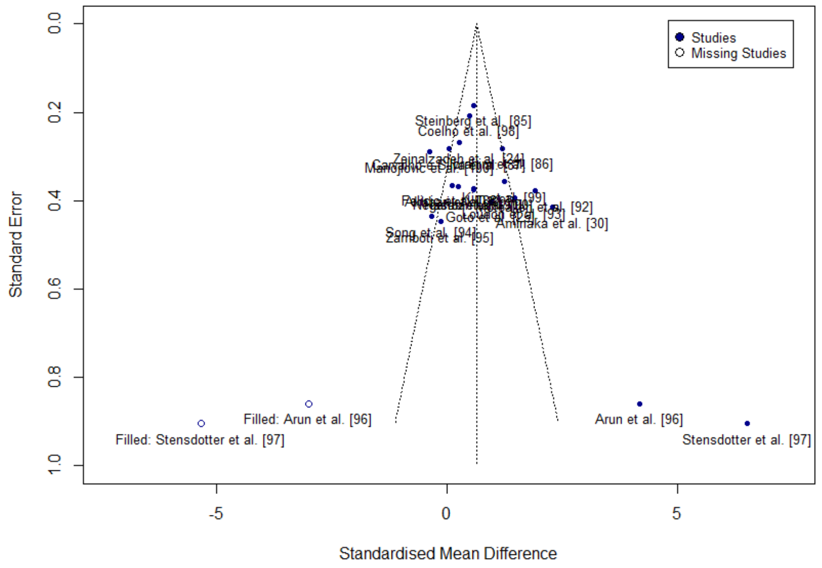

Removing outliers (Egger's test p=0.460; n=17 studies)

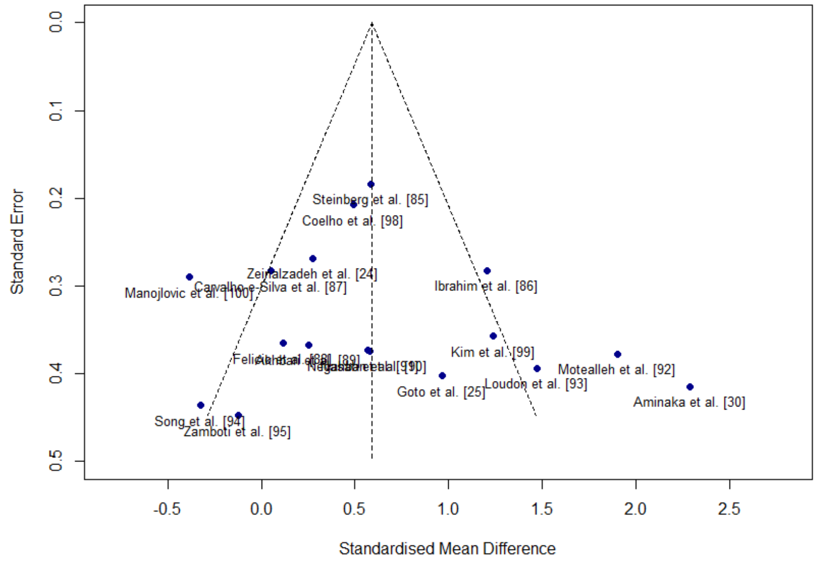

Comparison PFP x Control for ML postural stability

Original plot (Egger's test p=0.059; n=13 studies)

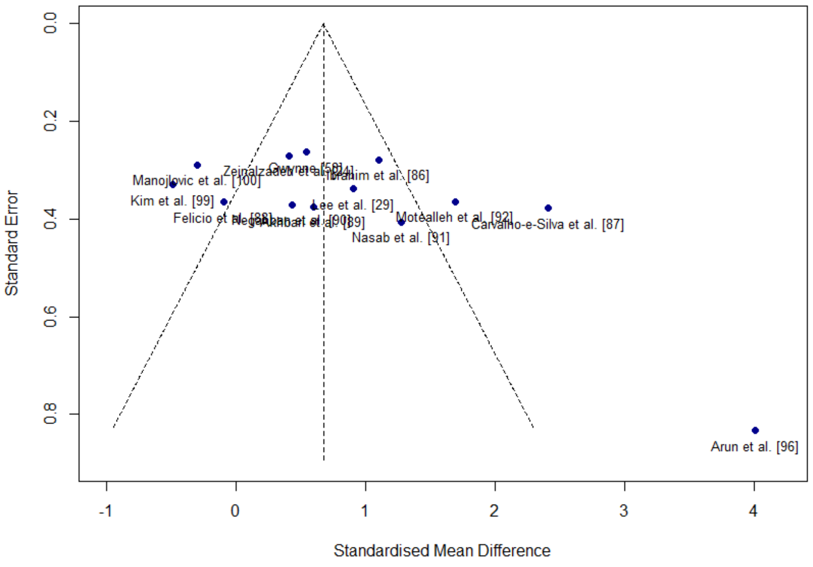

Trim and Fill analysis (Egger's test p=0.641; n=14 studies)

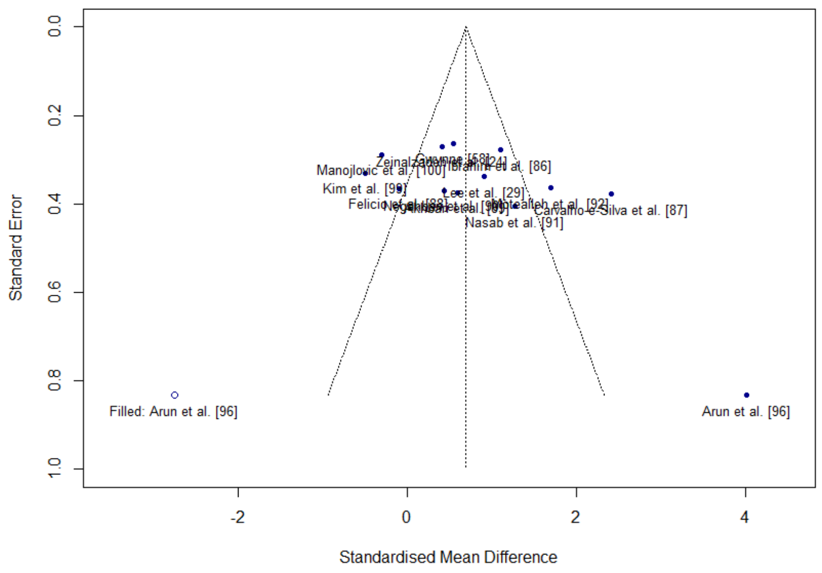

Removing outliers (Egger's test p=0.292; n=12 studies)

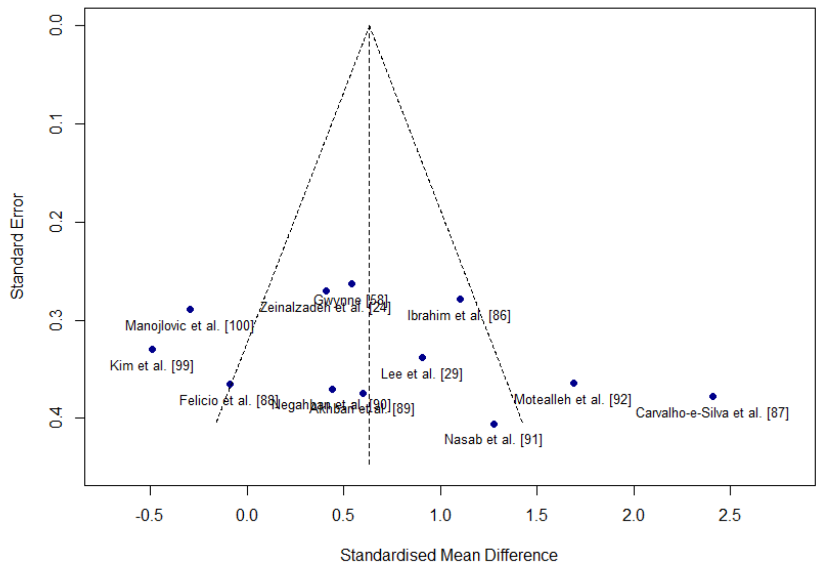

Comparison PFP x Control for Overall postural stability
Original plot (Egger's test p=0.813; n=15 studies)
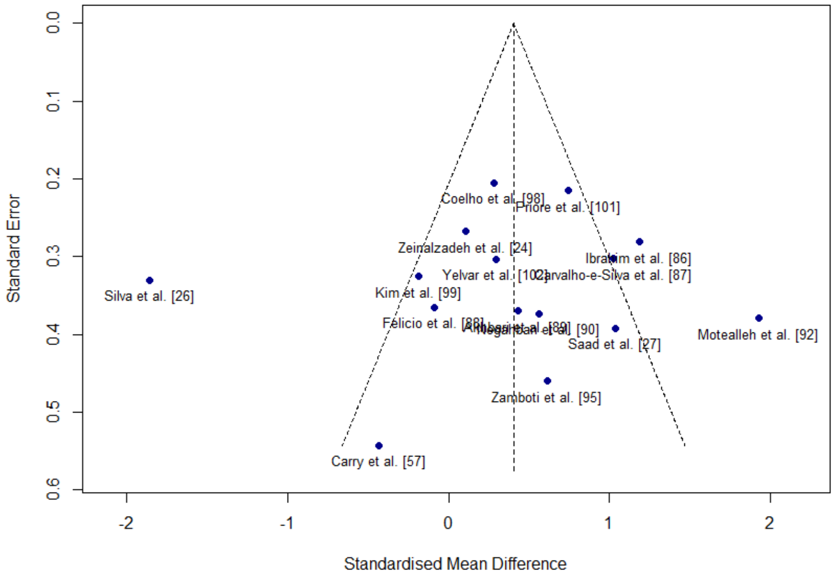

Additional file 3B. Funnel Plot for the Q2 main meta-analysis with ≥ 10 studies.

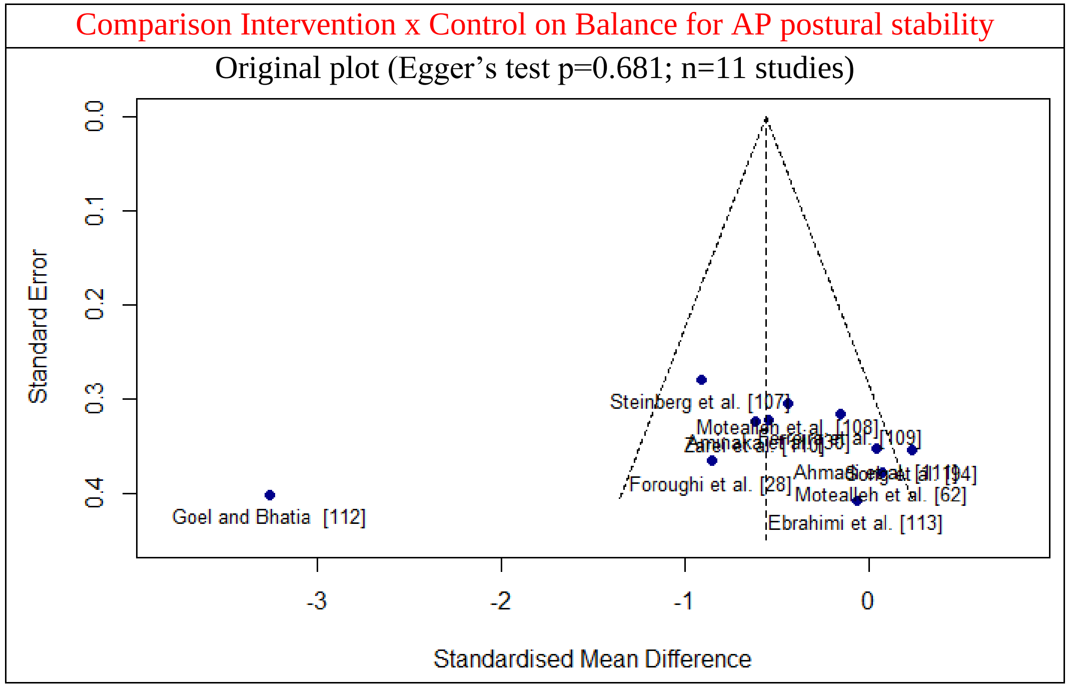

Additional file 3C. Funnel Plot for the Q3 main meta-analysis with ≥ 10 studies.

Comparison Balance x No balance interventions on Pain

Original plot (Egger's test p=0.054; n=14 studies)

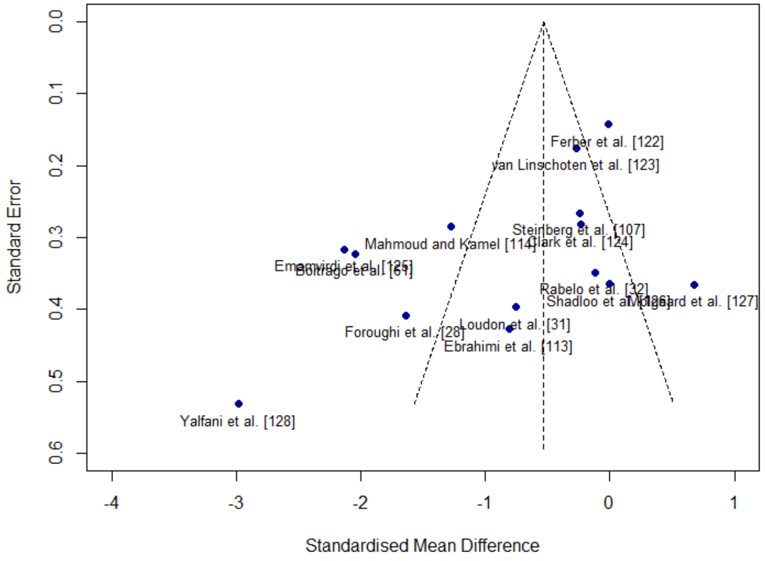

Trim and Fill analysis (Egger's test p=0.613; n=17 studies)

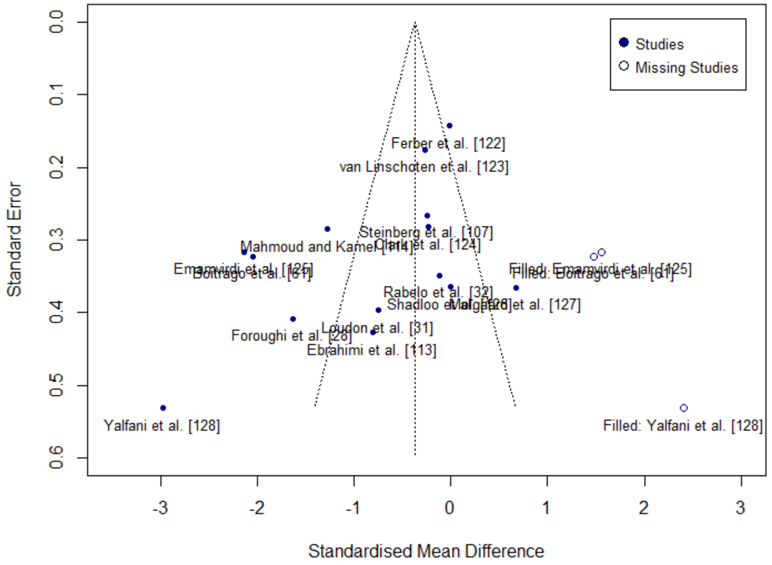

Removing outliers (Egger's test p=0.242; n=11 studies)

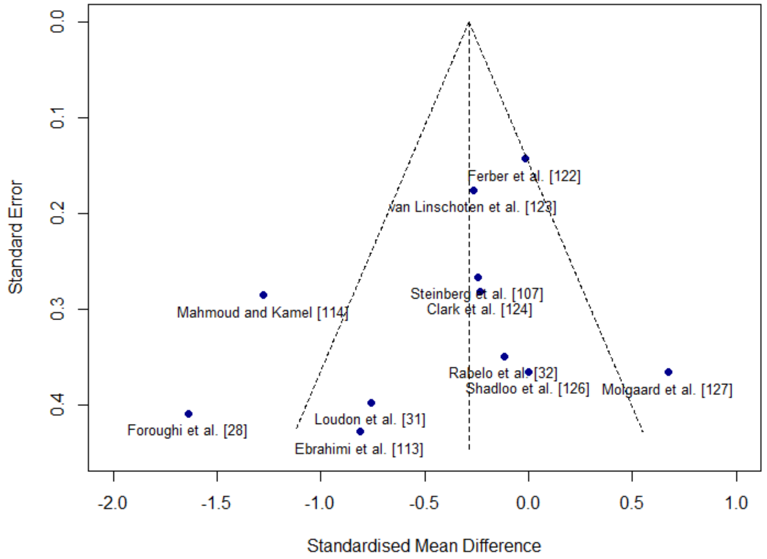

Comparison Balance x No balance interventions on Function

Original plot (Egger's test p=0.082; n=10 studies)

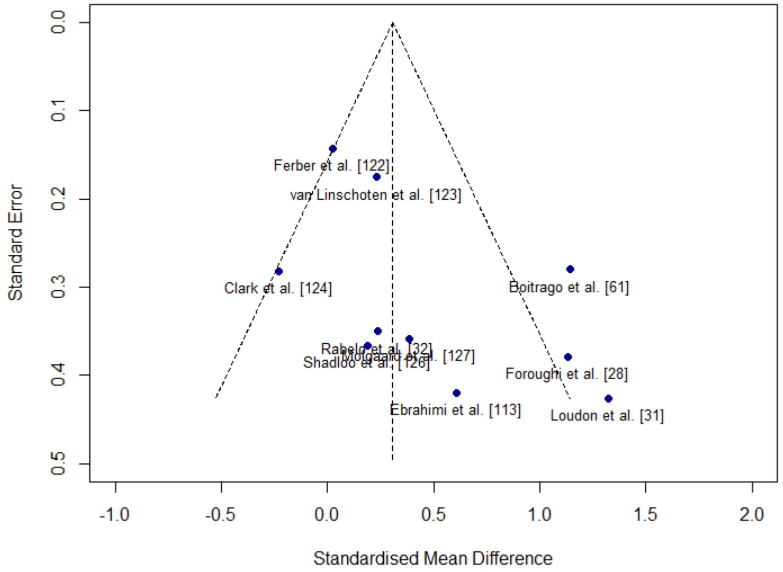

Supplement: Supplementary file 3 — Additional file 3. Funnel plots. [file 40798_2022_538_MOESM3_ESM.pdf]
