# Supplementary material for: Is Postural Control Affected in People with Patellofemoral Pain and Should it be Part of Rehabilitation? A Systematic Review with Meta-analysis
Source: Sports Med Open. 2022 Dec 12;8:144. doi: 10.1186/s40798-022-00538-4 (PMC9742077; doi:10.1186/s40798-022-00538-4)
Supplement: Supplementary file 4 — Additional file 4. GRADE description. [file 40798_2022_538_MOESM4_ESM.pdf]

Additional file 4A. Detailed GRADE description for the evidence level assessment in meta-analyses for Q1.

Analysis		Level of Evidence	Reason to downgrade the level (GRADE)
**AP postural stability; 19 studies**		**⊕⊖⊖⊖ Very Low**	Included >25% of studies with high RoB (5 studies), heterogenic outcomes, presence of publication bias
Adjustment for publication bias	Trim-and-fill; 21 studies	⊕⊕⊖⊖ Low	Included >25% of studies with high RoB (5 studies + 2 imputed), heterogenic outcomes
	Removing outliers; 17 studies	⊕⊕⊕⊖ Moderate	Heterogenic outcomes
Subgroup analyses	***by sex***		
	Females; 8 studies	⊕⊕⊕⊖ Moderate	Heterogenic outcomes
	Mixed; 10 studies	⊕⊖⊖⊖ Very Low	Included >25% of studies with high RoB (3 studies), heterogenic outcomes, presence of publication bias
	by assessment method		
	SEBT Anterior; 8 studies	⊕⊕⊕⊖ Moderate	Heterogenic outcomes
	Posturography; 4 studies	⊕⊕⊕⊖ Moderate	Included less than 100 participants per group
	CoP; 7 studies	⊕⊕⊖⊖ Low	Included >25% of studies with high RoB (2 studies), heterogenic outcomes
	by task		
	Dynamic; 14 studies	⊕⊖⊖⊖ Very Low	Included >25% of studies with high RoB (4 studies), heterogenic outcomes, presence of publication bias
	Static; 5 studies	⊕⊕⊖⊖ Low	Heterogenic outcomes, included less than 100 participants per group
**ML postural stability; 13 studies**		**⊕⊕⊕⊖ Moderate**	Heterogenic outcomes
Adjustment for publication bias	Trim-and-fill; 14 studies	⊕⊕⊕⊖ Moderate	Heterogenic outcomes
	Removing outliers; 12 studies	⊕⊕⊕⊖ Moderate	Heterogenic outcomes
Subgroup analyses	***by sex***		
	Females; 5 studies	⊕⊕⊖⊖ Low	Heterogenic outcomes, included less than 100 participants per group
	Mixed; 7 studies	⊕⊕⊖⊖ Low	Included >25% of studies with high RoB (2 studies), heterogenic outcomes
	by assessment method		
	Posturography; 4 studies	⊕⊕⊖⊖ Low	Heterogenic outcomes, included less than 100 participants per group
	CoP; 8 studies	⊕⊕⊕⊖ Moderate	Heterogenic outcomes
	by task		
	Dynamic; 9 studies	⊕⊕⊕⊖ Moderate	Heterogenic outcomes
	Static; 4 studies	⊕⊕⊖⊖ Low	Heterogenic outcomes, included less than 100 participants per group
**Overall postural stability; 15 studies**		**⊕⊕⊕⊖ Moderate**	Heterogenic outcomes
Subgroup analyses	***by sex***		
	Females; 10 studies	⊕⊕⊕⊖ Moderate	Heterogenic outcomes
	Mixed; 4 studies	⊕⊕⊕⊕ High	-
	by assessment method		
	SEBT; 2 studies	⊕⊕⊖⊖ Low	Heterogenic outcomes, included less than 100 participants per group
	Posturography; 5 studies	⊕⊕⊕⊖ Moderate	Heterogenic outcomes
	CoP; 8 studies	⊕⊕⊕⊖ Moderate	Heterogenic outcomes
	by task		
	Dynamic; 10 studies	⊕⊕⊕⊖ Moderate	Heterogenic outcomes
	Static; 5 studies	⊕⊕⊖⊖ Low	Heterogenic outcomes, included less than 100 participants per group

**SEBT Posterolateral; 4 studies**	⊕⊕⊕⊖ **Moderate**	Heterogenic outcomes
**SEBT Posteromedial; 3 studies**	⊕⊕⊕⊖ **Moderate**	Heterogenic outcomes
**AP CoP velocity; 6 studies**	⊕⊕⊕⊖ **Moderate**	Included >25% of studies with high RoB (2 studies)
**ML CoP velocity; 5 studies**	⊕⊕⊕⊖ **Moderate**	Heterogenic outcomes
**Overall CoP velocity; 4 studies**	⊕⊕⊕⊖ **Moderate**	Heterogenic outcomes

Abbreviations: AP = anteroposterior; CoP = centre of pressure; ML = mediolateral; RoB = risk of bias; SEBT = Star Excursion Balance Test.

Additional file 4B. Detailed GRADE description for the evidence level assessment in meta-analyses for Q2 (Intervention x Control).

**Analysis**		**Level of Evidence**	**Reason to downgrade the level (GRADE)**
**AP postural stability; 11 studies**		⊕⊕⊖⊖ **Low**	Included >25% of studies with high RoB (6 studies), heterogenic outcomes
Subgroup analyses	***by intervention characteristic***		
	Passive; 7 studies	⊕⊕⊖⊖ Low	Included >25% of studies with high RoB (4 studies), heterogenic outcomes
	Exercise; 4 studies	⊕⊖⊖⊖ Very Low	Included >25% of studies with high RoB (2 studies), heterogenic outcomes, included <100 participants per group
	by comparator		
	Sham/no intervention; 8 studies	⊕⊕⊖⊖ Low	Included >25% of studies with high RoB (5 studies), heterogenic outcomes
	Exercise; 3 studies	⊕⊕⊖⊖ Low	Included >25% of studies with high RoB (1 studies), included less than 100 participants per group
	by design		
	Parallel; 7 studies	⊕⊕⊕⊖ Moderate	Included >25% of studies with high RoB (3 studies)
	Crossover; 4 studies	⊕⊖⊖⊖ Very Low	Included >25% of studies with high RoB (3 studies), heterogenic outcomes, included <100 participants per group
	by study interventions		
	Balance specific; 2 studies	⊕⊖⊖⊖ Very Low	Included only studies with high RoB (-2 levels), included less than 100 participants per group
	Not specific; 9 studies	⊕⊕⊖⊖ Low	Included >25% of studies with high RoB (4 studies), heterogenic outcomes
**ML postural stability; 4 studies**		⊕⊖⊖⊖ **Very Low**	Included >25% of studies with high RoB (3 studies), heterogenic outcomes, included <100 participants per group
**Overall postural stability; 7 studies**		⊕⊕⊖⊖ **Low**	Included >25% of studies with high RoB (3 studies), heterogenic outcomes
**SEBT Posterolateral; 5 studies**		⊕⊕⊕⊖ **Moderate**	Included less than 100 participants per group
**SEBT Posteromedial; 5 studies**		⊕⊕⊕⊖ **Moderate**	Included less than 100 participants per group

Abbreviations: AP = anteroposterior; ML = mediolateral; RoB = risk of bias; SEBT = Star Excursion Balance Test.

Additional file 4C. Detailed GRADE description for the evidence level assessment in meta-analyses for Q3 (Balance x No Balance).

Analysis		Level of Evidence	Reason to downgrade the level (GRADE)
**Pain; 14 studies**		⊕⊕⊝⊝ **Low**	Included >25% of studies with high RoB (5 studies), heterogenic outcomes
Adjustment for publication bias	Trim-and-fill; 17 studies	⊕⊕⊝⊝ Low	Included >25% of studies with high RoB (5 studies + 1 imputed), heterogenic outcomes
	Removing outliers; 11 studies	⊕⊕⊝⊝ Low	Included >25% of studies with high RoB (4 studies), heterogenic outcomes
Subgroup analyses	***by study aim***		
	Balance specific; 4 studies	⊕⊝⊝⊝ Very Low	Included only studies with high RoB, heterogenic outcomes, included less than 100 participants per group
	Not specific; 10 studies	⊕⊕⊕⊝ Moderate	Heterogenic outcomes
	by comparator		
	Minimal interventions; 9 studies	⊕⊕⊝⊝ Low	Included >25% of studies with high RoB (3 studies), heterogenic outcomes
	Exercise; 5 studies	⊕⊕⊝⊝ Low	Included >25% of studies with high RoB (2 studies), heterogenic outcomes
	by interventions		
	Balance only; 2 studies	⊕⊝⊝⊝ Very Low	Included only studies with high RoB, heterogenic outcomes, included less than 100 participants per group
	Balance + exercise; 12 studies	⊕⊕⊕⊝ Moderate	Heterogenic outcomes
**Function (PROMs); 10 studies**		⊕⊕⊕⊝ **Moderate**	Heterogenic outcomes
Subgroup analyses	***by study aim***		
	Balance specific; 1 study	Not applicable	
	Not specific; 9 studies	⊕⊕⊕⊝ Moderate	Heterogenic outcomes
	by comparator		
	Minimal interventions; 6 studies	⊕⊕⊕⊝ Moderate	Heterogenic outcomes
	Exercise; 4 studies	⊕⊕⊝⊝ Low	Included >25% of studies with high RoB (1 study), heterogenic outcomes
	by interventions		
	Balance only; no study	Not applicable	
	Balance + exercise; 10 studies		
**Function (tests); 5 studies**		⊕⊝⊝⊝ **Very Low**	Included >25% of studies with high RoB (2 studies), heterogenic outcomes, included <100 participants per group

Abbreviations: PROMs = patient-reported outcome measures; RoB = risk of bias.
