# Supplementary material for: Is Postural Control Affected in People with Patellofemoral Pain and Should it be Part of Rehabilitation? A Systematic Review with Meta-analysis
Source: Sports Med Open. 2022 Dec 12;8:144. doi: 10.1186/s40798-022-00538-4 (PMC9742077; doi:10.1186/s40798-022-00538-4)
Supplement: Supplementary file 5 — Additional file 5. Study characteristics. [file 40798_2022_538_MOESM5_ESM.pdf]

Additional file 5A. Individual characteristics of the included studies for Question 1 (PFP x Control for Balance).

Study	Specific Population	Patellofemoral Pain Group							Control Group					Balance Assessment	Settings	Funding
		n (F/M)	*Age (y)*	*Symptom duration*	**Pain severity*	*H (m)*	*BM (kg)*	*BMI (kg/m²)*	*n (F/M)*	*Age (y)*	*H (m)*	*BM (kg)*	*BMI (kg/m²)*			
Akhbari et al. [89]	NI	15 (6/9)	25 (5)	NI	NI	1.72 (0.07)	74 (8)	NI	15 (6/9)	25 (6)	1.73 (0.08)	75 (9)	NI	Single-legged stance (Biodex Balance System)	NI	NI
Aminaka et al. [30]	University students	20 (12/8)	20 (2)	>8weeks	NI	1.70 (0.10)	72 (14)	NI	20 (12/8)	21 (3)	1.72 (0.09)	71 (11)	NI	Star Excursion Balance Test	Research laboratory	NI
Arun et al. [96]	NI	10 (?)	NI	<4 weeks	NI	NI	NI	NI	10 (?)	NI	NI	NI	NI	Star Excursion Balance Test	NI	NI
Carry et al. [57]	Adolescent females	7 (7/0)	14 (1)	1 to 6 months	NI	NI	NI	17.4 (2.9)	7 (7/0)	14 (1)	NI	NI	18.4 (3.2)	CoP - single-legged squat (Force plate)	NI	NI
Carvalho-e-Silva et al. [87]	Females	25 (25/0)	25 (7)	34 (23) months	5.6 (1.6)	1.63 (0.08)	61 (10)	NI	25 (25/0)	24 (4)	1.63 (0.05)	59 (7)	NI	CoP - the step task (Force Plate)	NI	NI
Coelho et al. [98]	Patients from clinic	48 (18/30)	31 (6)	38 (1-180) months	4.8 (1.4)	1.70 (0.10)	75 (14)	25.3 (3.6)	48 (18/30)	32 (6)	1.70 (0.10)	75 (13)	25.4 (3.1)	Y-balance test	NI	Yes
Felicio et al. [88]	Females	15 (15/0)	23 (3)	>1 month	4.6 (1.1)	1.61 (0.03)	59 (4)	NI	15 (15/0)	23 (2)	1.60 (3)	54 (2)	NI	CoP - single-legged stance (Force plate)	NI	Yes
Goto et al. [25]	University community	14 (10/4)	21 (3)	>8 weeks	0.8 (1.1)	1.72 (0.10)	70 (9)	NI	14 (10/4)	21 (3)	1.70 (0.08)	70 (9)	NI	Star Excursion Balance Test	Research laboratory	NI
Gwynne [58]	Physically active people	30 (18/12)	31 (7)	>2 months	NI	1.70 (9)	71 (10)	NI	30 (15/15)	30 (9)	1.71 (10)	70 (14)	NI	CoP - single-limb squats (Force plate)	NI	NI
Ibrahim et al. [86]	Males	30 (0/30)	21 (1)	>1 month	NI	1.74 (0.04)	69 (4)	NI	30 (0/30)	21 (1)	1.73 (0.05)	70 (3)	NI	Single- and double-legged stance (Biodex Balance System)	NI	None
Kim et al. [99]	Females	19 (19/0)	23 (3)	>1 month	NI	1.63 (0.05)	59 (8)	NI	19 (19/0)	23 (2)	1.64 (0.05)	55 (7)	NI	Single-legged drop landing (Force plate)	NI	Yes
Lee et al. [29]	Females	22 (22/0)	27 (6)	>3 months	NI	1.69 (0.08)	65 (10)	NI	19 (19/0)	26 (5)	1.68 (0.06)	63 (7)	NI	CoP - step-down task (Force Platform)	Research laboratory	Yes
Loudon et al. [93]	NI	29 (19/10)	28 (5)	5 months (average)	NI	1.70 (0.11)	70 (16)	NI	11 (7/4)	30 (5)	1.69 (0.10)	70 (15)	NI	Balance and reach test (repetitions)	NI	Yes
Manojlovic et al. [100]	NI	18 (13/5)	25 (13)	9 (4) months	5.8 (1.2)	1.71 (0.10	67 (17)	NI	37 (26/11)	22 (9)	1.71 (0.09)	64 (12)	NI	CoP - single-legged stance (Force plate)	NI	Yes
Motealleh et al. [92]	Females	21 (21/0)	23 (4)	>4 months	4.7 (1.3)	1.61 (0.04)	57 (6)	21.7 (1.8)	21 (21/0)	24 (3)	1.62 (0.04)	57 (6)	21.6 (1.9)	CoP - seated position on unstable device (Force plate)	Research laboratory	Yes
Nasab et al. [91]	Patients from clinics	15 (10/5)	25 (6)	>6 months	5.1 (1.2)	1.71 (0.10)	64 (10)	NI	15 (10/5)	25 (5)	1.72 (0.10)	66 (11)	NI	CoP - double-legged stance (Force Platform)	NI	NI
Naserpour et al. [103]	NI	34 (17/17)	24 (3)	>3 months	NI	1.69 (0.09)	68 (15)	NI	34 (17/17)	23 (3)	1.67 (0.09)	64 (10)	NI	CoP - step-down task (Force Platform)	NI	Yes
Negahban et al. [90]	NI	15 (12/3)	26 (4)	29 (21) months	5.7 (1.1)	1.64 (0.11)	NI	22.7 (3.8)	15 (12/3)	25 (5)	1.64 (0.10)	NI	22.9 (3.9)	Single-legged stance (Biodex Balance System)	NI	Yes
Priore et al. [101]	Females	55 (55/0)	22 (3)	59 (23) months	5.0 (1.7)	1.61 (0.06)	60 (7)	22.9 (2.8)	40 (40/0)	22 (3)	1.61 (0.06)	57 (8)	22.1 (3.0)	Star Excursion Balance Test	NI	Yes
Saad et al. [27]	Females	15 (15/0)	23 (2)	>1 month	NI	1.60 (0.03)	59 (4)	NI	15 (15/0)	23 (2)	1.60 (0.03)	54 (2)	NI	CoP - stair negotiation (Force Platform)	NI	Yes
Silva et al. [26]	Females	29 (29/0)	22 (3)	>1 month	5.8 (2.0)	1.65 (0.06)	65 (9)	NI	25 (25/0)	22 (4)	1.65 (0.04)	64 (6)	NI	CoP - stair climbing (Force plate)	Research laboratory	Yes

Song et al. [94]	Females	16 (16/0)	26 (6)	>1 month	NI	1.64 (0.05)	56 (6)	NI	8 (8/0)	29 (6)	1.61 (0.06)	52 (6)	NI	Star Excursion Balance Test	Researc h laboratory	NI
Steinberg et al. [85]	Adolescent females	83 (83/0)	13 (1)	NI	NI	1.57 (0.07)	48 (9)	19.7 (2.2)	49 (49/0)	13 (1)	1.57 (0.07)	47 (8)	19.1 (2.5)	Y-balance test	NI	NI
Stensdotter et al. [97]	Females	17 (17/0)	28 (7)	78 (75) months	52.3 (3.0)[a]	1.67 (0.08)	63 (9)	NI	17 (17/0)	26 (5)	1.67 (0.04)	61 (4)	NI	CoP - double-legged stance on sliding platform (Force plate)	Research laboratory	Yes
Stensdotter et al. [104]	Females	17 (17/0)	27 (7)	77 (8) months	51.2 (3.0)[a]	1.67 (0.08)	63 (9)	NI	17 (17/0)	26 (5)	1.67 (0.04)	61 (4)	NI	CoP - double-legged stance on sliding platform (Force plate)	NI	Yes
Yelvar et al. [102]	Females	22 (22/0)	36 (3)	8 (5) months	7.6 (1.8)	NI	NI	25.4 (4.4)	22 (22/0)	36 (3)	1.68 (0.06)	63 (7)	25.9 (3.4)	Standing in different conditions (Interactive Balance System)	NI	None
Zamboti et al. [95]	Females	10 (10/0)	21 (1)	4 (1) years	4.0 (3 to 7)	1.54 (0.06)	60 (8)	21.8 (1.8)	10 (10/0)	22 (2)	1.64 (0.03)	63 (7)	23.3 (3.2)	- Single-legged stance (Force plate)<br>- Star Excursion Balance Test	NI	NI
Zeinalzadeh et al [24]	University students	28 (20/8)	24 (4)	NI	NI	1.68 (0.09)	64 (12)	21.8 (5.2)	28 (20/8)	22 (3)	1.66 (0.10)	59 (9)	21.5 (2.8)	CoP - single-legged stance (Force plate)	NI	NI

Abbreviations: F = female, M = male, H = height, BM = Body mass, BMI = body mass index, CoP = centre of pressure, NI = not informed

*In the baseline assessment using VAS

[a]Knee Injury and Osteoarthritis Outcome Score (KOOS)

Additional file 5B. Individual characteristics of the included studies for Question 2 (Interventions for Balance).

Study	*n (F/M)*	*Age (y)*	*Symptom duration*	**Pain severity*	*H (m)*	*BM (kg)*	*BMI (kg/m²)*	Intervention 1	Intervention 2	Intervention 3	Dose	Balance Assessment	Settings	Funding
Ahmadi et al. [111]	32 (0/32)	25 (2)	>6 weeks	6.3 (1.8)	1.75 (0.04)	76 (6)	24.8 (1.6)	Neurofeedback training (n=16)	No intervention (n=16)	x	3x/week for 12 weeks	Double-legged stance (Biodex Balance System)	Research laboratory	NI
Aminaka et al. [30]	20 (12/8)	20 (2)	>8weeks	NI	1.70 (0.10)	72 (14)	NI	McConnell patellar taping (n=20)	No tape (n=20)	x	Single application	Star Excursion Balance Test	Research laboratory	NI
Aytar et al. [115]	22 (22/0)	24 (3)	16 (10) months	5.2 (1.9)	NI	NI	21.2 (2.3)	Kinesio taping on the quadriceps and around the patella (n=12)	Sham kinesio taping on the quadriceps and around the patella (n=10)	x	Single application (45 min)	Posturography (Kinesthetic Ability Trainer)	NI	NI
Chevidikunnan et al. [116]	20 (20/0)	22 (2)	>4 weeks	6.4 (3.6)	1.60 (0.05)	67 (8)	NI	Strengthening exercises for the core, knee and hip muscles (n=10)	Strengthening exercises for the knee and hip muscles (n=10)	x	3x/week for 4 weeks	Star Excursion Balance Test	Clinical settings	Yes
Demirci et al. [118]	35 (35/0)	37 (8)	>2 months	3.8 (1.8)	1.64 (0.05)	67 (12)	25.1 (4.4)	Lower limb traction, knee mobilizations, and home exercises (n=18)	Kinesio taping on the quadriceps and around the patella, and home exercises (n=17)	x	2x/week for 2 weeks (exercise for 6 weeks)	Y-balance test	NI	NI
Ebrahimi et al. [113]	24 (24/0)	31 (6)	>6 months	5.1 (1.5)	1.64 (0.05)	68 (12)	25.2 (4.2)	Exercise using virtual games (n=12)	Written instructions on how to manage daily activities (n=12)	x	3x/week for 8 weeks	Star Excursion Balance Test	Clinical settings	Yes
Fang et al. [121]	54 (0/54)	21 (3)	NI	NI	1.79 (0.06)	76 (6)	23.7 (1.8)	Aquatic exercise using a high intensity interval training program (n=27)	Bicycling training using the Wingate sprint protocol (n=27)	x	3x/week for 8 weeks	Y-balance test	Clinical settings	None
Ferreira et al. [109]	40 (40/0)	24 (3)	NI	6.1 (1.8)	1.60 (0.06)	61 (12)	23.7 (4.2)	McConnell patellar taping (n=20)	Sham taping (n=20)	x	Single application	CoP - single-legged stance and squat (Force plate)	NI	NI
Foroughi et al. [28]	33 (33/0)	24 (2)	>3 months	6.2 (1.2)	1.63 (0.07)	58 (10)	21.9 (2.9)	Stretching, strengthening, and core postural exercises (n=17)	Stretching and strengthening exercises (n=16)	x	3x/weeks for 4 weeks	CoP - seated position on unstable device (Force plate)	Research laboratory	NI
Goel and Bhatia [112]	30 (mixed)	28 (5)	3 (1) months	NI	1.63 (0.05)	63 (6)	23.9 (1.1)	McConnell patellar taping (n=30)	Sham taping (n=30)	No tape (n=30)	Single application	Star Excursion Balance Test	NI	NI
Lee et al. [29]	20 (20/0)	27 (6)	>3 months	NI	1.69 (0.08)	65 (10)	NI	Hip brace (n=20)	No brace (n=20)	x	Single application	CoP - step-down task (Force plate)	Research laboratory	Yes
Loudon et al. [31]	29 (22/7)	27 (6)	>2 months	4.3 (1.9)	1.50 (0.03)	66 (3)	NI	Home-based: education, flexibility, aerobic, strength and balance exercises (n=9)	Supervised treatment: education, flexibility, aerobic, strength and balance exercises (n=9)	Educational pamphlet (n=11)	1-2x/weeks for 8 weeks	Balance and reach test	Research laboratory	NI

Mahmoud and Kamel [114]	60 (24/36)	25 (6)	>3 months	5.5 (1.9)	1.74 (0.07)	73 (6)	NI	Balance training and strengthening exercises for hip muscles (n=30)	Strengthening exercises for hip muscles (n=30)	x	3x/week for 6 weeks	Single-legged stance (Prokin system PK252)	NI	NI
Maryam et al. [105,106]	26 (26/0)	24 (3)	>2 months	3.3 (2.0)	NI	NI	21.6 (2.2)	Kinesio taping on thigh (n=13)	Kinesio taping on core muscles (n=13)	x	Single application	- Star Excursion Balance Test<br>- Reach test	NI	Yes
Miller et al. [117]	18 (6/12)	20 (1)	> 2 weeks	3.2 (1.9)	1.74 (0.09)	72 (10)	NI	Kinesio taping on the hip (n=6)	Lumbopelvic manipulation (n=6)	Sham taping (n=6)	Single application	Y-balance test	NI	NI
Motealleh et al. [62]	28 (28/0)	29 (6)	>2 months	6.3 (2.1)	1.60 (0.06)	59 (8)	22.9 (3.1)	Strengthening exercises for knee muscles, flexibility exercises, and core neuromuscular training (n=14)	Strengthening for knee muscles and flexibility exercises (n=14)	x	4 weeks	Y-balance test	Clinical settings	Yes
Motealleh et al. [108]	44 (?)	24 (4)	>3 months	5.6 (1.1)	1.67 (0.08)	61 (8)	21.9 (1.8)	Lumbopelvic manipulation (n=22)	Sham lumbopelvic manipulation (n=22)	x	Single application	Star Excursion Balance Test	Research laboratory	Yes
Ojaghi et al. [119]	34 (19/15)	24 (2)	>1 month	NI	1.68 (0.09)	62 (9)	NI	McConnell patellar taping (n=17)	Elastic bandage taping (n=17)	x	Single application	- Star Excursion Balance Test<br>- CoP - single-legged stance (Force plate)	NI	Yes
Sinaei et al. [120]	32 (32/0)	26 (6)	>2 months	4.7 (1.3)	1.61 (0.05)	58 (8)	NI	Facilitatory kinesio taping on the vastus medialis obliquus (n=16)	Inhibitory kinesio taping on the vastus lateralis (n=16)	x	Single application	Star Excursion Balance Test	NI	Yes
Song et al. [94]	16 (16/0)	26 (6)	>1 month	NI	1.64 (0.05)	56 (6)	NI	Kinesio taping around the thigh (n=16)	Sham taping (n=16)	No tape (n=16)	Single application	Star Excursion Balance Test	Research laboratory	NI
Steinberg et al. [107]	98 (98/0)	13 (1)	NI	4.9 (2.0)	1.57 (0.08)	48 (9)	19.4 (2.6)	Somatosensory ballet-related exercises (n=28)	Isometric exercises for knee hip muscles (n=41)	Stretching exercises (n=29)	3x/weeks for 12 weeks	Y-balance test	not informed	NI
Zarei et al. [110]	40 (40/0)	24 (6)	>3 months	6.0 (0.9)	1.60 (0.06)	54 (8)	NI	Dry needling, stretching and strengthening exercises (n=20)	Stretching and strengthening exercises (n=20)	x	5x/week for 4 weeks (dry needling 1x/week)	Star Excursion Balance Test	Clinical settings	NI

Abbreviations: F = female, M = male, H = height, BM = Body mass, BMI = body mass index, CoP = centre of pressure, NI = not informed

*In the baseline assessment using VAS

Additional file 5C. Individual characteristics of the included studies for Question 3 (Balance Exercise for Pain/Funciton).

Study	*n* *(F/M)*	*Age* *(y)*	*Symptom duration*	**Pain severity*	*H* *(m)*	*BM* *(kg)*	*BMI* *(kg/m²)*	Intervention 1	Intervention 2	Intervention 3	Dose	Balance Assessment	Settings	Funding
Boitrago et al. [61]	60 (60/0)	26 (6)	>3 months	6.7 (2.0)	1.64 (0.06)	63 (11)	NI	Strengthening exercises for the knee, hip and trunk muscles, and proprioceptive exercises (n=30); ***Balance exercises***: single-legged stance on stable and unstable surface	Educational, cognitive and behavioral guidance on health (n=30)	x	3x/week for 6 weeks	P: VAS<br>F: AKPS and KOS-ADLS	Clinical settings	None
Clark et al. [124]	81 (36/45)	28 (7)	>3 months	7.8 (3.9)	NI	NI	25.0 (4.5)	Strengthening exercises for the knee and hip muscles, stretching and balance exercises, and education (n=20); ***Balance exercise***: balance work using a trampet	McConnell patellar taping and education (n=19)	Educational leaflet only (n=22)<br>**Intervention 4:** intervention 1 and 2 (n=20)	For 3 months	P: VAS<br>F: WOMAC	NI	NI
Ebrahimi et al. [113]	24 (24/0)	31 (6)	>6 months	5.1 (1.5)	1.64 (0.05)	68 (12)	25.2 (4.2)	Exercise using virtual games (n=12); ***Balance exercise***: single-legged stance was an important movement in the games	Written instructions on how to manage daily activities (n=12)	x	3x/week for 8 weeks	P: VAS<br>F: AKPS and step-down test	Clinical settings	Yes
Emamvirdi et al. [125]	64 (64/0)	23 (6)	>8 weeks	6.1 (1.3)	1.64 (0.08)	59 (4)	21.7 (1.4)	Neuromuscular progressive exercises with feedback to control knee valgus (n=32); ***Balance exercise***: single-legged exercises on stable and unstable surfaces	Written instructions regarding movement corrections and tips for improving general health (n=32)	x	3x/week for 6 weeks	P: VAS<br>F: Hop tests	Research laboratory	NI
Ferber et al. [122]	199 (133/66)	29 (7)	28 (41) months	5.1 (1.7)	1.70 (0.09)	68 (14)	NI	Hip- and core-focussed exercises (n=111); ***Balance exercises***: single- and double-legged stance on unstable surface	Knee-focused exercises (=88)	x	3x/week for 6 weeks	P: VAS<br>F: AKPS	Research laboratory	Yes
Foroughi et al. [28]	33 (33/0)	24 (2)	>3 months	6.2 (1.2)	1.63 (0.07)	58 (10)	21.9 (2.9)	Stretching, strengthening, and core postural exercises (n=17); ***Balance exercises***: postural control on unstable seat	Stretching and strengthening exercises (n=16)	x	3x/week for 4 weeks	P: NRS<br>F: AKPS	Research laboratory	NI
Loudon et al. [31]	29 (22/7)	27 (6)	>2 months	4.3 (1.9)	1.50 (0.03)	66 (3)	NI	Home-based: education, flexibility, aerobic, strength and balance exercises (n=9); ***Balance exercises***: single-legged stance with open and closed eyes; balance and reach task	Supervised treatment: education, flexibility, aerobic, strength and balance exercises (n=9); ***Balance exercises***: single-legged stance with open and closed eyes; balance and reach task	Educational pamphlet (n=11)	1-2x/week for 8 weeks	P: VAS<br>F: AKPS and functional tests	Research laboratory	NI
Mahmoud and Kamel [114]	60 (24/36)	25 (6)	>3 months	5.5 (1.9)	1.74 (0.07)	73 (6)	NI	Balance training and strengthening exercises for the hip muscles (n=30); ***Balance exercises***: double- and single-legged stance with open and closed eyes on stable and unstable surfaces, double- and single-legged dynamic activities on stable and unstable surfaces	Strengthening exercises for the hip muscles (n=30)	x	3x/week for 6 weeks	P: VAS<br>F: -	NI	NI

Molgaard et al. [127]	32 (22/10)	31 (11)	65 (41) months	6.6 (1.2)	1.73 (0.11)	76 (18)	25.7 (4.7)	Foot exercises, foot orthoses, strengthening exercises for knee muscles, manual therapy, and taping (n=17); ***Balance exercises***: double-legged stance on wobble board	Strengthening exercises for knee muscles, manual therapy, and taping (n=15)	-	1x/week for 3 months	P: KOOS pain subscale<br>F: KOOS ADL and Sport subscales	Clinical settings	Yes
Rabelo et al. [32]	34 (34/0)	26 (7)	48 (36) months	6.4 (1.2)	1.61 (0.07)	57 (7)	22.3 (2.4)	Movement control training of the trunk and lower limbs, and strengthening exercises for the knee and hip muscles (n=17); ***Balance exercise***: single-legged stance	Strengthening exercises for the knee and hip muscles (n=17)	-	3x/week for 4 weeks	P: NRS<br>F: AKPS	Research laboratory	None
Shadloo et al. [126]	30 (13/17)	28 (3)	>1 year	7.5 (2.1)	1.77 (0.04)	70 (4)	NI	Stretching and strengthening exercises for the knee muscles, and balance treining (n=15); ***Balance exercises***: single-legged stance and tilt board exercises with open and closed eyes	Whole-body vibration (n=15)	-	3x/week for 4 weeks	P: VAS<br>F: AKPS and leg press	Clinical settings	Yes
Steinberg et al. [107]	98 (98/0)	13 (1)	NI	4.9 (2.0)	1.57 (0.08)	48 (9)	19.4 (2.6)	Somatosensory ballet-related exercises (n=28); ***Balance exercises***: single-legged exercises on stable and unstable surfaces	Isometric exercises for knee hip muscles (n=41)	Stretching exercises (n=29)	3x/week for 12 weeks	P: VAS<br>F: -	NI	NI
van Linschoten et al. [123]	131 (84/47)	24 (8)	>2 months	6.2 (2.2)	NI	NI	23.1 (3.6)	Neuromuscular exercises targeting knee and hip muscles, including flexibility and balance exercises (n=65); ***Balance exercise***: not described	Usual care (n=66)	-	Daily for 3 months	P: VAS<br>F: AKPS	Clinical settings	Yes
Yalfani et al. [128]	32 (0/32)	25 (2)	>6 weeks	6.5 (1.6)	1.74 (0.04)	74 (6)	24.4 (1.5)	Sensorimotor exercises (n=16); ***Balance exercises***: double- and single-legged stance with open and closed eyes on stable and unstable surfaces, double- and single-legged dynamic activities on stable and unstable surfaces	No intervention (n=16)	-	3x/week for 12 weeks	P: VAS<br>F: -	Research laboratory	Yes

Abbreviations: F = female, M = male, H = height, BM = Body mass, BMI = body mass index, CoP = centre of pressure, NI = not informed; P = Pain; F = Function; NRS = Numeric Rating Scale; AKPS = Anterior Knee Pain Scale; VAS = Visual Analog Scale; WOMAC = The Western Ontario and McMaster Universities Osteoarthritis Index; KOOS = Knee injury and Osteoarthritis Outcome Score; KOS-ADLS = Knee Outcome Survey – Activities of Daily Living Scale
