# Supplementary material for: Is Postural Control Affected in People with Patellofemoral Pain and Should it be Part of Rehabilitation? A Systematic Review with Meta-analysis
Source: Sports Med Open. 2022 Dec 12;8:144. doi: 10.1186/s40798-022-00538-4 (PMC9742077; doi:10.1186/s40798-022-00538-4)
Supplement: Supplementary file 6 — Additional file 6. Risk of bias assessment. [file 40798_2022_538_MOESM6_ESM.pdf]

Additional file 6A. Risk of Bias for cross-sectional design data (Question 1).

	1. Study objective report	2. Balance measurement report	3. Study design report	4. Population source and sampling frame report	5. Eligibility criteria report	6. Participation rate report	7. Characteristics of participants	8. Statistical methods report	9. Balance findings report	10. Data variability of balance findings report	11. Statistical parameters report	12. Sample size calculations report	13. Comparability between groups	14. Adequate participation rate	15. Recruitment period of groups report	16. Data of subject losses taken into account	17. Blinding of assessors	18. Reliability report of balance outcomes	19. Standardized measures report	20. Assessment period of groups report	21. Adjust for covariates (age/sex)	22. Report of data by subgroups	23. Generalizability of the study results to the eligible population	24. Generalizability of the study results to other relevant populations	**Total Quality Score (0 to 48)**	**Overall Quality Score (0 to 2)**	**RISK OF BIAS**
Motealleh et al. [92]																		U							40	1.67	L
Priore et al. [101]															U			U		U					38	1.58	L
Steinberg et al. [85]															U					U					37	1.54	L
Yelvar et al. [102]																		U							37	1.54	L
Carvalho-e-Silva et al. [87]															U			U		U					36	1.50	L
Manojlovic et al. [100]															U			U		U					34	1.42	L
Song et al. [94]															U					U					34	1.42	L
Zamboti et al. [95]															U			U		U					34	1.42	L
Felicio et al. [88]															U			U		U					33	1.38	L
Negahban et al. [90]															U					U					33	1.38	L
Zeinalzadeh et al. [24]															U					U					32	1.33	L
Akhbari et al. [89]															U					U					31	1.29	L
Gwynne [58]															U			U		U					31	1.29	L
Lee et al. [29]															U			U		U					31	1.29	L
Coelho et al. [98]						U								U	U	U				U			U	U	30	1.25	L
Saad et al. [27]															U			U		U					29	1.21	L
Aminaka et al. [30]						U								U	U	U		U		U			U	U	26	1.08	L
Kim et al. [99]															U			U		U					26	1.08	L
Silva et al. [26]						U								U		U		U		U			U	U	26	1.08	L
Goto et al. [25]						U								U	U	U		U		U			U	U	25	1.04	L
Stensdotter et al. [104]															U			U		U					25	1.04	L
Loudon et al. [93]						U								U	U	U				U			U	U	24	1.00	H
Carry et al. [57]						U								U	U	U		U		U			U	U	22	0.92	H
Nasab et al. [91]						U								U	U	U		U		U			U	U	22	0.92	H
Ibrahim et al. [86]						U								U	U	U		U		U			U	U	20	0.83	H
Naserpour et al. [103]						U								U	U	U		U		U			U	U	20	0.83	H
Arun et al. [96]						U	U						U	U	U	U		U		U			U	U	18	0.75	H
Stensdotter et al. [97]						U								U	U	U		U		U			U	U	18	0.75	H

Black shading = yes, clear report; White shading = no, not reported; Gray shading = partial; U: unable to determine.

Abbreviations: H = high risk of bias; L = low risk of bias.

Additional file 6B. Risk of Bias for randomized controlled trails (Questions 2 and 3).

Study	1	2	3	4	5	6	Total	*Risk of Bias*
Question 2								
Zarei et al. [110]	■	■	■	■	■	■	6	Low
Motealleh et al. [62]	■	■	■	■	■		5	Low
Ebrahimi et al. [113]	■	■	■	■	■		5	Low
Aytar et al. [115]	■		■	■	■		4	Moderate
Chevidikunnan et al. [116]	■	■	■		■		4	Moderate
Ferreira et al. [109]	■	■	■		■		4	Moderate
Miller et al. [117]	■		■	■	■		4	Moderate
Motealleh et al. [108]	■	■	■		■		4	Moderate
Sinaei et al. [120]	■		■	■	■		4	Moderate
Demirci et al. [118]	■		■		■		3	Moderate
Mahmoud and Kamel [114]	■		■	■			3	Moderate
Fang et al. [121]	■		■				2	High
Foroughi et al. [28]	■		■				2	High
Lee et al. [29]	■				■		2	High
Maryam et al. [105,106]	■		■				2	High
Steinberg et al. [107]	■			■			2	High
Ojaghi et al. [119]	■		■				2	High
Song et al. [94]	■				■		2	High
Ahmadi et al. [111]	■						1	High
Aminaka et al. [30]	■						1	High
Goel and Bhatia [112]	■						1	High
Loudon et al. [31]	■						1	High
Question 3								
Rabelo et al. [32]	■	■	■	■	■	■	6	Low
Boitrago et al. [61]	■	■	■	■	■		5	Low
Clark et al. [124]	■		■	■	■	■	5	Low
Ebrahimi et al. [113]	■	■	■	■	■		5	Low
Emamvirdi et al. [125]	■	■	■		■	■	5	Low
Molgaard et al. [127]	■	■	■	■		■	5	Low
Shadloo et al. [126]	■	■	■	■	■		5	Low
Ferber et al. [122]	■	■	■			■	4	Moderate
van Linschoten et al. [123]	■		■		■	■	4	Moderate
Foroughi et al. [28]	■		■				2	High
Steinberg et al. [107]	■			■			2	High
Loudon et al. [31]	■						1	High
Mahmoud and Kamel [114]	■						1	High
Yalfani et al. [128]	■						1	High

Items PEDro Scale

1. participants randomly allocated to groups
2. allocation was concealed
3. similarity among groups at baseline
4. blinding of all assessors
5. results from more than 85% of allocated subjects
6. intention-to-treat approach

Black shading: item satisfied

White shading: item not satisfied
