# Supplementary material for: Is Postural Control Affected in People with Patellofemoral Pain and Should it be Part of Rehabilitation? A Systematic Review with Meta-analysis
Source: Sports Med Open. 2022 Dec 12;8:144. doi: 10.1186/s40798-022-00538-4 (PMC9742077; doi:10.1186/s40798-022-00538-4)
Supplement: Supplementary file 7 — Additional file 7. Subgroup and meta-regression analysis. [file 40798_2022_538_MOESM7_ESM.pdf]

Additional file 7A. Results for Subgroup and Meta-regression analyses for Q1 (PFP vs Controls for balance impairments).

Analysis		n PFP/ Control	SMD (95% CI)	p-value	I^2	Level of Evidence
AP postural stability; 19 studies		448/407	1.03 (0.40 to 1.66)	<0.001	86%	⊕⊖⊖⊖ Very Low
Subgroup analyses	***by sex***					
	Females; 8 studies	206/164	1.15 (–0.26 to 2.56)	0.110	90%	⊕⊕⊕⊖ Moderate
	Mixed; 10 studies	212/213	0.94 (0.27 to 1.60)	0.001	83%	⊕⊖⊖⊖ Very Low
	by assessment method					
	SEBT Anterior; 8 studies	230/170	1.08 (0.21 to 1.96)	0.015	85%	⊕⊕⊕⊖ Moderate
	Posturography; 4 studies	79/79	0.85 (0.37 to 1.32)	<0.001	49%	⊕⊕⊕⊖ Moderate
	CoP; 7 studies	139/158	1.19 (–0.44 to 2.83)	0.153	92%	⊕⊕⊖⊖ Low
	by task					
	Dynamic; 14 studies	351/291	1.26 (0.41 to 2.11)	0.004	87%	⊕⊖⊖⊖ Very Low
	Static; 5 studies	97/116	0.48 (–0.26 to 1.22)	0.206	83%	⊕⊕⊖⊖ Low
Meta-regression (age) = B=0.01, 95% CI –0.10 to 0.12; p=0.90; R^2=0%; I^2=86%						
ML postural stability; 13 studies		261/279	0.87 (0.31 to 1.42)	0.002	85%	⊕⊕⊕⊖ Moderate
Subgroup analyses	***by sex***					
	Females; 5 studies	100/99	0.88 (–0.18 to 1.93)	0.102	91%	⊕⊕⊖⊖ Low
	Mixed; 7 studies	131/150	0.83 (0.03 to 1.63)	0.042	79%	⊕⊕⊖⊖ Low
	by assessment method					
	Posturography; 4 studies	79/79	0.42 (–0.25 to 1.10)	0.222	78%	⊕⊕⊖⊖ Low
	CoP; 8 studies	172/190	0.84 (0.21 to 1.46)	0.009	86%	⊕⊕⊕⊖ Moderate
	by task					
	Dynamic; 9 studies	182/200	0.92 (0.13 to 1.71)	0.022	88%	⊕⊕⊕⊖ Moderate
	Static; 4 studies	79/79	0.81 (0.02 to 1.59)	0.044	80%	⊕⊕⊖⊖ Low
Meta-regression (age) = B=0.01, 95% CI –0.20 to 0.21; p=0.97; R^2=0%; I^2=84%						
Overall postural stability; 15 studies		354/335	0.38 (–0.05 to 0.82)	0.083	84%	⊕⊕⊕⊖ Moderate
Subgroup analyses	***by sex***					
	Females; 10 studies	218/199	0.31 (–0.33 to 0.96)	0.341	89%	⊕⊕⊕⊖ Moderate
	Mixed; 4 studies	106/106	0.30 (0.03 to 0.57)	0.032	0%	⊕⊕⊕⊖ High
	by assessment method					
	SEBT; 2 studies	103/88	0.51 (0.06 to 0.96)	0.027	58%	⊕⊕⊖⊖ Low
	Posturography; 5 studies	101/101	0.47 (0.01 to 0.93)	0.048	63%	⊕⊕⊕⊖ Moderate
	CoP; 8 studies	150/146	0.29 (–0.51 to 1.10)	0.475	91%	⊕⊕⊕⊖ Moderate
	by task					
	Dynamic; 10 studies	258/239	0.30 (–0.27 to 0.86)	0.301	87%	⊕⊕⊕⊖ Moderate
	Static; 5 studies	96/96	0.55 (–0.14 to 1.25)	0.119	79%	⊕⊕⊖⊖ Low
Meta-regression (age) = B=0.02, 95% CI –0.08 to 0.11; p=0.70; R^2=0%; I^2=86%						

Abbreviations: AP = anteroposterior; CoP = centre of pressure; ML = mediolateral; PFP = patellofemoral pain; SEBT = Star Excursion Balance Test; SMD = standardised mean difference.

- Balance for AP postural stability

Subgroup analysis: females

Study	PFP Total	PFP Mean	PFP SD	Control Total	Control Mean	Control SD	SMD	95%-CI	Weight
Steinberg et al. [85]	83	4.10	3.30	49	2.40	2.00	0.59	[0.22; 0.95]	13.0%
Carvalho-e-Silva et al. [87]	25	61.00	5.90	25	60.70	6.00	0.05	[-0.50; 0.60]	12.9%
Kim et al. [99]	19	0.01	0.00	19	0.00	0.00	1.24	[0.54; 1.94]	12.7%
Felicio et al. [88]	15	5.00	0.74	15	4.90	0.89	0.12	[-0.60; 0.84]	12.7%
Motealleh et al. [92]	21	26.04	11.48	21	9.35	4.02	1.90	[1.16; 2.64]	12.7%
Song et al. [94]	16	-65.57	4.83	8	-63.54	8.10	-0.32	[-1.18; 0.53]	12.5%
Zamboti et al. [95]	10	-80.50	4.48	10	-79.88	4.97	-0.13	[-1.00; 0.75]	12.5%
Stensdotter et al. [97]	17	3.10	0.03	17	2.90	0.03	6.51	[4.74; 8.28]	10.9%
**Random effects model**	**206**			**164**			**1.15**	**[-0.26; 2.56]**	**100.0%**

Heterogeneity: $I^2 = 90\%$, $\tau^2 = 3.9277$, $p < 0.01$

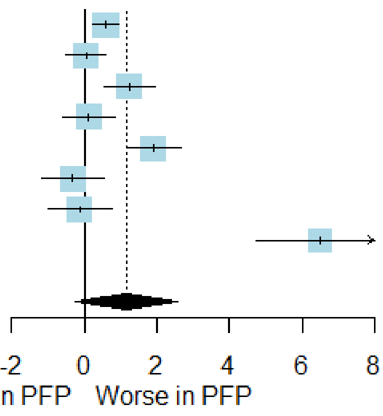

- Balance for AP postural stability

Subgroup analysis: mixed-sex

Study	PFP Total	PFP Mean	PFP SD	Control Total	Control Mean	Control SD	SMD	95%-CI	Weight
Coelho et al. [98]	48	-58.60	6.60	48	-61.70	5.90	0.49	[0.08; 0.90]	11.2%
Zeinalzadeh et al. [24]	28	3.59	0.97	28	3.29	1.17	0.28	[-0.25; 0.80]	10.9%
Manojlovic et al. [100]	18	5.00	1.60	37	5.60	1.50	-0.39	[-0.95; 0.18]	10.7%
Akhbari et al. [89]	15	1.90	1.20	15	1.60	1.10	0.25	[-0.47; 0.97]	10.2%
Negahban et al. [90]	15	2.83	1.47	15	2.12	0.88	0.57	[-0.16; 1.30]	10.2%
Nasab et al. [91]	15	0.22	0.14	15	0.15	0.09	0.58	[-0.15; 1.31]	10.2%
Loudon et al. [93]	29	-40.80	9.10	11	-53.60	6.70	1.47	[0.70; 2.24]	10.1%
Goto et al. [25]	14	-66.17	4.99	14	-70.84	4.39	0.96	[0.18; 1.75]	10.0%
Aminaka et al. [30]	20	-62.80	1.20	20	-65.60	1.20	2.29	[1.47; 3.10]	9.9%
Arun et al. [96]	10	-48.50	1.51	10	-53.90	0.88	4.18	[2.50; 5.87]	6.6%
**Random effects model**	**212**			**213**			**0.94**	**[0.27; 1.60]**	**100.0%**

Heterogeneity: $I^2 = 83\%$, $\tau^2 = 0.9821$, $p < 0.01$

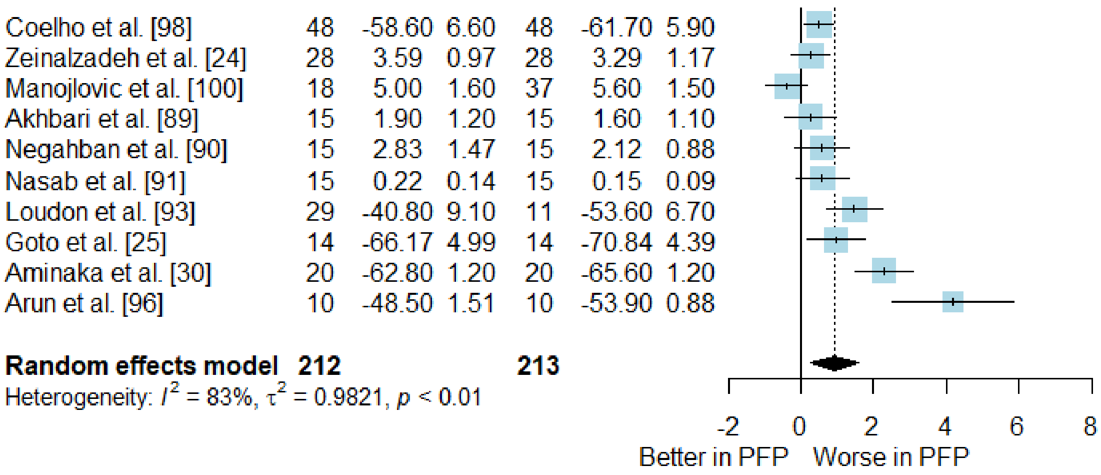

- Balance for AP postural stability

Subgroup analysis: Star Excursion Balance Test Anterior Direction

Study	PFP Total	PFP Mean	PFP SD	Control Total	Control Mean	Control SD	SMD	95%-CI	Weight
Steinberg et al. [85]	83	4.10	3.30	49	2.40	2.00	0.59	[0.22; 0.95]	13.9%
Coelho et al. [98]	48	-58.60	6.60	48	-61.70	5.90	0.49	[0.08; 0.90]	13.8%
Loudon et al. [93]	29	-40.80	9.10	11	-53.60	6.70	1.47	[0.70; 2.24]	12.8%
Goto et al. [25]	14	-66.17	4.99	14	-70.84	4.39	0.96	[0.18; 1.75]	12.7%
Aminaka et al. [30]	20	-62.80	1.20	20	-65.60	1.20	2.29	[1.47; 3.10]	12.6%
Song et al. [94]	16	-65.57	4.83	8	-63.54	8.10	-0.32	[-1.18; 0.53]	12.5%
Zamboti et al. [95]	10	-80.50	4.48	10	-79.88	4.97	-0.13	[-1.00; 0.75]	12.4%
Arun et al. [96]	10	-48.50	1.51	10	-53.90	0.88	4.18	[2.50; 5.87]	9.3%
**Random effects model**	**230**			**170**			**1.08**	**[0.21; 1.96]**	**100.0%**

Heterogeneity: $I^2 = 85\%$, $\tau^2 = 1.4013$, $p < 0.01$

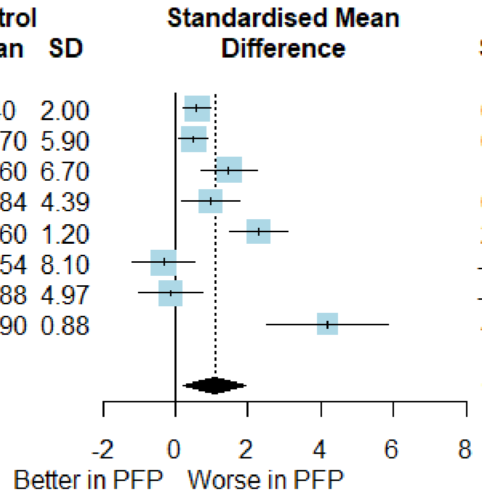

- Balance for AP postural stability

Subgroup analysis: Posturography

Study	PFP Total	PFP Mean	PFP SD	Control Total	Control Mean	Control SD	SMD	95%-CI	Weight
Ibrahim et al. [86]	30	3.30	2.30	30	1.30	0.30	1.20	[0.65; 1.76]	29.9%
Kim et al. [99]	19	0.01	0.00	19	0.00	0.00	1.24	[0.54; 1.94]	24.0%
Akhbari et al. [89]	15	1.90	1.20	15	1.60	1.10	0.25	[-0.47; 0.97]	23.3%
Negahban et al. [90]	15	2.83	1.47	15	2.12	0.88	0.57	[-0.16; 1.30]	22.8%
**Random effects model**	**79**			**79**			**0.85**	**[0.37; 1.32]**	**100.0%**

Heterogeneity: $I^2 = 49\%$, $\tau^2 = 0.1143$, $p = 0.12$

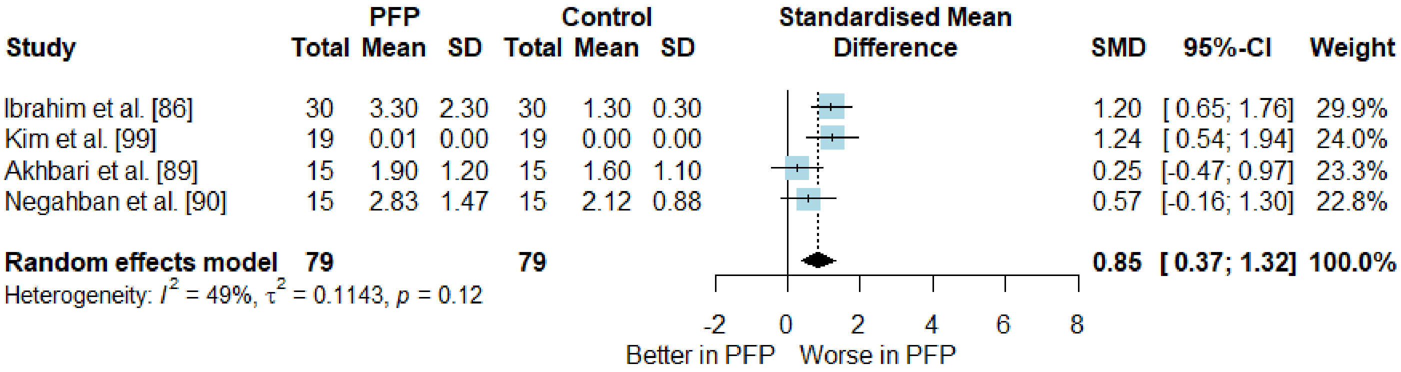

- Balance for AP postural stability

Subgroup analysis: Centre of Pressure Behavior

Study	PFP Total	PFP Mean	PFP SD	Control Total	Control Mean	Control SD	SMD	95%-CI	Weight
Zeinalzadeh et al. [24]	28	3.59	0.97	28	3.29	1.17	0.28	[-0.25; 0.80]	14.7%
Carvalho-e-Silva et al. [87]	25	61.00	5.90	25	60.70	6.00	0.05	[-0.50; 0.60]	14.6%
Manojlovic et al. [100]	18	5.00	1.60	37	5.60	1.50	-0.39	[-0.95; 0.18]	14.6%
Felicio et al. [88]	15	5.00	0.74	15	4.90	0.89	0.12	[-0.60; 0.84]	14.5%
Nasab et al. [91]	15	0.22	0.14	15	0.15	0.09	0.58	[-0.15; 1.31]	14.5%
Motealleh et al. [92]	21	26.04	11.48	21	9.35	4.02	1.90	[1.16; 2.64]	14.5%
Stensdotter et al. [97]	17	3.10	0.03	17	2.90	0.03	6.51	[4.74; 8.28]	12.7%
**Random effects model**	**139**			**158**			**1.19**	**[-0.44; 2.83]**	**100.0%**

Heterogeneity: $I^2 = 92\%$, $\tau^2 = 4.6707$, $p < 0.01$

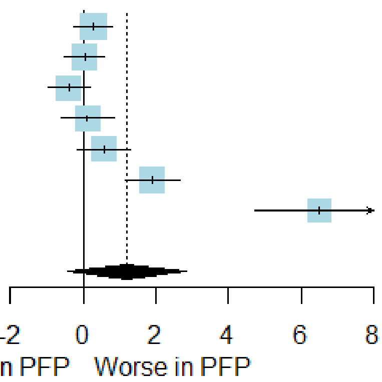

- Balance for AP postural stability

Subgroup analysis: Dynamic measures

Study	PFP Total	PFP Mean	PFP SD	Control Total	Control Mean	Control SD	SMD	95%-CI	Weight
Steinberg et al. [85]	83	4.10	3.30	49	2.40	2.00	0.59	[0.22; 0.95]	7.6%
Coelho et al. [98]	48	-58.60	6.60	48	-61.70	5.90	0.49	[0.08; 0.90]	7.6%
Ibrahim et al. [86]	30	3.30	2.30	30	1.30	0.30	1.20	[0.65; 1.76]	7.5%
Carvalho-e-Silva et al. [87]	25	61.00	5.90	25	60.70	6.00	0.05	[-0.50; 0.60]	7.5%
Kim et al. [99]	19	0.01	0.00	19	0.00	0.00	1.24	[0.54; 1.94]	7.4%
Akhbari et al. [89]	15	1.90	1.20	15	1.60	1.10	0.25	[-0.47; 0.97]	7.3%
Negahban et al. [90]	15	2.83	1.47	15	2.12	0.88	0.57	[-0.16; 1.30]	7.3%
Loudon et al. [93]	29	-40.80	9.10	11	-53.60	6.70	1.47	[0.70; 2.24]	7.3%
Goto et al. [25]	14	-66.17	4.99	14	-70.84	4.39	0.96	[0.18; 1.75]	7.3%
Aminaka et al. [30]	20	-62.80	1.20	20	-65.60	1.20	2.29	[1.47; 3.10]	7.2%
Song et al. [94]	16	-65.57	4.83	8	-63.54	8.10	-0.32	[-1.18; 0.53]	7.2%
Zamboti et al. [95]	10	-80.50	4.48	10	-79.88	4.97	-0.13	[-1.00; 0.75]	7.1%
Arun et al. [96]	10	-48.50	1.51	10	-53.90	0.88	4.18	[2.50; 5.87]	5.9%
Stensdotter et al. [97]	17	3.10	0.03	17	2.90	0.03	6.51	[4.74; 8.28]	5.8%
**Random effects model**	**351**			**291**			**1.26**	**[0.41; 2.11]**	**100.0%**

Heterogeneity: $I^2 = 87\%$, $\tau^2 = 2.4129$, $p < 0.01$

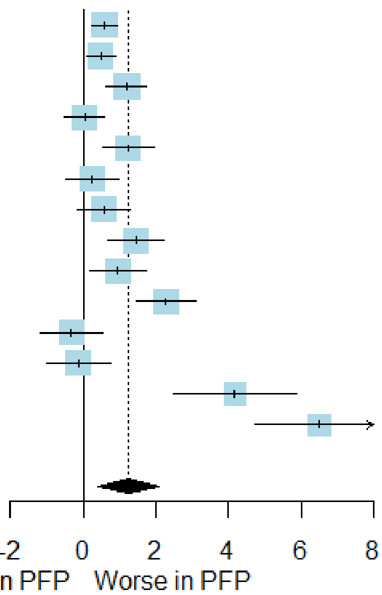

- Balance for AP postural stability

Subgroup analysis: Static measures

Study	PFP Total	PFP Mean	PFP SD	Control Total	Control Mean	Control SD	SMD	95%-CI	Weight
Zeinalzadeh et al. [24]	28	3.59	0.97	28	3.29	1.17	0.28	[-0.25; 0.80]	21.2%
Manojlovic et al. [100]	18	5.00	1.60	37	5.60	1.50	-0.39	[-0.95; 0.18]	20.8%
Felicio et al. [88]	15	5.00	0.74	15	4.90	0.89	0.12	[-0.60; 0.84]	19.4%
Nasab et al. [91]	15	0.22	0.14	15	0.15	0.09	0.58	[-0.15; 1.31]	19.3%
Motealleh et al. [92]	21	26.04	11.48	21	9.35	4.02	1.90	[1.16; 2.64]	19.2%
**Random effects model**	**97**			**116**			**0.48**	**[-0.26; 1.22]**	**100.0%**

Heterogeneity: $I^2 = 83\%$, $\tau^2 = 0.6032$, $p < 0.01$

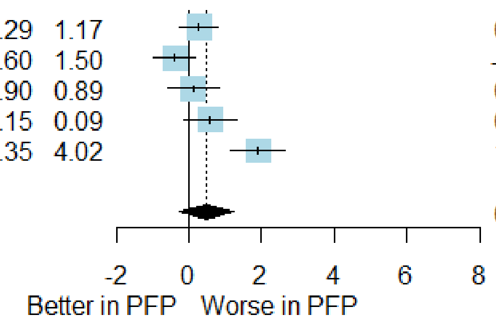

- Balance for ML postural stability

Subgroup analysis: females

Study	PFP Total	PFP Mean	PFP SD	Control Total	Control Mean	Control SD	SMD	95%-CI	Weight
Kim et al. [99]	19	0.01	0.00	19	0.01	0.00	-0.49	[-1.14; 0.16]	20.2%
Lee et al. [29]	20	24.70	16.30	19	13.50	4.40	0.91	[0.25; 1.57]	20.2%
Motealleh et al. [92]	21	25.47	12.40	21	9.70	3.70	1.69	[0.98; 2.40]	19.9%
Felicio et al. [88]	15	7.18	2.09	15	7.39	2.40	-0.09	[-0.81; 0.63]	19.9%
Carvalho-e-Silva et al. [87]	25	32.30	5.50	25	21.70	2.70	2.41	[1.67; 3.15]	19.8%
**Random effects model**	**100**			**99**			**0.88**	**[-0.18; 1.93]**	**100.0%**

Heterogeneity: $I^2 = 91\%$, $\tau^2 = 1.3214$, $p < 0.01$

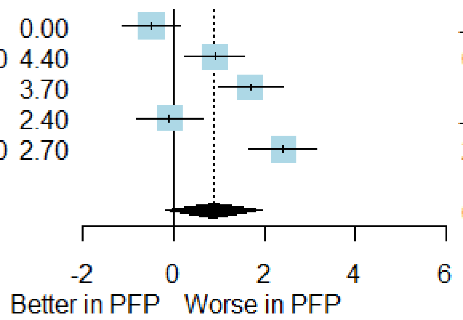

- Balance for ML postural stability

Subgroup analysis: mixed-sex

Study	PFP Total	PFP Mean	PFP SD	Control Total	Control Mean	Control SD	SMD	95%-CI	Weight
Gwynne [58]	30	1.50	0.15	30	1.43	0.10	0.54	[0.03; 1.06]	15.6%
Zeinalzadeh et al. [24]	28	2.89	1.06	28	2.54	0.55	0.41	[-0.12; 0.94]	15.5%
Manojlovic et al. [100]	18	5.90	2.20	37	6.60	2.40	-0.30	[-0.86; 0.27]	15.4%
Negahban et al. [90]	15	3.07	1.30	15	2.45	1.45	0.44	[-0.29; 1.16]	14.7%
Akhbari et al. [89]	15	1.70	0.70	15	1.30	0.60	0.60	[-0.14; 1.33]	14.6%
Nasab et al. [91]	15	0.34	0.21	15	0.14	0.05	1.27	[0.48; 2.07]	14.3%
Arun et al. [96]	10	-46.10	0.99	10	-52.70	2.00	4.01	[2.37; 5.64]	9.9%
**Random effects model**	**131**			**150**			**0.83**	**[0.03; 1.63]**	**100.0%**

Heterogeneity: $I^2 = 79\%$, $\tau^2 = 1.0021$, $p < 0.01$

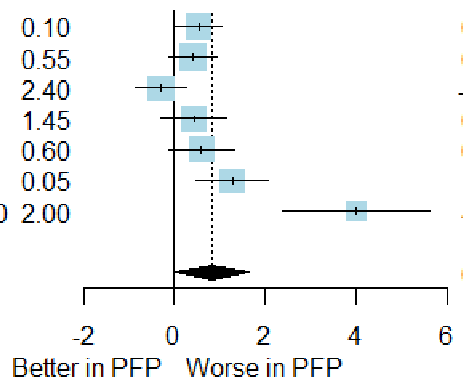

- Balance for ML postural stability

Subgroup analysis: Posturography

Study	PFP Total	PFP Mean	PFP SD	Control Total	Control Mean	Control SD	SMD	95%-CI	Weight
Ibrahim et al. [86]	30	3.00	1.80	30	1.50	0.60	1.10	[0.56; 1.65]	27.1%
Kim et al. [99]	19	0.01	0.00	19	0.01	0.00	-0.49	[-1.14; 0.16]	25.3%
Negahban et al. [90]	15	3.07	1.30	15	2.45	1.45	0.44	[-0.29; 1.16]	23.9%
Akhbari et al. [89]	15	1.70	0.70	15	1.30	0.60	0.60	[-0.14; 1.33]	23.7%
**Random effects model**	**79**			**79**			**0.42**	**[-0.25; 1.10]**	**100.0%**

Heterogeneity: $I^2 = 78\%$, $\tau^2 = 0.3610$, $p < 0.01$

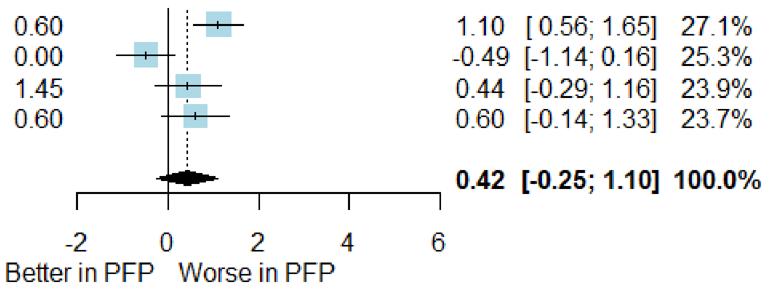

- Balance for ML postural stability

Subgroup analysis: Centre of Pressure Behavior

Study	PFP Total	PFP Mean	PFP SD	Control Total	Control Mean	Control SD	SMD	95%-CI	Weight
Gwynne [58]	30	1.50	0.15	30	1.43	0.10	0.54	[0.03; 1.06]	13.2%
Zeinalzadeh et al. [24]	28	2.89	1.06	28	2.54	0.55	0.41	[-0.12; 0.94]	13.1%
Manojlovic et al. [100]	18	5.90	2.20	37	6.60	2.40	-0.30	[-0.86; 0.27]	13.0%
Lee et al. [29]	20	24.70	16.30	19	13.50	4.40	0.91	[0.25; 1.57]	12.5%
Motealleh et al. [92]	21	25.47	12.40	21	9.70	3.70	1.69	[0.98; 2.40]	12.2%
Felicio et al. [88]	15	7.18	2.09	15	7.39	2.40	-0.09	[-0.81; 0.63]	12.2%
Carvalho-e-Silva et al. [87]	25	32.30	5.50	25	21.70	2.70	2.41	[1.67; 3.15]	12.1%
Nasab et al. [91]	15	0.34	0.21	15	0.14	0.05	1.27	[0.48; 2.07]	11.8%
**Random effects model**	**172**			**190**			**0.84**	**[0.21; 1.46]**	**100.0%**

Heterogeneity: $I^2 = 86\%$, $\tau^2 = 0.6965$, $p < 0.01$

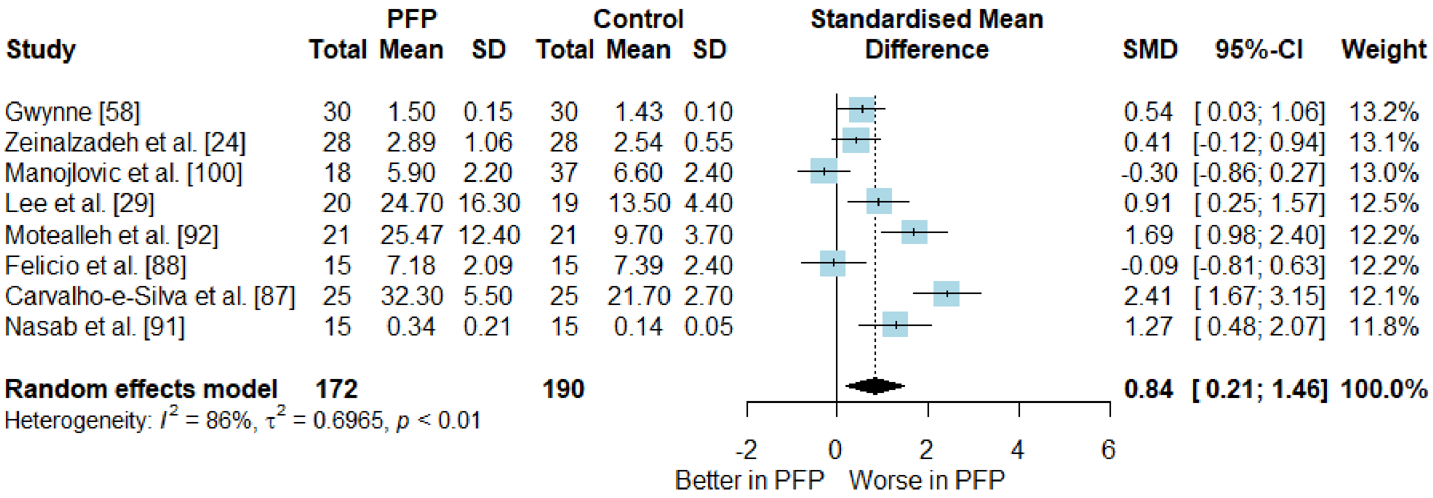

- Balance for ML postural stability

Subgroup analysis: Dynamic measures

Study	PFP Total	PFP Mean	PFP SD	Control Total	Control Mean	Control SD	SMD	95%-CI	Weight
Gwynne [58]	30	1.50	0.15	30	1.43	0.10	0.54	[0.03; 1.06]	11.8%
Ibrahim et al. [86]	30	3.00	1.80	30	1.50	0.60	1.10	[0.56; 1.65]	11.7%
Manojlovic et al. [100]	18	5.90	2.20	37	6.60	2.40	-0.30	[-0.86; 0.27]	11.7%
Kim et al. [99]	19	0.01	0.00	19	0.01	0.00	-0.49	[-1.14; 0.16]	11.5%
Lee et al. [29]	20	24.70	16.30	19	13.50	4.40	0.91	[0.25; 1.57]	11.4%
Negahban et al. [90]	15	3.07	1.30	15	2.45	1.45	0.44	[-0.29; 1.16]	11.3%
Akhbari et al. [89]	15	1.70	0.70	15	1.30	0.60	0.60	[-0.14; 1.33]	11.2%
Carvalho-e-Silva et al. [87]	25	32.30	5.50	25	21.70	2.70	2.41	[1.67; 3.15]	11.2%
Arun et al. [96]	10	-46.10	0.99	10	-52.70	2.00	4.01	[2.37; 5.64]	8.1%
**Random effects model**	**182**			**200**			**0.92**	**[0.13; 1.71]**	**100.0%**

Heterogeneity: $I^2 = 88\%$, $\tau^2 = 1.3002$, $p < 0.01$

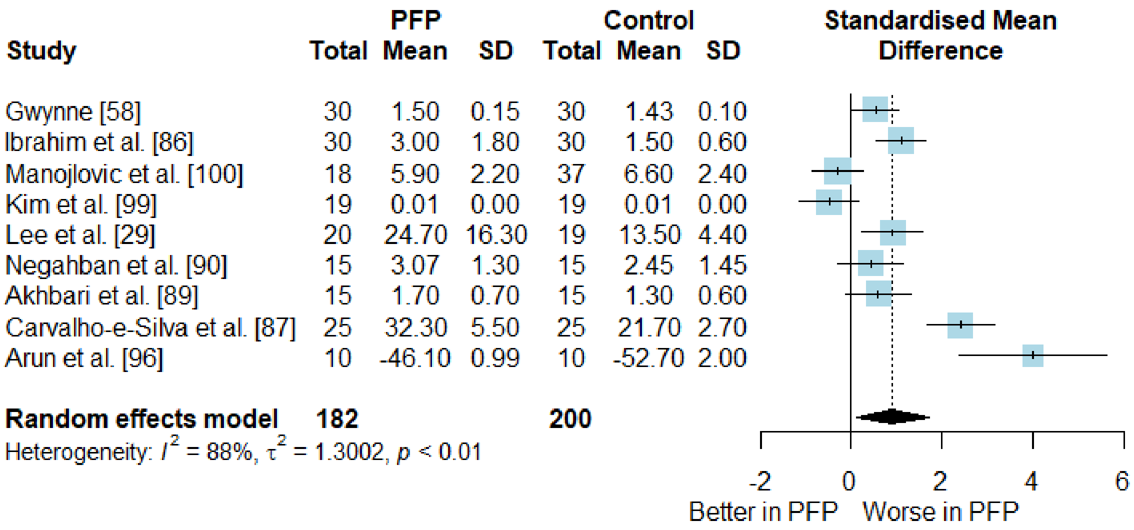

- Balance for ML postural stability

Subgroup analysis: Static measures

Study	PFP Total	PFP Mean	PFP SD	Control Total	Control Mean	Control SD	SMD	95%-CI	Weight
Zeinalzadeh et al. [24]	28	2.89	1.06	28	2.54	0.55	0.41	[-0.12; 0.94]	27.2%
Motealleh et al. [92]	21	25.47	12.40	21	9.70	3.70	1.69	[0.98; 2.40]	24.7%
Felicio et al. [88]	15	7.18	2.09	15	7.39	2.40	-0.09	[-0.81; 0.63]	24.6%
Nasab et al. [91]	15	0.34	0.21	15	0.14	0.05	1.27	[0.48; 2.07]	23.5%
**Random effects model**	**79**			**79**			**0.81**	**[0.02; 1.59]**	**100.0%**

Heterogeneity: $I^2 = 80\%$, $\tau^2 = 0.5181$, $p < 0.01$

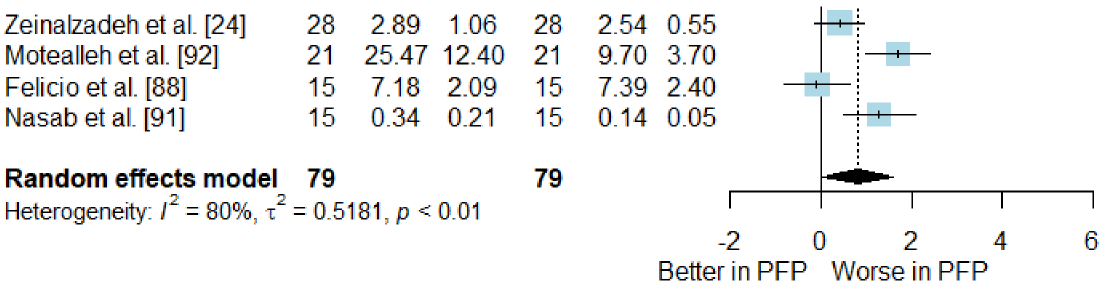

- Balance for Overall postural stability

Subgroup analysis: females

Study	PFP Total	PFP Mean	PFP SD	Control Total	Control Mean	Control SD	SMD	95%-CI	Weight
Priore et al. [101]	55	-78.00	8.00	40	-84.00	8.00	0.74	[0.32; 1.17]	10.9%
Carvalho-e-Silva et al. [87]	25	888.70	76.10	25	812.90	69.20	1.03	[0.43; 1.62]	10.4%
Yelvar et al. [102]	22	12.16	3.14	22	11.09	3.99	0.29	[-0.30; 0.89]	10.4%
Kim et al. [99]	19	0.04	0.01	19	0.04	0.01	-0.19	[-0.82; 0.45]	10.3%
Silva et al. [26]	29	26.40	7.50	25	43.10	10.20	-1.86	[-2.51; -1.21]	10.2%
Felicio et al. [88]	15	16.12	4.66	15	16.52	4.43	-0.09	[-0.80; 0.63]	10.0%
Motealleh et al. [92]	21	564.60	263.70	21	186.38	68.04	1.93	[1.18; 2.67]	9.9%
Saad et al. [27]	15	56.63	24.56	15	36.34	10.92	1.04	[0.27; 1.81]	9.8%
Zamboti et al. [95]	10	7.93	2.98	10	6.41	1.50	0.62	[-0.28; 1.52]	9.3%
Carry et al. [57]	7	5459.89	1627.74	7	6131.39	1240.87	-0.43	[-1.50; 0.63]	8.7%
**Random effects model**	**218**			**199**			**0.31**	**[-0.33; 0.96]**	**100.0%**

Heterogeneity: $I^2 = 89\%$, $\tau^2 = 0.9457$, $p < 0.01$

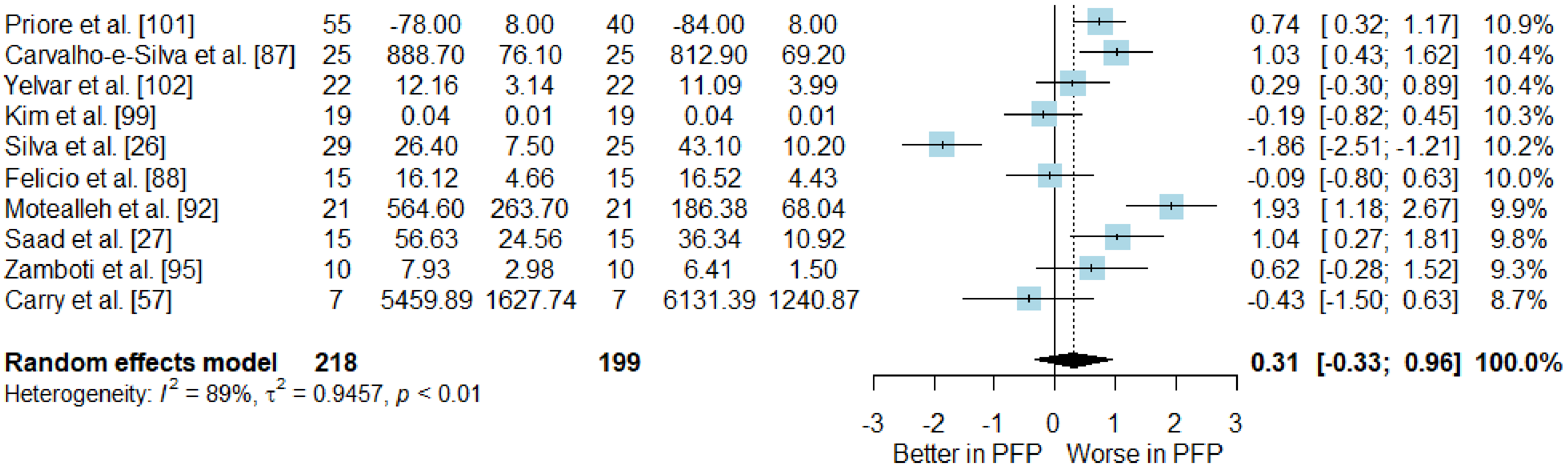

- Balance for Overall postural stability

Subgroup analysis: mixed-sex

Study	PFP Total	PFP Mean	PFP SD	Control Total	Control Mean	Control SD	SMD	95%-CI	Weight
Coelho et al. [98]	48	-83.80	9.30	48	-86.10	6.50	0.28	[-0.12; 0.69]	45.5%
Zeinalzadeh et al. [24]	28	40.36	19.29	28	37.69	28.44	0.11	[-0.42; 0.63]	26.8%
Akhbari et al. [89]	15	2.70	1.30	15	2.20	0.90	0.44	[-0.29; 1.16]	14.0%
Negahban et al. [90]	15	4.54	1.77	15	3.63	1.35	0.56	[-0.17; 1.29]	13.7%
**Random effects model**	**106**			**106**			**0.30**	**[0.03; 0.57]**	**100.0%**

Heterogeneity: $I^2 = 0\%$, $\tau^2 = 0$, $p = 0.77$

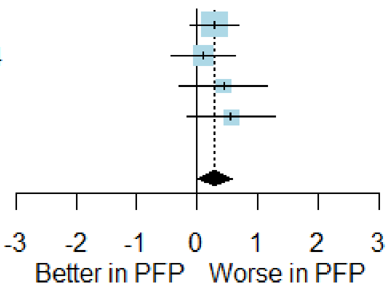

- Balance for Overall postural stability

Subgroup analysis: SEBT

Study	PFP Total	PFP Mean	PFP SD	Control Total	Control Mean	Control SD	SMD	95%-CI	Weight
Coelho et al. [98]	48	-83.80	9.30	48	-86.10	6.50	0.28	[-0.12; 0.69]	51.0%
Priore et al. [101]	55	-78.00	8.00	40	-84.00	8.00	0.74	[0.32; 1.17]	49.0%
**Random effects model**	**103**			**88**			**0.51**	**[0.06; 0.96]**	**100.0%**

Heterogeneity: $I^2 = 58\%$, $\tau^2 = 0.0614$, $p = 0.12$

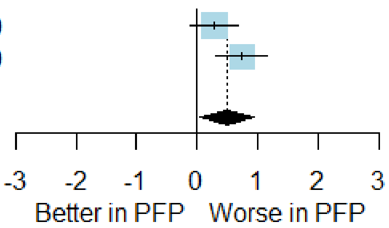

- Balance for Overall postural stability

Subgroup analysis: Posturography

Study	PFP Total	PFP Mean	PFP SD	Control Total	Control Mean	Control SD	SMD	95%-CI	Weight
Ibrahim et al. [86]	30	4.90	3.10	30	2.20	0.70	1.19	[0.63; 1.74]	22.3%
Yelvar et al. [102]	22	12.16	3.14	22	11.09	3.99	0.29	[-0.30; 0.89]	21.3%
Kim et al. [99]	19	0.04	0.01	19	0.04	0.01	-0.19	[-0.82; 0.45]	20.2%
Akhbari et al. [89]	15	2.70	1.30	15	2.20	0.90	0.44	[-0.29; 1.16]	18.2%
Negahban et al. [90]	15	4.54	1.77	15	3.63	1.35	0.56	[-0.17; 1.29]	18.0%
**Random effects model**	**101**			**101**			**0.47**	**[0.01; 0.93]**	**100.0%**

Heterogeneity: $I^2 = 63\%$, $\tau^2 = 0.1725$, $p = 0.03$

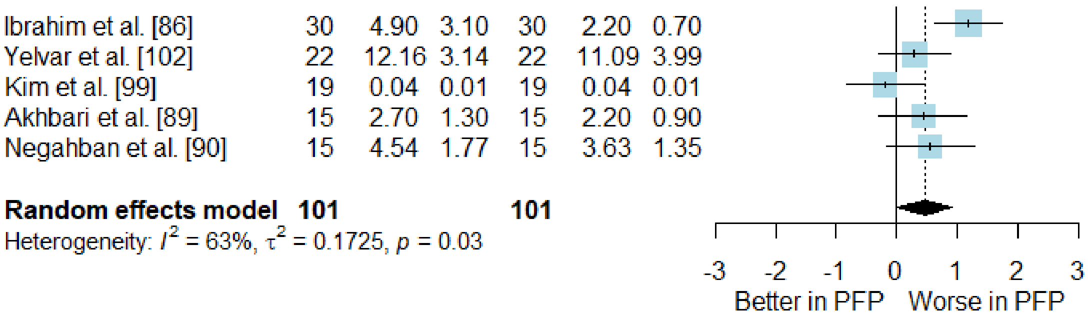

- Balance for Overall postural stability

Subgroup analysis: Centre of Pressure Behavior

Study	PFP Total	PFP Mean	PFP SD	Control Total	Control Mean	Control SD	SMD	95%-CI	Weight
Zeinalzadeh et al. [24]	28	40.36	19.29	28	37.69	28.44	0.11	[-0.42; 0.63]	13.3%
Carvalho-e-Silva et al. [87]	25	888.70	76.10	25	812.90	69.20	1.03	[0.43; 1.62]	13.0%
Silva et al. [26]	29	26.40	7.50	25	43.10	10.20	-1.86	[-2.51; -1.21]	12.9%
Felicio et al. [88]	15	16.12	4.66	15	16.52	4.43	-0.09	[-0.80; 0.63]	12.6%
Motealleh et al. [92]	21	564.60	263.70	21	186.38	68.04	1.93	[1.18; 2.67]	12.5%
Saad et al. [27]	15	56.63	24.56	15	36.34	10.92	1.04	[0.27; 1.81]	12.4%
Zamboti et al. [95]	10	7.93	2.98	10	6.41	1.50	0.62	[-0.28; 1.52]	11.9%
Carry et al. [57]	7	5459.89	1627.74	7	6131.39	1240.87	-0.43	[-1.50; 0.63]	11.3%
**Random effects model**	**150**			**146**			**0.29**	**[-0.51; 1.10]**	**100.0%**

Heterogeneity: $I^2 = 91\%$, $\tau^2 = 1.2022$, $p < 0.01$

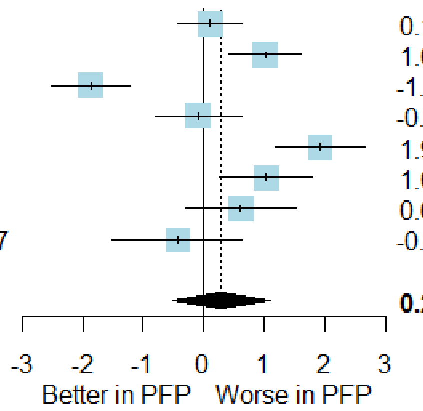

- Balance for Overall postural stability

Subgroup analysis: Dynamic measures

Study	PFP Total	PFP Mean	PFP SD	Control Total	Control Mean	Control SD	SMD	95%-CI	Weight
Coelho et al. [98]	48	-83.80	9.30	48	-86.10	6.50	0.28	[-0.12; 0.69]	10.9%
Priore et al. [101]	55	-78.00	8.00	40	-84.00	8.00	0.74	[0.32; 1.17]	10.9%
Ibrahim et al. [86]	30	4.90	3.10	30	2.20	0.70	1.19	[0.63; 1.74]	10.4%
Carvalho-e-Silva et al. [87]	25	888.70	76.10	25	812.90	69.20	1.03	[0.43; 1.62]	10.3%
Kim et al. [99]	19	0.04	0.01	19	0.04	0.01	-0.19	[-0.82; 0.45]	10.1%
Silva et al. [26]	29	26.40	7.50	25	43.10	10.20	-1.86	[-2.51; -1.21]	10.1%
Akhbari et al. [89]	15	2.70	1.30	15	2.20	0.90	0.44	[-0.29; 1.16]	9.7%
Negahban et al. [90]	15	4.54	1.77	15	3.63	1.35	0.56	[-0.17; 1.29]	9.7%
Saad et al. [27]	15	56.63	24.56	15	36.34	10.92	1.04	[0.27; 1.81]	9.6%
Carry et al. [57]	7	5459.89	1627.74	7	6131.39	1240.87	-0.43	[-1.50; 0.63]	8.2%
**Random effects model**	**258**			**239**			**0.30**	**[-0.27; 0.86]**	**100.0%**

Heterogeneity: $I^2 = 87\%$, $\tau^2 = 0.7265$, $p < 0.01$

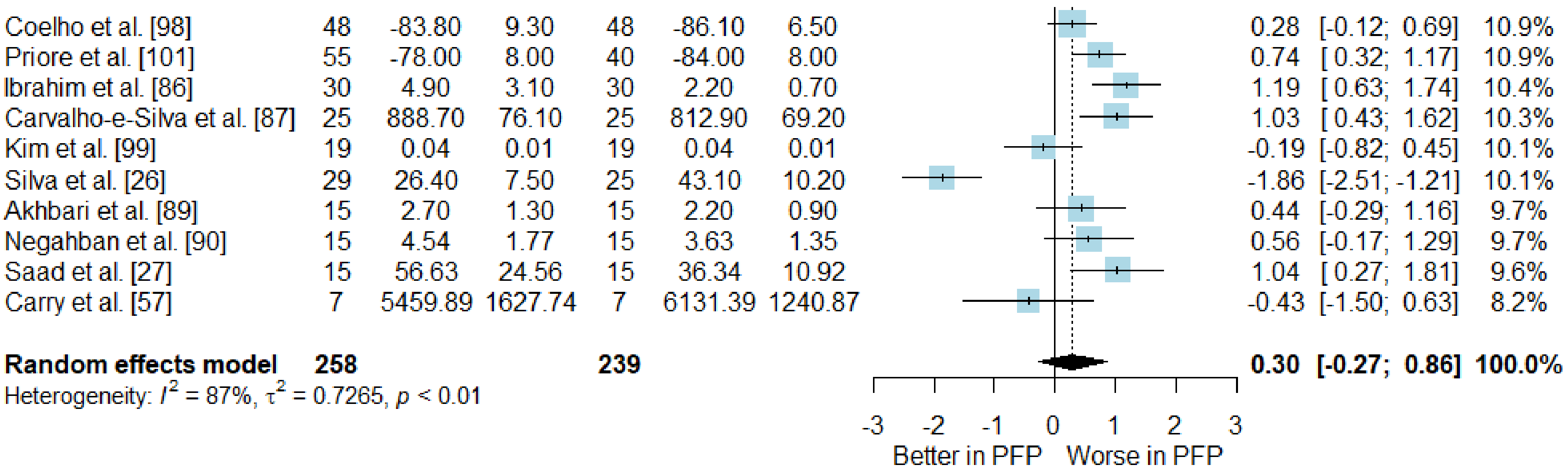

- Balance for Overall postural stability

Subgroup analysis: Static measures

Study	PFP Total	PFP Mean	PFP SD	Control Total	Control Mean	Control SD	SMD	95%-CI	Weight
Zeinalzadeh et al. [24]	28	40.36	19.29	28	37.69	28.44	0.11	[-0.42; 0.63]	21.9%
Yelvar et al. [102]	22	12.16	3.14	22	11.09	3.99	0.29	[-0.30; 0.89]	21.2%
Felicio et al. [88]	15	16.12	4.66	15	16.52	4.43	-0.09	[-0.80; 0.63]	19.8%
Motealleh et al. [92]	21	564.60	263.70	21	186.38	68.04	1.93	[1.18; 2.67]	19.5%
Zamboti et al. [95]	10	7.93	2.98	10	6.41	1.50	0.62	[-0.28; 1.52]	17.6%
**Random effects model**	**96**			**96**			**0.55**	**[-0.14; 1.25]**	**100.0%**

Heterogeneity: $I^2 = 79\%$, $\tau^2 = 0.5006$, $p < 0.01$

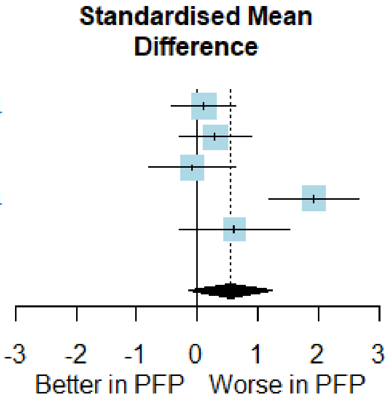

Additional file 7B. Results for Subgroup and Meta-regression analyses for Q2 (Interventions for balance improvement)

	Analysis	n Exp/Con	SMD (95% CI)	p-value	I^2	Level of Evidence
AP postural stability;11 studies		215/215	0.59 (0.04 to 1.14)	0.038	84%	⊕⊕⊖⊖ Low
Subgroup analyses	***by intervention characteristic***					
	Passive; 7 studies	144/144	0.67 (–0.19 to 1.52)	0.126	89%	⊕⊕⊖⊖ Low
	Exercise; 4 studies	71/71	0.48 (–0.03 to 0.99)	0.063	54%	⊕⊖⊖⊖ Very Low
	by comparator					
	Sham/no intervention; 8 studies	164/165	0.63 (–0.12 to 1.38)	0.102	88%	⊕⊕⊖⊖ Low
	Exercise; 3 studies	51/50	0.49 (–0.03 to 1.00)	0.065	40%	⊕⊕⊖⊖ Low
	by design					
	Parallel; 7 studies	127/127	0.39 (0.06 to 0.72)	0.019	39%	⊕⊕⊕⊖ Moderate
	Crossover; 4 studies	88/88	0.99 (–0.50 to 2.49)	0.194	94%	⊕⊖⊖⊖ Very Low
	by study interventions					
	Balance specific; 2 studies	45/45	0.89 (0.46 to 1.33)	<0.001	0%	⊕⊖⊖⊖ Very Low
	Not specific; 9 studies	170/170	0.52 (–0.16 to 1.20)	0.133	86%	⊕⊕⊖⊖ Low

Meta-regression

- age = B=0.01, 95% CI –0.10 to 0.13; p=0.81; R^2=0%; I^2=85%
- treatment duration (weeks) = B=0.04, 95% CI –0.09 to 0.16; p=0.53; R^2=0%; I^2=85%

- Comparison Intervention x Control on Balance for AP postural stability

Subgroup analysis: studies that applied passive interventions

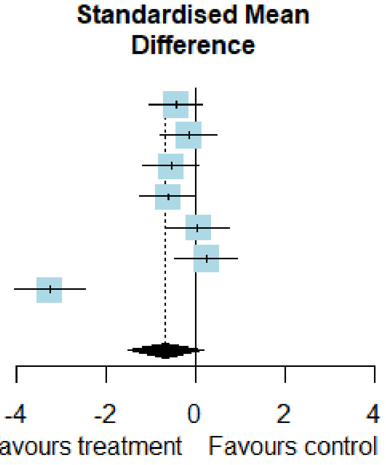

Study	Experimental Total	Experimental Mean	Experimental SD	Control Total	Control Mean	Control SD	SMD	95%-CI	Weight
Motealleh et al. [108]	22	-69.22	7.39	22	-65.81	7.85	-0.44	[-1.04; 0.16]	14.5%
Ferreira et al. [109]	20	5.79	2.10	20	6.09	1.62	-0.16	[-0.78; 0.46]	14.5%
Aminaka et al. [30]	20	-63.50	1.30	20	-62.80	1.20	-0.55	[-1.18; 0.08]	14.4%
Zarei et al. [110]	20	-0.89	0.11	20	-0.81	0.14	-0.62	[-1.26; 0.01]	14.4%
Ahmadi et al. [111]	16	1.40	0.25	16	1.39	0.20	0.04	[-0.65; 0.74]	14.2%
Song et al. [94]	16	-66.15	4.64	16	-67.18	3.74	0.24	[-0.46; 0.93]	14.2%
Goel and Bhatia [112]	30	-0.73	0.03	30	-0.64	0.03	-3.26	[-4.05; -2.47]	13.8%
**Random effects model**	**144**			**144**			**-0.67**	**[-1.52; 0.19]**	**100.0%**

Heterogeneity: $I^2 = 89\%$, $\tau^2 = 1.2052$, $p < 0.01$

- Comparison Intervention x Control on Balance for AP postural stability

Subgroup analysis: studies that applied exercise as intervention

Study	Experimental Total	Experimental Mean	Experimental SD	Control Total	Control Mean	Control SD	SMD	95%-CI	Weight
Steinberg et al. [107]	28	-59.80	5.50	29	-54.80	5.30	-0.91	[-1.46; -0.37]	30.4%
Foroughi et al. [28]	17	0.35	0.11	16	0.48	0.18	-0.86	[-1.57; -0.14]	24.3%
Motealleh et al. [62]	14	-0.70	0.11	14	-0.71	0.17	0.07	[-0.67; 0.81]	23.5%
Ebrahimi et al. [113]	12	-0.71	0.12	12	-0.70	0.12	-0.06	[-0.87; 0.74]	21.7%
**Random effects model**	**71**			**71**			**-0.48**	**[-0.99; 0.03]**	**100.0%**

Heterogeneity: $I^2 = 54\%$, $\tau^2 = 0.1443$, $p = 0.09$

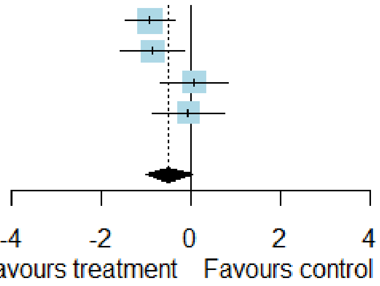

- Comparison Intervention x Control on Balance for AP postural stability

Subgroup analysis: studies that used no intervention/sham as control intervention

Study	Experimental Total	Experimental Mean	Experimental SD	Control Total	Control Mean	Control SD	SMD	95%-CI	Weight
Steinberg et al. [107]	28	-59.80	5.50	29	-54.80	5.30	-0.91	[-1.46; -0.37]	12.9%
Motealleh et al. [108]	22	-69.22	7.39	22	-65.81	7.85	-0.44	[-1.04; 0.16]	12.8%
Ferreira et al. [109]	20	5.79	2.10	20	6.09	1.62	-0.16	[-0.78; 0.46]	12.7%
Aminaka et al. [30]	20	-63.50	1.30	20	-62.80	1.20	-0.55	[-1.18; 0.08]	12.7%
Ahmadi et al. [111]	16	1.40	0.25	16	1.39	0.20	0.04	[-0.65; 0.74]	12.4%
Song et al. [94]	16	-66.15	4.64	16	-67.18	3.74	0.24	[-0.46; 0.93]	12.4%
Goel and Bhatia [112]	30	-0.73	0.03	30	-0.64	0.03	-3.26	[-4.05; -2.47]	12.1%
Ebrahimi et al. [113]	12	-0.71	0.12	12	-0.70	0.12	-0.06	[-0.87; 0.74]	12.0%
**Random effects model**	**164**			**165**			**-0.63**	**[-1.38; 0.12]**	**100.0%**

Heterogeneity: $I^2 = 88\%$, $\tau^2 = 1.0663$, $p < 0.01$

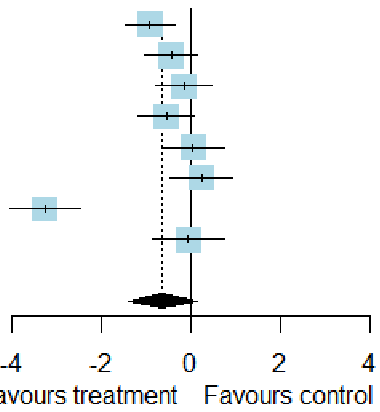

- Comparison Intervention x Control on Balance for AP postural stability

Subgroup analysis: studies that used exercise protocols as control intervention

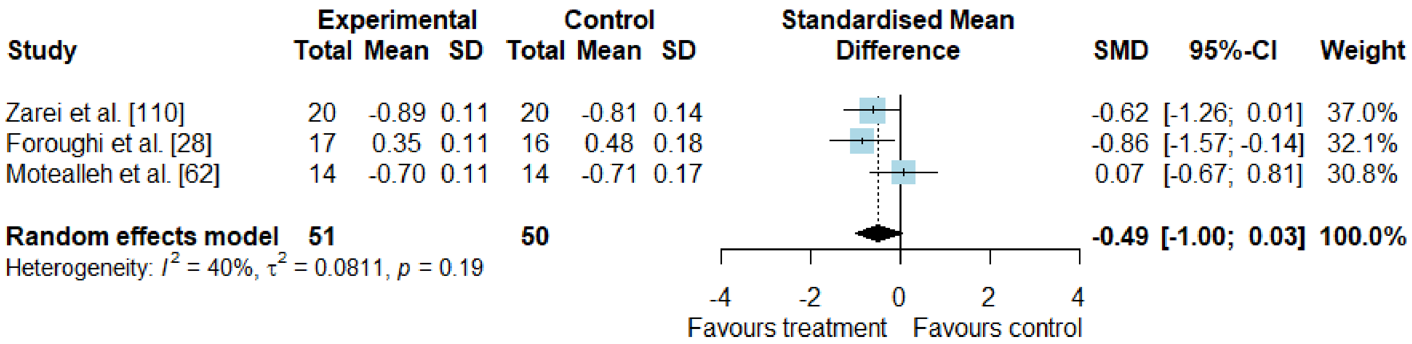

- Comparison Intervention x Control on Balance for AP postural stability

Subgroup analysis: parallel-controlled trials

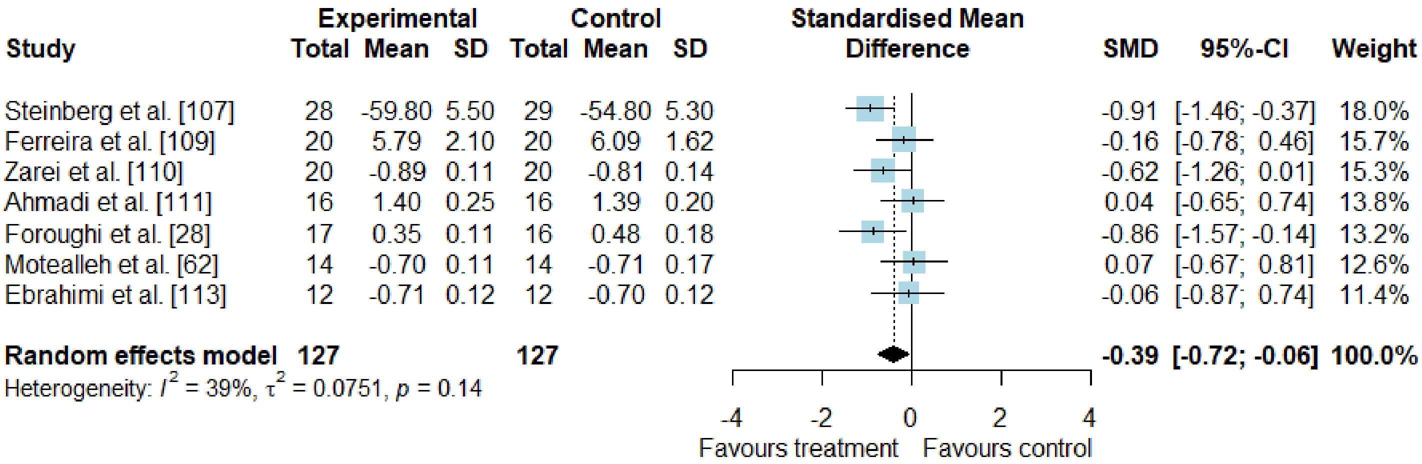

- Comparison Intervention x Control on Balance for AP postural stability

Subgroup analysis: crossover-controlled trials

Study	Experimental Total	Experimental Mean	Experimental SD	Control Total	Control Mean	Control SD	SMD	95%-CI	Weight
Motealleh et al. [108]	22	-69.22	7.39	22	-65.81	7.85	-0.44	[-1.04; 0.16]	25.3%
Aminaka et al. [30]	20	-63.50	1.30	20	-62.80	1.20	-0.55	[-1.18; 0.08]	25.2%
Song et al. [94]	16	-66.15	4.64	16	-67.18	3.74	0.24	[-0.46; 0.93]	24.9%
Goel and Bhatia [112]	30	-0.73	0.03	30	-0.64	0.03	-3.26	[-4.05; -2.47]	24.6%
**Random effects model**	**88**			**88**			**-0.99**	**[-2.49; 0.50]**	**100.0%**

Heterogeneity: $I^2 = 94\%$, $\tau^2 = 2.2086$, $p < 0.01$

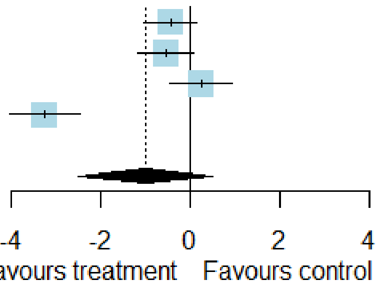

- Comparison Intervention x Control on Balance for AP postural stability

Subgroup analysis: studies aiming to evaluate the effects of balance exercises

Study	Experimental Total	Experimental Mean	Experimental SD	Control Total	Control Mean	Control SD	SMD	95%-CI	Weight
Steinberg et al. [107]	28	-59.80	5.50	29	-54.80	5.30	-0.91	[-1.46; -0.37]	63.2%
Foroughi et al. [28]	17	0.35	0.11	16	0.48	0.18	-0.86	[-1.57; -0.14]	36.8%
**Random effects model**	**45**			**45**			**-0.89**	**[-1.33; -0.46]**	**100.0%**

Heterogeneity: $I^2 = 0\%$, $\tau^2 = 0$, $p = 0.90$

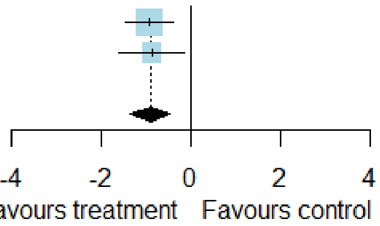

- Comparison Intervention x Control on Balance for AP postural stability

Subgroup analysis: studies aiming to evaluate the effects of intervention not related to balance exercises

Study	Experimental			Control			SMD	95%-CI	Weight
	Total	Mean	SD	Total	Mean	SD			
Motealleh et al. [108]	22	-69.22	7.39	22	-65.81	7.85	-0.44	[-1.04; 0.16]	11.4%
Ferreira et al. [109]	20	5.79	2.10	20	6.09	1.62	-0.16	[-0.78; 0.46]	11.4%
Aminaka et al. [30]	20	-63.50	1.30	20	-62.80	1.20	-0.55	[-1.18; 0.08]	11.3%
Zarei et al. [110]	20	-0.89	0.11	20	-0.81	0.14	-0.62	[-1.26; 0.01]	11.3%
Ahmadi et al. [111]	16	1.40	0.25	16	1.39	0.20	0.04	[-0.65; 0.74]	11.1%
Song et al. [94]	16	-66.15	4.64	16	-67.18	3.74	0.24	[-0.46; 0.93]	11.1%
Motealleh et al. [62]	14	-0.70	0.11	14	-0.71	0.17	0.07	[-0.67; 0.81]	10.9%
Goel and Bhatia [112]	30	-0.73	0.03	30	-0.64	0.03	-3.26	[-4.05; -2.47]	10.7%
Ebrahimi et al. [113]	12	-0.71	0.12	12	-0.70	0.12	-0.06	[-0.87; 0.74]	10.7%
**Random effects model**	**170**			**170**			**-0.52**	**[-1.20; 0.16]**	**100.0%**

Heterogeneity: $I^2 = 86\%$, $\tau^2 = 0.9494$, $p < 0.01$

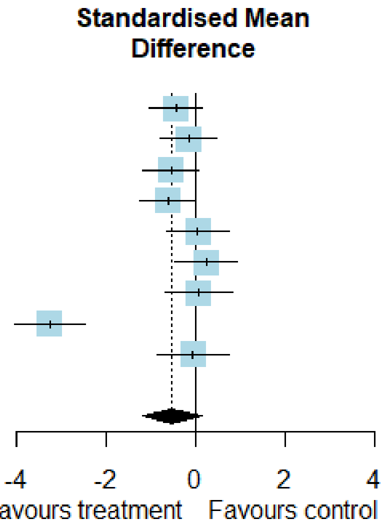

Additional file 7C. Results for Subgroup and Meta-regression analyses for Q3 (Balance exercise for pain and function)

Analysis		n Exp/Con	SMD (95% CI)	p-value	I²	Level of Evidence
Pain; 14 studies		439/398	0.82 (0.30 to 1.33)	0.002	89%	⊕⊕⊖⊖ Low
Subgroup analyses	***by study aim***					
	Balance specific; 4 studies	91/91	1.47 (0.40 to 2.55)	0.007	88%	⊕⊖⊖⊖ Very Low
	Not specific; 10 studies	348/307	0.57 (0.01 to 1.11)	0.047	88%	
	by comparator					
	Minimal interventions; 9 studies	248/232	1.02 (0.35 to 1.69)	0.003	89%	⊕⊕⊖⊖ Low
	Exercise; 5 studies	191/166	0.46 (–0.35 to 1.28)	0.266	88%	⊕⊕⊖⊖ Low
	by interventions					
	Balance only; 2 studies	44/45	1.58 (–1.11 to 4.26)	0.249	95%	⊕⊖⊖⊖ Very Low
	Balance + exercise; 12 studies	395/353	0.71 (0.21 to 1.21)	0.006	88%	⊕⊕⊕⊖ Moderate
Meta-regression<br>- age = B=0.05, 95% CI –0.06 to 0.15; p=0.42; R^2=0%; I^2=89%<br>- treatment duration (weeks) = B=0.07, 95% CI –0.05 to 0.19; p=0.26; R^2=0%; I^2=89%						
Function; 10 studies		333/291	0.45 (0.13 to 0.78)	0.006	68%	⊕⊕⊕⊖ Moderate
Subgroup analyses	***by study aim***					
	Balance specific; 1 study	Not applicable				
	Not specific; 9 studies	316/275	0.38 (0.06 to 0.71)	0.019	65%	⊕⊕⊕⊖ Moderate
	by comparator					
	Minimal interventions; 6 studies	172/155	0.51 (0.03 to 0.99)	0.036	73%	⊕⊕⊕⊖ Moderate
	Exercise; 4 studies	161/136	0.37 (–0.09 to 0.83)	0.114	62%	⊕⊕⊖⊖ Low
	by interventions					
	Balance only; no study	Not applicable				
	Balance + exercise; 10 studies					
Meta-regression<br>- age = B=–0.05, 95% CI –0.20 to 0.08; p=0.40; R^2=0%; I^2=68%<br>- treatment duration (weeks) = B=–0.04, 95% CI –0.12 to 0.05; p=0.40; R^2=0%; I^2=70%						

- Comparison Balance x No balance interventions on Pain

Subgroup analysis: studies aiming to evaluate the effects of balance exercises

Study	Experimental Total	Experimental Mean	Experimental SD	Control Total	Control Mean	Control SD	SMD	95%-CI	Weight
Steinberg et al. [107]	28	4.45	2.18	29	5.00	2.23	-0.25	[-0.77; 0.28]	26.6%
Mahmoud and Kamel [114]	30	1.90	0.90	30	3.50	1.50	-1.28	[-1.84; -0.72]	26.4%
Foroughi et al. [28]	17	1.58	0.93	16	3.31	1.13	-1.64	[-2.44; -0.83]	24.5%
Yalfani et al. [128]	16	2.66	1.17	16	6.53	1.35	-2.99	[-4.03; -1.94]	22.4%
**Random effects model**	**91**			**91**			**-1.47**	**[-2.55; -0.40]**	**100.0%**

Heterogeneity: $I^2 = 88\%$, $\tau^2 = 1.0644$, $p < 0.01$

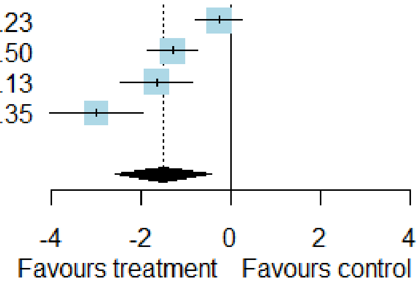

- Comparison Balance x No balance interventions on Pain

Subgroup analysis: studies aiming to evaluate the effects of exercise in general

Study	Experimental Total	Experimental Mean	Experimental SD	Control Total	Control Mean	Control SD	SMD	95%-CI	Weight
Ferber et al. [122]	111	1.96	1.92	88	1.99	2.05	-0.02	[-0.29; 0.26]	11.1%
van Linschoten et al. [123]	65	3.81	2.90	66	4.60	3.00	-0.27	[-0.61; 0.08]	11.0%
Clark et al. [124]	32	32.95	34.44	21	41.80	40.60	-0.24	[-0.79; 0.32]	10.3%
Emamvirdi et al. [125]	32	3.10	1.61	32	6.10	1.12	-2.14	[-2.76; -1.52]	10.0%
Boitrago et al. [61]	30	1.90	1.80	30	6.07	2.20	-2.05	[-2.68; -1.42]	10.0%
Rabelo et al. [32]	16	2.00	1.70	17	2.20	1.60	-0.12	[-0.80; 0.57]	9.8%
Shadloo et al. [126]	15	2.00	1.54	15	2.00	1.66	0.00	[-0.72; 0.72]	9.6%
Molgaard et al. [127]	17	78.00	11.00	15	69.00	15.00	0.67	[-0.04; 1.39]	9.6%
Loudon et al. [31]	18	21.70	16.31	11	34.80	17.70	-0.76	[-1.53; 0.02]	9.4%
Ebrahimi et al. [113]	12	1.71	1.38	12	3.07	1.82	-0.81	[-1.65; 0.03]	9.1%
**Random effects model**	**348**			**307**			**-0.57**	**[-1.12; -0.01]**	**100.0%**

Heterogeneity: $I^2 = 88\%$, $\tau^2 = 0.7058$, $p < 0.01$

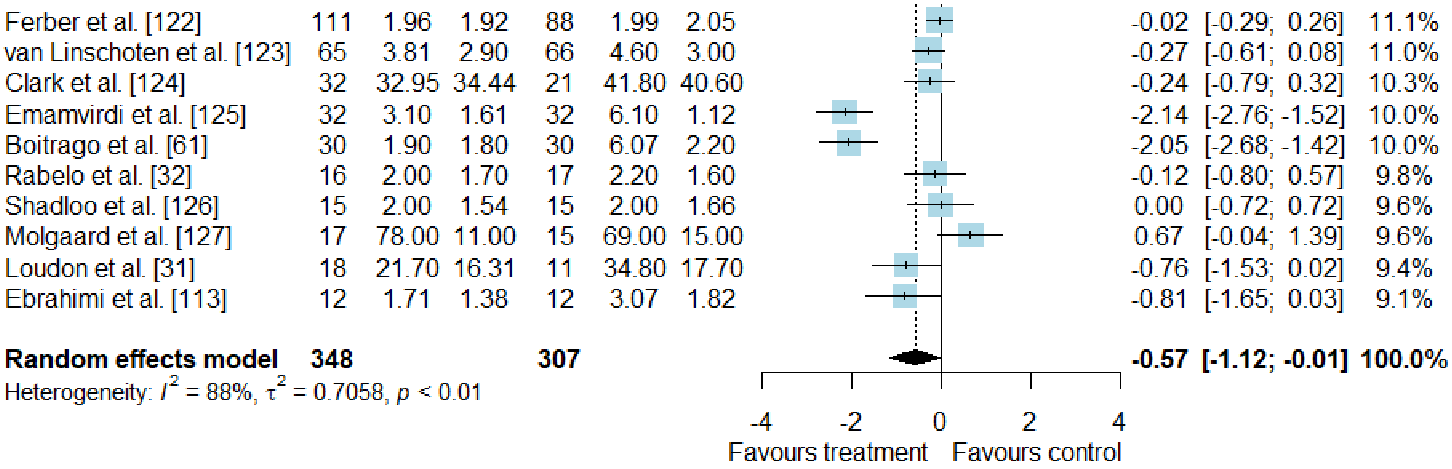

- Comparison Balance x No balance interventions on Pain

Subgroup analysis: control interventions were minimal intervention protocols

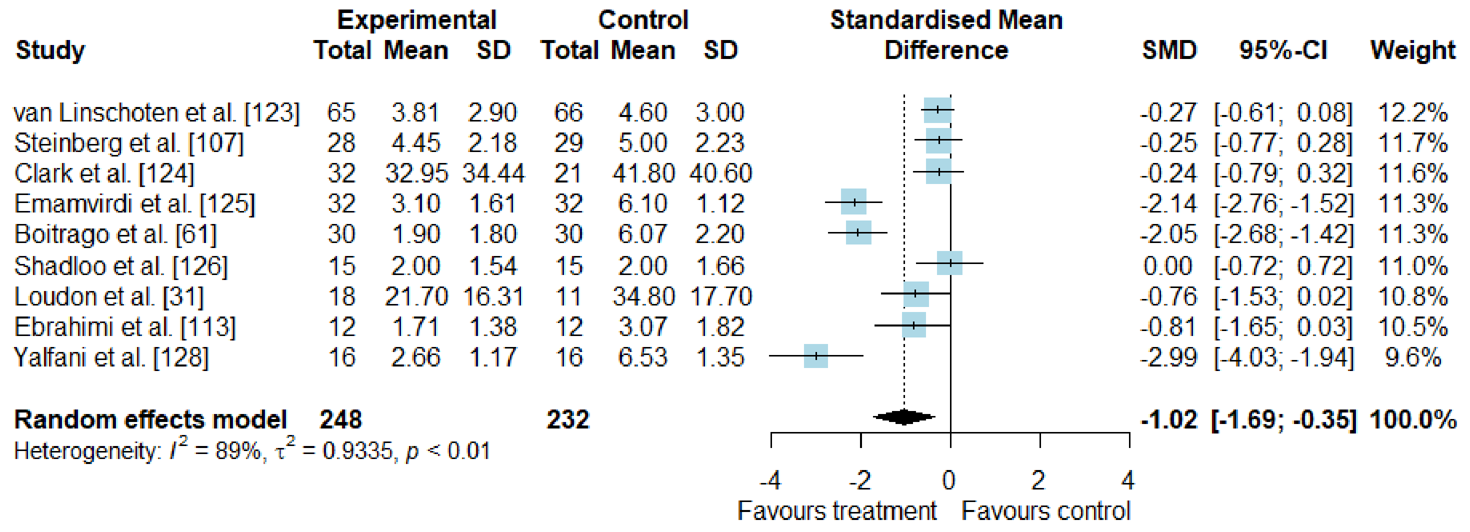

- Comparison Balance x No balance interventions on Pain

Subgroup analysis: control intervention were exercise protocols

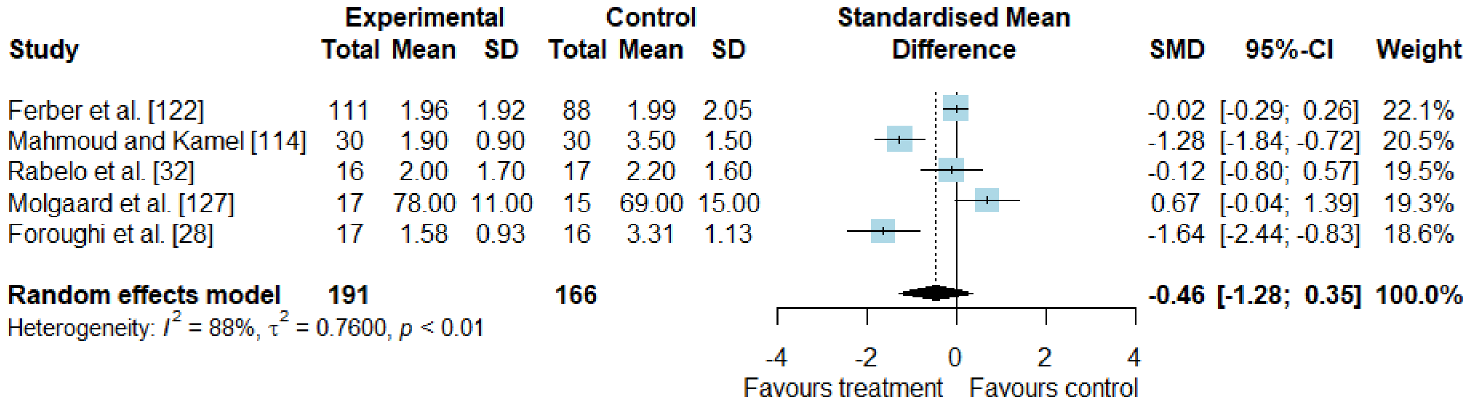

- Comparison Balance x No balance interventions on Pain

Subgroup analysis: experimental interventions were only balance exercises

Study	Experimental Total	Experimental Mean	Experimental SD	Control Total	Control Mean	Control SD	SMD	95%-CI	Weight
Steinberg et al. [107]	28	4.45	2.18	29	5.00	2.23	-0.25	[-0.77; 0.28]	51.4%
Yalfani et al. [128]	16	2.66	1.17	16	6.53	1.35	-2.99	[-4.03; -1.94]	48.6%
**Random effects model**	**44**			**45**			**-1.58**	**[-4.26; 1.11]**	**100.0%**

Heterogeneity: $I^2 = 95\%$, $\tau^2 = 3.5779$, $p < 0.01$

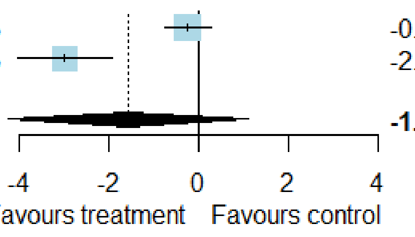

- Comparison Balance x No balance interventions on Pain

Subgroup analysis: experimental interventions were only exercise protocols

Study	Experimental Total	Experimental Mean	Experimental SD	Control Total	Control Mean	Control SD	SMD	95%-CI	Weight
Ferber et al. [122]	111	1.96	1.92	88	1.99	2.05	-0.02	[-0.29; 0.26]	9.4%
van Linschoten et al. [123]	65	3.81	2.90	66	4.60	3.00	-0.27	[-0.61; 0.08]	9.2%
Clark et al. [124]	32	32.95	34.44	21	41.80	40.60	-0.24	[-0.79; 0.32]	8.6%
Mahmoud and Kamel [114]	30	1.90	0.90	30	3.50	1.50	-1.28	[-1.84; -0.72]	8.6%
Emamvirdi et al. [125]	32	3.10	1.61	32	6.10	1.12	-2.14	[-2.76; -1.52]	8.4%
Boitrago et al. [61]	30	1.90	1.80	30	6.07	2.20	-2.05	[-2.68; -1.42]	8.4%
Rabelo et al. [32]	16	2.00	1.70	17	2.20	1.60	-0.12	[-0.80; 0.57]	8.2%
Shadloo et al. [126]	15	2.00	1.54	15	2.00	1.66	0.00	[-0.72; 0.72]	8.1%
Molgaard et al. [127]	17	78.00	11.00	15	69.00	15.00	0.67	[-0.04; 1.39]	8.1%
Loudon et al. [31]	18	21.70	16.31	11	34.80	17.70	-0.76	[-1.53; 0.02]	7.8%
Foroughi et al. [28]	17	1.58	0.93	16	3.31	1.13	-1.64	[-2.44; -0.83]	7.7%
Ebrahimi et al. [113]	12	1.71	1.38	12	3.07	1.82	-0.81	[-1.65; 0.03]	7.6%
**Random effects model**	**395**			**353**			**-0.71**	**[-1.21; -0.21]**	**100.0%**

Heterogeneity: $I^2 = 88\%$, $\tau^2 = 0.6800$, $p < 0.01$

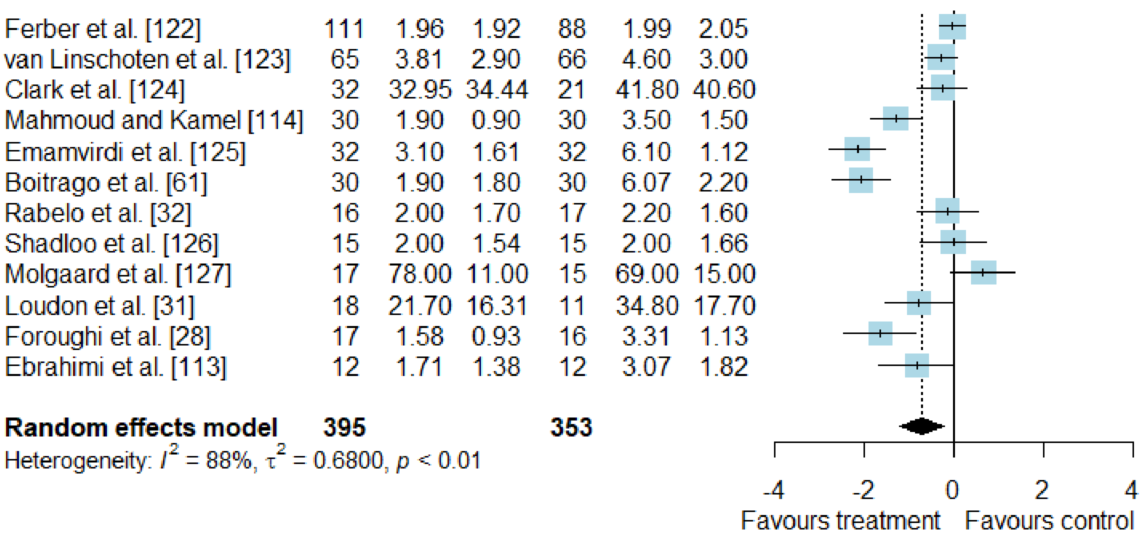

- Comparison Balance x No balance interventions on Function (PROMs)

Subgroup analysis: studies aiming to evaluate the effects of balance exercises

NOT APPLICABLE

- Comparison Balance x No balance interventions on Function (PROMs)

Subgroup analysis: studies aiming to evaluate the effects of exercise in general

Study	Experimental Total	Experimental Mean	Experimental SD	Control Total	Control Mean	Control SD	SMD	95%-CI	Weight
Ferber et al. [122]	111	87.95	11.26	88	87.67	10.53	0.03	[-0.25; 0.31]	15.9%
van Linschoten et al. [123]	65	78.80	15.50	66	74.90	17.60	0.23	[-0.11; 0.58]	14.9%
Boitrago et al. [61]	30	85.80	12.30	30	71.83	11.80	1.14	[0.60; 1.69]	11.8%
Clark et al. [124]	32	10.75	11.01	21	13.80	15.80	-0.23	[-0.78; 0.32]	11.8%
Rabelo et al. [32]	16	85.80	9.20	17	83.70	8.30	0.23	[-0.45; 0.92]	9.9%
Molgaard et al. [127]	17	83.00	10.00	15	78.00	15.00	0.39	[-0.31; 1.09]	9.7%
Shadloo et al. [126]	15	88.66	3.01	15	88.06	3.12	0.19	[-0.53; 0.91]	9.5%
Ebrahimi et al. [113]	12	88.36	6.03	12	83.00	10.46	0.61	[-0.22; 1.43]	8.3%
Loudon et al. [31]	18	85.45	9.56	11	71.20	11.80	1.33	[0.49; 2.16]	8.2%
**Random effects model**	**316**			**275**			**0.38**	**[0.06; 0.71]**	**100.0%**

Heterogeneity: $I^2 = 65\%$, $\tau^2 = 0.1500$, $p < 0.01$

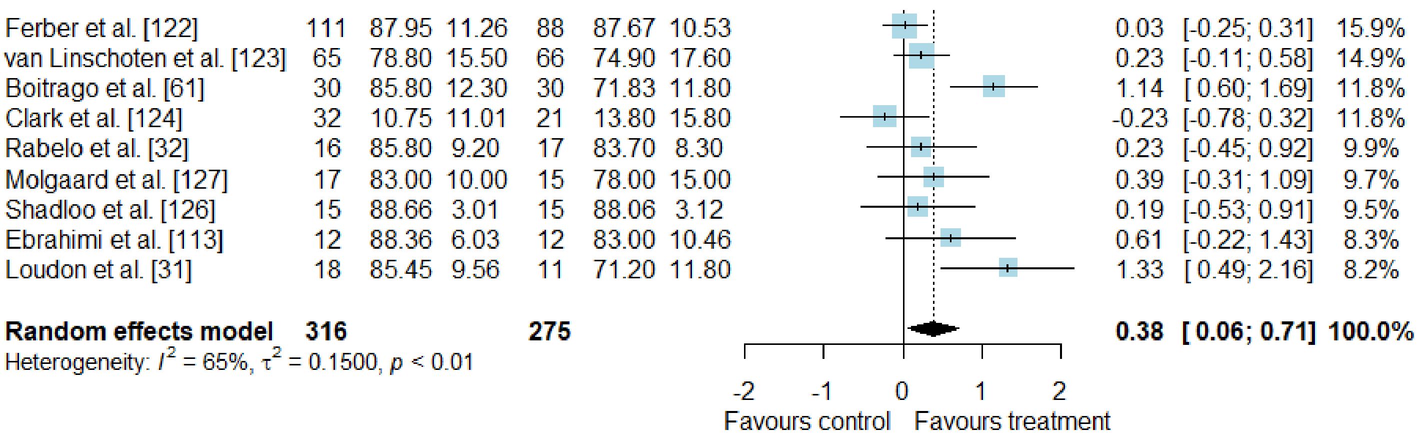

- Comparison Balance x No balance interventions on Function (PROMs)

Subgroup analysis: control interventions were minimal intervention protocols

Study	Experimental Total	Experimental Mean	Experimental SD	Control Total	Control Mean	Control SD	SMD	95%-CI	Weight
van Linschoten et al. [123]	65	78.80	15.50	66	74.90	17.60	0.23	[-0.11; 0.58]	21.0%
Boitrago et al. [61]	30	85.80	12.30	30	71.83	11.80	1.14	[0.60; 1.69]	18.0%
Clark et al. [124]	32	10.75	11.01	21	13.80	15.80	-0.23	[-0.78; 0.32]	17.9%
Shadloo et al. [126]	15	88.66	3.01	15	88.06	3.12	0.19	[-0.53; 0.91]	15.4%
Ebrahimi et al. [113]	12	88.36	6.03	12	83.00	10.46	0.61	[-0.22; 1.43]	13.9%
Loudon et al. [31]	18	85.45	9.56	11	71.20	11.80	1.33	[0.49; 2.16]	13.7%
**Random effects model**	**172**			**155**			**0.51**	**[0.03; 0.99]**	**100.0%**

Heterogeneity: $I^2 = 73\%$, $\tau^2 = 0.2507$, $p < 0.01$

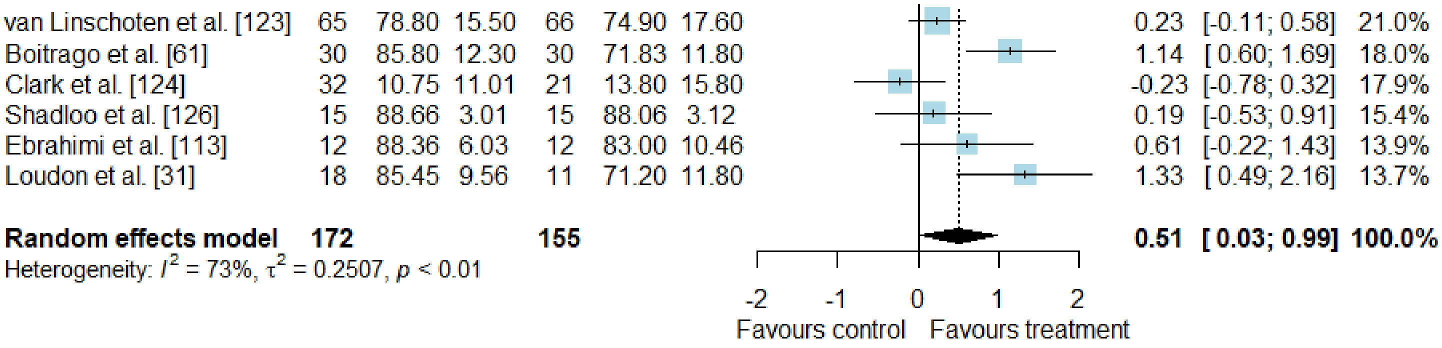

- Comparison Balance x No balance interventions on Function (PROMs)

Subgroup analysis: control intervention were exercise protocols

Study	Experimental Total	Experimental Mean	Experimental SD	Control Total	Control Mean	Control SD	SMD	95%-CI	Weight
Ferber et al. [122]	111	87.95	11.26	88	87.67	10.53	0.03	[-0.25; 0.31]	36.6%
Rabelo et al. [32]	16	85.80	9.20	17	83.70	8.30	0.23	[-0.45; 0.92]	21.9%
Molgaard et al. [127]	17	83.00	10.00	15	78.00	15.00	0.39	[-0.31; 1.09]	21.4%
Foroughi et al. [28]	17	88.05	4.75	16	81.93	5.79	1.13	[0.39; 1.87]	20.2%
**Random effects model**	**161**			**136**			**0.37**	**[-0.09; 0.83]**	**100.0%**

Heterogeneity: $I^2 = 62\%$, $\tau^2 = 0.1307$, $p = 0.05$

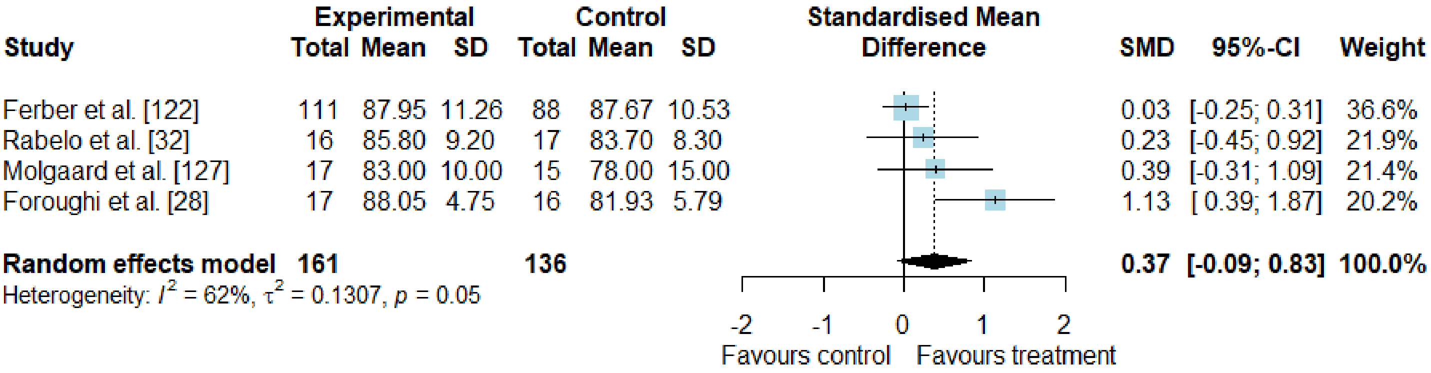

- Comparison Balance x No balance interventions on Function (PROMs)

Subgroup analysis: experimental interventions were only balance exercises

NOT APPLICABLE

- Comparison Balance x No balance interventions on Function (PROMs)

Subgroup analysis: experimental interventions were only exercise protocols

NOT APPLICABLE
