# Supplementary material for: Is Postural Control Affected in People with Patellofemoral Pain and Should it be Part of Rehabilitation? A Systematic Review with Meta-analysis
Source: Sports Med Open. 2022 Dec 12;8:144. doi: 10.1186/s40798-022-00538-4 (PMC9742077; doi:10.1186/s40798-022-00538-4)
Supplement: Supplementary file 8 — Additional file 8. Data of the included studies. [file 40798_2022_538_MOESM8_ESM.pdf]

Additional file 8A. Individual data of the included studies for Q1 (mean±SD).

<table>
<tr><th>Study</th><th>Participants</th><th>Balance Test</th><th>Results</th></tr>
<tr><td colspan="4">Posturography</td></tr>
<tr><td>Akhbari et al. [89]</td><td>15 PFP, 15 controls</td><td>Single-legged stance for 20 seconds</td><td>
<table>
<tr><th></th><th></th><th></th><th>PFP</th><th>Control</th></tr>
<tr><td rowspan="6">Static</td><td rowspan="3">Eyes open</td><td>Overall Stability Index</td><td>2.1 (1.1)</td><td>1.5 (0.5)</td></tr>
<tr><td>Anterior/Posterior Index</td><td>1.6 (1.1)</td><td>1.3 (0.9)</td></tr>
<tr><td>Medial/Lateral Index</td><td>1.0 (0.6)</td><td>0.9 (0.4)</td></tr>
<tr><td rowspan="3">Eyes closed</td><td>Overall Stability Index</td><td>4.4 (1.9)</td><td>4.1 (1.6)</td></tr>
<tr><td>Anterior/Posterior Index</td><td>3.6 (2.0)</td><td>3.2 (0.8)</td></tr>
<tr><td>Medial/Lateral Index</td><td>1.9 (0.8)</td><td>1.6 (0.7)</td></tr>
</table>
<table>
<tr><th></th><th></th><th></th><th>PFP</th><th>Control</th></tr>
<tr><td rowspan="6">Dynamic</td><td rowspan="3">Eyes open</td><td>Overall Stability Index</td><td>2.7 (1.3)</td><td>2.2 (0.9)</td></tr>
<tr><td>Anterior/Posterior Index</td><td>1.9 (1.2)</td><td>1.6 (1.1)</td></tr>
<tr><td>Medial/Lateral Index</td><td>1.7 (0.7)</td><td>1.3 (0.6)</td></tr>
<tr><td rowspan="3">Eyes closed</td><td>Overall Stability Index</td><td>5.7 (1.7)</td><td>5.2 (1.5)</td></tr>
<tr><td>Anterior/Posterior Index</td><td>4.6 (1.9)</td><td>4.1 (1.7)</td></tr>
<tr><td>Medial/Lateral Index</td><td>2.6 (0.9)</td><td>2.3 (0.8)</td></tr>
</table>
</td></tr>
<tr><td>Ibrahim et al. [86]</td><td>30 PFP, 30 controls</td><td>Single and double-legged stance for 20 seconds</td><td>
<table>
<tr><th>Dynamic</th><th>PFP</th><th>Control</th></tr>
<tr><td>Overall Stability Index</td><td>4.9 (3.1)</td><td>2.2 (0.7)</td></tr>
<tr><td>Anterior/Posterior Index</td><td>3.3 (2.3)</td><td>1.3 (0.3)</td></tr>
<tr><td>Medial/Lateral Index</td><td>3.0 (1.8)</td><td>1.5 (0.6)</td></tr>
</table>
</td></tr>
<tr><td>Kim et al. [99]</td><td>19 PFP, 19 controls</td><td>Single-legged drop landing</td><td>
<table>
<tr><th></th><th>PFP</th><th>Control</th></tr>
<tr><td>Anteroposterior stability index</td><td>0.007 (0.002)</td><td>0.005 (0.001)</td></tr>
<tr><td>Mediolateral stability index</td><td>0.012 (0.002)</td><td>0.013 (0.002)</td></tr>
<tr><td>Vertical stability index</td><td>0.017 (0.009)</td><td>0.019 (0.010)</td></tr>
<tr><td>Dynamic postural (Overall) stability index</td><td>0.035 (0.011)</td><td>0.037 (0.010)</td></tr>
</table>
</td></tr>
<tr><td>Negahban et al. [90]</td><td>15 PFP, 15 controls</td><td>Single-legged stance for 20 seconds (open eyes)</td><td>
<table>
<tr><th>Dynamic</th><th>PFP</th><th>Control</th></tr>
<tr><td>Overall Stability Index</td><td>4.54 (1.77)</td><td>3.63 (1.35)</td></tr>
<tr><td>Anterior/Posterior Index</td><td>2.83 (1.47)</td><td>2.12 (0.88)</td></tr>
<tr><td>Medial/Lateral Index</td><td>3.07 (1.30)</td><td>2.45 (1.45)</td></tr>
</table>
</td></tr>
</table>

<table>
<tr><td>Yelvar et al. [102]</td><td>22 PFP, 22 controls</td><td>Posturographic assessment using a Tetrax system</td><td>
<table>
<tr><th></th><th></th><th></th><th>PFP</th><th>Control</th></tr>
<tr><td rowspan="4">Fourier spectral intensity</td><td rowspan="2">Standing straight</td><td>Eyes open</td><td>1.97 (0.66)</td><td>1.56 (0.65)</td></tr>
<tr><td>Eyes closed</td><td>3.02 (1.46)</td><td>2.24 (1.11)</td></tr>
<tr><td rowspan="2">On pillows</td><td>Eyes open</td><td>2.94 (1.15)</td><td>2.28 (1.16)</td></tr>
<tr><td>Eyes closed</td><td>4.51 (1.45)</td><td>2.92 (0.98)</td></tr>
<tr><td rowspan="4">Stability Index</td><td rowspan="2">Standing straight</td><td>Eyes open</td><td>12.16 (3.14)</td><td>11.09 (3.99)</td></tr>
<tr><td>Eyes closed</td><td>17.44 (5.51)</td><td>16.13 (6.24)</td></tr>
<tr><td rowspan="2">On pillows</td><td>Eyes open</td><td>15.48 (5.16)</td><td>13.62 (4.55)</td></tr>
<tr><td>Eyes closed</td><td>26.30 (5.00)</td><td>19.30 (4.66)</td></tr>
<tr><td rowspan="4">Weight Distribution Index</td><td rowspan="2">Standing straight</td><td>Eyes open</td><td>6.89 (2.56)</td><td>5.87 (2.17)</td></tr>
<tr><td>Eyes closed</td><td>6.04 (3.03)</td><td>5.25 (2.17)</td></tr>
<tr><td rowspan="2">On pillows</td><td>Eyes open</td><td>9.95 (3.15)</td><td>8.01 (4.08)</td></tr>
<tr><td>Eyes closed</td><td>8.16 (3.19)</td><td>7.25 (3.41)</td></tr>
</table>
</td></tr>
<tr><td colspan="4">Center of Pressure (CoP) Behaviour</td></tr>
<tr><td>Carry et al. [57]</td><td>7 PFP, 7 controls</td><td>Single-legged squat</td><td>
<table>
<tr><th></th><th>PFP</th><th>Control</th></tr>
<tr><td>CoP Mean distance (mm)</td><td>22.62 (3.84)</td><td>24.79 (2.13)</td></tr>
<tr><td>CoP Root-mean square (mm)</td><td>25.09 (4.38)</td><td>27.56 (2.28)</td></tr>
<tr><td>CoP range (mm)</td><td>78.87 (10.67)</td><td>86.36 (7.38)</td></tr>
<tr><td>CoP area (mm^2)</td><td>5459.89 (1627.74)</td><td>6131.39 (1240.87)</td></tr>
</table>
</td></tr>
<tr><td>Carvalho-e-Silva et al. [87]</td><td>25 PFP, 25 controls</td><td>Step task</td><td>
<table>
<tr><th></th><th>PFP</th><th>Control</th></tr>
<tr><td>CoP total displacement (cm)</td><td>888.7 (76.1)</td><td>812.9 (69.2)</td></tr>
<tr><td>CoP AP displacement (mm)</td><td>61.0 (5.9)</td><td>60.7 (6.0)</td></tr>
<tr><td>CoP ML displacement (mm)</td><td>32.3 (5.5)</td><td>21.7 (2.7)</td></tr>
<tr><td>CoP total velocity (m/s)</td><td>45.5 (2.9)</td><td>44.4 (2.1)</td></tr>
<tr><td>CoP AP velocity (m/s)</td><td>36.7 (2.9)</td><td>35.3 (1.8)</td></tr>
<tr><td>CoP ML velocity (m/s)</td><td>22.2 (2.0)</td><td>17.0 (1.6)</td></tr>
</table>
</td></tr>
</table>

<table>
<tr><td></td><td></td><td></td><td></td></tr>
<tr><td>Felicio et al. [88]</td><td>15 PFP, 15 controls</td><td>Single-legged stance for 30 seconds (closed eyes)</td><td>
<table>
<tr><th></th><th>PFP</th><th>Control</th></tr>
<tr><td>CoP AP displacement (cm)</td><td>5.00 (0.74)</td><td>4.90 (0.89)</td></tr>
<tr><td>CoP ML displacement (cm)</td><td>7.18 (2.09)</td><td>7.39 (2.40)</td></tr>
<tr><td>CoP area (cm²)</td><td>16.12 (4.66)</td><td>16.52 (4.43)</td></tr>
<tr><td>CoP AP velocity (cm/s)</td><td>6.68 (1.30)</td><td>5.90 (1.65)</td></tr>
<tr><td>CoP ML velocity (cm/s)</td><td>6.22 (1.49)</td><td>5.74 (1.90)</td></tr>
<tr><td>Frequency AP (Hz)</td><td>0.33 (0.31)</td><td>0.34 (0.24)</td></tr>
<tr><td>Frequency ML (Hz)</td><td>0.24 (0.21)</td><td>0.29 (0.21)</td></tr>
</table>
</td></tr>
<tr><td>Gwynne [58]</td><td>30 PFP, 30 controls</td><td>Single-legged squats</td><td>
<table>
<tr><th></th><th>PFP</th><th>Control</th></tr>
<tr><td>CoP ML index</td><td>1.50 (0.15)</td><td>1.43 (0.10)</td></tr>
<tr><td>CoP overall velocity (m/s)</td><td>2.14 (1.28)</td><td>1.45 (0.72)</td></tr>
<tr><td>CoP ML velocity (m/s)</td><td>0.49 (0.24)</td><td>0.47 (0.23)</td></tr>
<tr><td>CoP AP velocity (m/s)</td><td>2.03 (1.27)</td><td>1.38 (0.68)</td></tr>
</table>
</td></tr>
<tr><td>Lee et al. [29]</td><td>20 PFP, 19 controls</td><td>Step-down task</td><td>
<table>
<tr><th></th><th>PFP</th><th>Control</th></tr>
<tr><td>CoP ML peak displacement (mm)</td><td>39.8 (6.7)</td><td>24.3 (3.8)</td></tr>
<tr><td>CoP ML mean displacement (mm)</td><td>24.7 (16.3)</td><td>13.5 (4.4)</td></tr>
<tr><td>CoP ML variability (mm)</td><td>15.3 (8.4)</td><td>9.2 (3.9)</td></tr>
</table>
</td></tr>
<tr><td>Manojlovic et al. [100]</td><td>18 PFP, 37 controls</td><td>Single-legged stance for 30 seconds (open eyes)</td><td>
<table>
<tr><th></th><th>PFP</th><th>Control</th></tr>
<tr><td>CoP AP displacement (mm)</td><td>5.0 (1.6)</td><td>5.6 (1.5)</td></tr>
<tr><td>CoP ML displacement (mm)</td><td>5.9 (2.2)</td><td>6.6 (2.4)</td></tr>
<tr><td>CoP AP velocity (mm/s)</td><td>23.2 (4.9)</td><td>25.8 (6.4)</td></tr>
<tr><td>CoP ML velocity (mm/s)</td><td>25.1 (5.5)</td><td>26.8 (6.7)</td></tr>
<tr><td>Standing asymmetry index (%)</td><td>3.6 (3.4)</td><td>3.2 (2.6)</td></tr>
<tr><td>Semi-squat asymmetry index (%)</td><td>4.7 (2.1)</td><td>3.9 (2.3)</td></tr>
</table>
</td></tr>
<tr><td>Motealleh et al. [92]</td><td>21 PFP, 21 controls</td><td>Seated balance for 7 seconds (open eyes)</td><td>
<table>
<tr><th></th><th>PFP</th><th>Control</th></tr>
<tr><td>CoP velocity (mm/s)</td><td>51.18 (22.36)</td><td>26.72 (5.08)</td></tr>
<tr><td>CoP AP displacement (mm)</td><td>26.04 (11.48)</td><td>9.35 (4.02)</td></tr>
<tr><td>CoP ML displacement (mm)</td><td>25.47 (12.40)</td><td>9.70 (3.70)</td></tr>
<tr><td>CoP area (mm²)</td><td>564.60 (263.70)</td><td>186.38 (68.04)</td></tr>
</table>
</td></tr>
</table>

<table>
<tr><td>Nasab et al. [91]</td><td>15 PFP, 15 controls</td><td>Double-legged stance for 60 seconds (open eyes)</td><td>
<table>
<tr><th></th><th>PFP</th><th>Control</th></tr>
<tr><td>CoP AP displacement (mm)</td><td>0.22 (0.14)</td><td>0.15 (0.09)</td></tr>
<tr><td>CoP ML displacement (mm)</td><td>0.34 (0.21)</td><td>0.14 (0.05)</td></tr>
<tr><td>CoP AP sum of displacement (mm)</td><td>562.14 (155.14)</td><td>502.24 (113.63)</td></tr>
<tr><td>CoP ML sum of displacement (mm)</td><td>699.29 (219.19)</td><td>567.26 (124.67)</td></tr>
<tr><td>CoP AP velocity (mm/s)</td><td>1124.3 (310.29)</td><td>1004.5 (227.25)</td></tr>
<tr><td>CoP ML velocity (mm/s)</td><td>1398.6 (438.38)</td><td>1202.4 (367.50)</td></tr>
</table>
</td></tr>
<tr><td>Naserpour et al. [103]</td><td>34 PFP, 34 controls</td><td>Step-down task</td><td>
<table>
<tr><th></th><th>PFP</th><th>Control</th></tr>
<tr><td>Time to CoP stabilization for AP direction (s)</td><td>9.09 (0.82)</td><td>8.43 (0.79)</td></tr>
<tr><td>Time to CoP stabilization for ML direction (s)</td><td>7.15 (2.11)</td><td>5.56 (1.95)</td></tr>
</table>
</td></tr>
<tr><td>Saad et al. [27]</td><td>15 PFP, 15 controls</td><td>Stair negotiation</td><td>
<table>
<tr><td rowspan="3">CoP área (cm^2)</td><th></th><th>PFP</th><th>Control</th></tr>
<tr><td>Step up</td><td>69.28 (33.36)</td><td>42.96 (10.22)</td></tr>
<tr><td>Step down</td><td>56.63 (24.56)</td><td>36.34 (10.92)</td></tr>
</table>
</td></tr>
<tr><td>Silva et al. [26]</td><td>29 PFP, 25 controls</td><td>Stair climbing</td><td>
<table>
<tr><th></th><th>PFP</th><th>Control</th></tr>
<tr><td>CoP area (cm^2)</td><td>26.4 (7.5)</td><td>43.1 (10.2)</td></tr>
</table>
</td></tr>
<tr><td>Stensdotter et al. [97]</td><td>17 PFP, 17 controls</td><td>Double-legged stance on sliding platform for 7 seconds (open eyes)</td><td>
<table>
<tr><th></th><th>PFP</th><th>Control</th></tr>
<tr><td>CoM AP displacement for anterior translation (cm)</td><td>3.10 (0.03)</td><td>2.90 (0.03)</td></tr>
<tr><td>CoM AP displacement for posterior translation (cm)</td><td colspan="2">not informed</td></tr>
<tr><td>CoM AP velocity for anterior translation (m/s)</td><td>0.21 (0.03)</td><td>0.21 (0.02)</td></tr>
<tr><td>CoM AP velocity for posterior translation (m/s)</td><td>0.20 (0.04)</td><td>0.20 (0.02)</td></tr>
</table>
</td></tr>
<tr><td>Stensdotter et al. [104]</td><td>17 PFP, 17 controls</td><td>Double-legged stance on sliding platform for 7 seconds (open eyes)</td><td>
<table>
<tr><th></th><th colspan="2">CoM Lateral displacement for anterior translation (mm)</th><th colspan="2">CoM Lateral displacement for posterior translation (mm)</th></tr>
<tr><th>Perturbation</th><th>PFP</th><th>Control</th><th>PFP</th><th>Control</th></tr>
<tr><td>Before</td><td colspan="4" rowspan="3">not informed</td></tr>
<tr><td>During</td></tr>
<tr><td>After</td></tr>
</table>
</td></tr>
<tr><td>Zamboti et al. [95]</td><td>10 PFP, 10 controls</td><td>Single-legged stance for 30 seconds (open eyes)</td><td>
<table>
<tr><th></th><th>PFP</th><th>Control</th></tr>
<tr><td>CoP área (cm^2)</td><td>7.93 (2.98)</td><td>6.41 (1.5)</td></tr>
<tr><td>CoP ML velocity (cm/s)</td><td>3.01 (0.9)</td><td>2.4 (0.53)</td></tr>
</table>
</td></tr>
</table>

<table>
<tr><td>Zeinalzadeh et al. [24]</td><td>28 PFP, 28 controls</td><td>Single-legged stance for 30 seconds</td><td>
<table>
<tr><th></th><th></th><th>PFP</th><th>Control</th></tr>
<tr><td rowspan="2">CoP area (cm²)</td><td>Eyes open</td><td>40.36 (19.29)</td><td>37.69 (28.44)</td></tr>
<tr><td>Eyes closed</td><td>131.67 (89.58)</td><td>82.37 (48.92)</td></tr>
<tr><td rowspan="2">CoP AP displacement (cm)</td><td>Eyes open</td><td>3.59 (0.97)</td><td>3.29 (1.17)</td></tr>
<tr><td>Eyes closed</td><td>15.52 (7.98)</td><td>8.87 (5.35)</td></tr>
<tr><td rowspan="2">CoP ML displacement (cm)</td><td>Eyes open</td><td>2.89 (1.06)</td><td>2.54 (0.55)</td></tr>
<tr><td>Eyes closed</td><td>13.97 (10.34)</td><td>7.02 (5.05)</td></tr>
<tr><td rowspan="2">CoP velocity (cm/s</td><td>Eyes open</td><td>0.65 (0.10)</td><td>0.45 (0.05)</td></tr>
<tr><td>Eyes closed</td><td>1.15 (0.55)</td><td>0.80 (0.40)</td></tr>
</table>
</td></tr>
<tr><td colspan="4">Star Excursion Balance Test (SEBT) and related tests</td></tr>
<tr><td>Aminaka et al. [30]</td><td>20 PFP, 20 controls</td><td>Anterior direction of SEBT</td><td>
<table>
<tr><th></th><th>PFP</th><th>Control</th></tr>
<tr><td>SEBT anterior (%)</td><td>62.8 (1.2)</td><td>65.6 (1.2)</td></tr>
</table>
</td></tr>
<tr><td>Arun et al. [96]</td><td>10 PFP, 10 controls</td><td>SEBT</td><td>
<table>
<tr><th>SEBT (inches)</th><th>PFP</th><th>Control</th></tr>
<tr><td>Anterior</td><td>48.5 (1.51)</td><td>53.9 (0.88)</td></tr>
<tr><td>Anteromedial</td><td>45.5 (1.51)</td><td>53.3 (1.49)</td></tr>
<tr><td>Anterolateral</td><td>43.0 (1.63)</td><td>52.5 (2.12)</td></tr>
<tr><td>Posterior</td><td>40.2 (1.87)</td><td>45.3 (1.77)</td></tr>
<tr><td>Posteromedial</td><td>35.4 (1.51)</td><td>46.9 (3.18)</td></tr>
<tr><td>Posterolateral</td><td>34.5 (2.46)</td><td>42.3 (1.16)</td></tr>
<tr><td>Medial</td><td>46.8 (1.55)</td><td>54.0 (1.33)</td></tr>
<tr><td>Lateral</td><td>46.1 (0.99)</td><td>52.7 (2.0)</td></tr>
</table>
</td></tr>
<tr><td>Coelho et al. [98]</td><td>48 PFP, 48 controls</td><td>Y-balance test</td><td>
<table>
<tr><th></th><th>PFP</th><th>Control</th></tr>
<tr><td>SEBT Anterior (%)</td><td>58.6 (6.6)</td><td>61.7 (5.9)</td></tr>
<tr><td>SEBT Posteromedial (%)</td><td>94.2 (13.2)</td><td>96.6 (9.3)</td></tr>
<tr><td>SEBT Posterolateral (%)</td><td>98.5 (12.6)</td><td>100.1 (8.6)</td></tr>
<tr><td>SEBT composite score</td><td>83.8 (9.3)</td><td>86.1 (6.5)</td></tr>
</table>
</td></tr>
<tr><td>Goto et al. [25]</td><td>14 PFP, 14 controls</td><td>Anterior direction of SEBT</td><td>
<table>
<tr><th></th><th>PFP</th><th>Control</th></tr>
<tr><td>SEBT anterior (%)</td><td>66.17 (4.99)</td><td>70.84 (4.39)</td></tr>
</table>
</td></tr>
<tr><td>Loudon et al. [93]</td><td>29 PFP, 11 controls</td><td>Anterior reach for 30 seconds “SEBT”</td><td>
<table>
<tr><th></th><th>PFP</th><th>Control</th></tr>
<tr><td>Balance and reach (rep)</td><td>17.93 (1.03)</td><td>16.65 (1.45)</td></tr>
<tr><td>Anterior reach (cm)</td><td>40.8 (9.1)</td><td>53.5 (6.7)</td></tr>
</table>
</td></tr>
<tr><td>Priore et al. [101]</td><td>55 PFP, 40 controls</td><td>SEBT</td><td>
<table>
<tr><th></th><th>PFP</th><th>Control</th></tr>
<tr><td>Mean of directions (%)</td><td>78 (8)</td><td>84 (8)</td></tr>
</table>
</td></tr>
</table>

<table>
<tr><td>Steinberg et al. [85]</td><td>83 PFP, 49 controls</td><td>Y-balance test</td><td>
<table>
<tr><th>Difference between limbs</th><th>PFP</th><th>Control</th></tr>
<tr><td>Anterior Y-test (cm)</td><td>4.1 (3.3)</td><td>2.4 (2.0)</td></tr>
<tr><td>Posteromedial Y-test (cm)</td><td>6.0 (4.3)</td><td>4.6 (3.9)</td></tr>
<tr><td>Posterolateral Y-test (cm)</td><td>6.2 (5.1)</td><td>5.4 (6.0)</td></tr>
</table>
</td></tr>
<tr><td>Song et al. [94]</td><td>16 PFP, 8 controls</td><td>Anterior direction of SEBT</td><td>
<table>
<tr><th></th><th>PFP</th><th>Control</th></tr>
<tr><td>SEBT anterior (%)</td><td>65.57 (4.83)</td><td>63.54 (8.10)</td></tr>
</table>
</td></tr>
<tr><td>Zamboti et al. [95]</td><td>10 PFP, 10 controls</td><td>SEBT</td><td>
<table>
<tr><th></th><th>PFP</th><th>Control</th></tr>
<tr><td>SEBT Anterior (%)</td><td>80.55 (4.72)</td><td>81.05 (4.58)</td></tr>
<tr><td>SEBT Posteromedial (%)</td><td>69.6 (9.94)</td><td>73.9 (6.94)</td></tr>
<tr><td>SEBT Posterolateral (%)</td><td>76.25 (8.81)</td><td>80.8 (6.05)</td></tr>
<tr><td>SEBT index</td><td>88.07 (7.68)</td><td>91.0 (5.78)</td></tr>
</table>
</td></tr>
</table>

Abbreviations: PFP = patellofemoral pain, CoP = centre of pressure, AP = anteroposterior, ML = mediolateral, CoM = centre of mass, SEBT = Star Excursion Balance Test

Additional file 8B. Individual data of the included studies for Q2 (mean±SD).

<table>
<tr><th>Study</th><th>Interventions</th><th>Balance Test</th><th>Results</th></tr>
<tr><td colspan="4">Posturography</td></tr>
<tr><td>Ahmadi et al. [111]</td><td>Neurofeedback training x No intervention</td><td>Double-legged stance for 30 seconds (closed eyes)</td><td>
<table>
<tr><td rowspan="2">Static</td><td colspan="2">Training (n=16)</td><td colspan="2">No intervention (n=16)</td></tr>
<tr><td>Pre</td><td>12 weeks</td><td>Pre</td><td>12 weeks</td></tr>
<tr><td>Anterior/Posterior index</td><td>1.46 (0.25)</td><td>1.40 (0.25)</td><td>1.34 (0.23)</td><td>1.39 (0.20)</td></tr>
<tr><td>Medial/Lateral index</td><td>1.66 (0.20)</td><td>1.68 (0.17)</td><td>1.41 (0.17)</td><td>1.52 (0.22)</td></tr>
<tr><td>Overall stability</td><td>1.51 (0.21)</td><td>1.49 (0.17)</td><td>1.30 (0.36)</td><td>1.46 (0.27)</td></tr>
</table>
</td></tr>
<tr><td>Aytar et al. [115]</td><td>Knee kinesiotaping (KT) x Sham tape</td><td>Posturographic assessment using Kinesthetic Ability Trainer (open eye)</td><td>
<table>
<tr><td rowspan="2"></td><td colspan="2">KT (n=12)</td><td colspan="2">Sham (n=10)</td></tr>
<tr><td>Pre</td><td>Post</td><td>Pre</td><td>Post</td></tr>
<tr><td>Static balance score</td><td>167.58 (80.05)</td><td>128.08 (52.44)</td><td>148.40 (62.92)</td><td>139.40 (63.62)</td></tr>
<tr><td>Dynamic balance score</td><td>2702.16 (1101.59)</td><td>2428.41 (959.76)</td><td>1912.30 (701.85)</td><td>1834.90 (674.40)</td></tr>
</table>
* Immediate effect</td></tr>
<tr><td>Mahmoud and Kamel [114]</td><td>Hip and balance exercise x Hip exercise</td><td>Single-legged stance for 30 seconds</td><td>
<table>
<tr><td rowspan="2"></td><td colspan="2">Balance (n=30)</td><td colspan="2">Control (n=30)</td></tr>
<tr><td>Pre</td><td>4 weeks</td><td>Pre</td><td>4 weeks</td></tr>
<tr><td>Overall stability index</td><td>14.51 (2.1)</td><td>11.38 (1.1)</td><td>15.17 (2.6)</td><td>14.76 (2.5)</td></tr>
</table>
</td></tr>
<tr><td colspan="4">Center of Pressure (CoP) Behaviour</td></tr>
<tr><td>Ferreira et al. [109]</td><td>McConnell patellar tape x Sham tape</td><td>Single-legged squat and stance for 30 seconds</td><td>
<table>
<tr><td rowspan="3"></td><td colspan="4">McConnell (n=20)</td><td colspan="4">Sham (n=20)</td></tr>
<tr><td colspan="2">Stance</td><td colspan="2">Squat</td><td colspan="2">Stance</td><td colspan="2">Squat</td></tr>
<tr><td>Pre</td><td>Post</td><td>Pre</td><td>Post</td><td>Pre</td><td>Post</td><td>Pre</td><td>Post</td></tr>
<tr><td>CoP area (cm^2)</td><td>6.83 (3.10)</td><td>6.81 (3.00)</td><td>13.34 (7.16)</td><td>13.69 (7.09)</td><td>6.99 (2.17)</td><td>6.90 (2.20)</td><td>14.35 (6.13)</td><td>14.06 (5.10)</td></tr>
<tr><td>CoP AP amplitude (cm)</td><td>3.77 (1.25)</td><td>3.63 (0.90)</td><td>5.69 (1.93)</td><td>5.79 (2.10)</td><td>3.57 (0.66)</td><td>3.62 (0.72)</td><td>6.04 (1.80)</td><td>6.09 (1.62)</td></tr>
<tr><td>CoP ML amplitude (cm)</td><td>2.79 (0.48)</td><td>2.65 (0.44)</td><td>3.36 (0.64)</td><td>3.25 (0.50)</td><td>2.86 (0.37)</td><td>2.77 (0.38)</td><td>3.39 (0.38)</td><td>3.25 (0.47)</td></tr>
<tr><td>CoP AP velocity (cm/s)</td><td>2.16 (0.60)</td><td>2.00 (0.48)</td><td>3.61 (0.88)</td><td>3.33 (0.74)</td><td>2.09 (0.44)</td><td>1.97 (0.47)</td><td>3.59 (0.70)</td><td>3.38 (0.66)</td></tr>
<tr><td>CoP ML velocity (cm/s)</td><td>2.50 (0.56)</td><td>2.27 (0.46)</td><td>3.37 (0.66)</td><td>2.95 (0.49)</td><td>2.43 (0.44)</td><td>2.26 (0.44)</td><td>3.29 (0.58)</td><td>3.09 (0.67)</td></tr>
<tr><td>AP Frequency (Hz)</td><td>0.54 (0.09)</td><td>0.52 (0.14)</td><td>0.67 (0.17)</td><td>0.57 (0.15)</td><td>0.54 (0.15)</td><td>0.52 (0.13)</td><td>0.61 (0.21)</td><td>0.53 (0.15)</td></tr>
<tr><td>ML Frequency (Hz)</td><td>0.78 (0.13)</td><td>0.74 (0.12)</td><td>0.90 (0.14)</td><td>0.80 (0.11)</td><td>0.73 (0.13)</td><td>0.69 (0.14)</td><td>0.83 (0.15)</td><td>0.81 (0.13)</td></tr>
</table>
* Immediate effect</td></tr>
</table>

<table>
<tr>
<td>Foroughi et al. [28]</td>
<td>Stretching, strengthening and postural control exercises x Stretching and strengthening exercises</td>
<td>CoP behaviour in seated position for 7 seconds</td>
<td>
<table>
<tr><td></td><td colspan="2">Experimental (n=17)</td><td colspan="2">Control (n=16)</td></tr>
<tr><td></td><td>Pre</td><td>After 4w</td><td>Pre</td><td>After 4w</td></tr>
<tr><td>CoP root-mean square AP distance (mm)</td><td>0.68 (0.27)</td><td>0.35 (0.11)</td><td>0.59 (0.22)</td><td>0.48 (0.18)</td></tr>
<tr><td>CoP root-mean square ML distance (mm)</td><td>1.48 (0.75)</td><td>0.62 (0.23)</td><td>1.07 (0.39)</td><td>0.67 (0.30)</td></tr>
<tr><td>CoP area (mm^2)</td><td>23.27 (19.76)</td><td>6.03 (3.69)</td><td>14.19 (7.96)</td><td>8.21 (7.27)</td></tr>
<tr><td>CoP velocity (mm/s)</td><td>67.78 (26.61)</td><td>35.54 (9.85)</td><td>49.93 (15.32)</td><td>40.53 (16.85)</td></tr>
</table>
</td>
</tr>
<tr>
<td>Lee et al. [29]</td>
<td>Hip brace x No brace</td>
<td>Step-down task</td>
<td>
<table>
<tr><td>n=20</td><td>Hip Brace</td><td>No Brace</td></tr>
<tr><td>CoP ML peak displacement (mm)</td><td>24.7 (4.7)</td><td>39.8 (6.7)</td></tr>
<tr><td>CoP ML mean displacement (mm)</td><td>16.8 (15.1)</td><td>24.7 (16.3)</td></tr>
<tr><td>CoP ML variability (mm)</td><td>10.1 (8.4)</td><td>15.3 (8.4)</td></tr>
</table>
* Immediate effect
</td>
</tr>
<tr>
<td colspan="4">Star Excursion Balance Test (SEBT) and related tests</td>
</tr>
<tr>
<td>Aminaka et al. [30]</td>
<td>Knee tape x No tape</td>
<td>Anterior direction of SEBT</td>
<td>
<table>
<tr><td>n=20</td><td>Knee Tape</td><td>No Tape</td></tr>
<tr><td>SEBT anterior (%)</td><td>63.5 (1.3)</td><td>62.8 (1.2)</td></tr>
</table>
* Immediate effect
</td>
</tr>
<tr>
<td>Chevidikunnan et al. [116]</td>
<td>Core exercise and physical therapy x Physical therapy</td>
<td>SEBT</td>
<td>
<table>
<tr><td></td><td colspan="2">Core (n=10)</td><td colspan="2">Control (n=10)</td></tr>
<tr><td></td><td>Pre</td><td>4 weeks</td><td>Pre</td><td>4 weeks</td></tr>
<tr><td>SEBT index (%)</td><td>58.1 (7.35)</td><td>78.1 (7.35)</td><td>56.3 (4.64)</td><td>68.7 (4.64)</td></tr>
</table>
</td>
</tr>
<tr>
<td>Demirci et al. [118]</td>
<td>Lower limb traction, tibial mobilization, and home exercises x Knee kinesiotaping (KT) and home exercises</td>
<td>SEBT</td>
<td>
<table>
<tr><td rowspan="2">SEBT (cm)</td><td colspan="4">Mobilization (n=18)</td><td colspan="4">KT (n=17)</td></tr>
<tr><td>Pre</td><td>Post*</td><td>2w</td><td>6w follow-up</td><td>Pre</td><td>Post*</td><td>2w</td><td>6w follow-up</td></tr>
<tr><td>Anterior</td><td>58.7 (5.8)</td><td>59.04 (5.2)</td><td>60.5 (5.1)</td><td>60.9 (5.1)</td><td>58.5 (6.5)</td><td>58.9 (6.5)</td><td>60.1 (5.9)</td><td>60.8 (5.7)</td></tr>
<tr><td>Posteromedial</td><td>81.8 (7.7)</td><td>82.2 (7.5)</td><td>84.1 (6.9)</td><td>84.5 (6.7)</td><td>82.5 (7.5)</td><td>82.9 (7.6)</td><td>83.4 (7.5)</td><td>84.3 (6.9)</td></tr>
<tr><td>Posterolateral</td><td>75.1 (8.7)</td><td>76.2 (7.7)</td><td>77.9 (7.1)</td><td>78.5 (6.8)</td><td>76.7 (10.1)</td><td>77.1 (10.3)</td><td>78.09 (9.6)</td><td>79.02 (9.2)</td></tr>
</table>
* Immediate effect
</td>
</tr>
</table>

<table>
<tr><td>Ebrahimi et al. [113]</td><td>Virtual reality training x Written education</td><td>SEBT</td><td>
<table>
<tr><td></td><td colspan="2">Virtual reality (n=12)</td><td colspan="2">Control (n=12)</td></tr>
<tr><td></td><td>Pre</td><td>8 weeks</td><td>Pre</td><td>8 weeks</td></tr>
<tr><td>Anterior (%)</td><td>0.635 (0.127)</td><td>0.709 (0.115)</td><td>0.625 (0.154)</td><td>0.701 (0.123)</td></tr>
<tr><td>Posteromedial (%)</td><td>0.694 (0.121)</td><td>0.831 (0.092)</td><td>0.737 (0.168)</td><td>0.801 (0.144)</td></tr>
<tr><td>Posterolateral (%)</td><td>0.697 (0.119)</td><td>0.815 (0.105)</td><td>0.661 (0.166)</td><td>0.761 (0.136)</td></tr>
</table>
</td></tr>
<tr><td>Fang et al. [121]</td><td>Aquatic training x Bicycling training</td><td>Y-balance test</td><td>
<table>
<tr><td></td><td colspan="2">Aquatic training (n=27)</td><td colspan="2">Bicycling training (n=27)</td></tr>
<tr><td></td><td>Pre</td><td>8 weeks</td><td>Pre</td><td>8 weeks</td></tr>
<tr><td>Anterior (%)</td><td>54.3 (15.3)</td><td>70.5 (17.1)</td><td>56.1 (17.0)</td><td>63.3 (19.4)</td></tr>
<tr><td>Posteromedial (%)</td><td>66.1 (19.6)</td><td>78.7 (21.6)</td><td>65.3 (21.0)</td><td>70.6 (23.4)</td></tr>
<tr><td>Posterolateral (%)</td><td>65.9 (25.9)</td><td>79.6 (22.7)</td><td>62.5 (26.1)</td><td>72.4 (23.8)</td></tr>
</table>
</td></tr>
<tr><td>Goel and Bhatia [112]</td><td>Patellar tape x Sham tape x No tape</td><td>SEBT</td><td>
<table>
<tr><td>n=30</td><td>Tape</td><td>Sham</td><td>No tape</td></tr>
<tr><td>SEBT anterior (cm/cm)</td><td>0.731 (0.031)</td><td>0.635 (0.027)</td><td>0.631 (0.024)</td></tr>
<tr><td>SEBT anteromedial (cm/cm)</td><td>0.701 (0.028)</td><td>0.610 (0.025)</td><td>0.607 (0.023)</td></tr>
<tr><td>SEBT anterolateral (cm/cm)</td><td>0.752 (0.025)</td><td>0.659 (0.023)</td><td>0.657 (0.022)</td></tr>
</table>
* Immediate effect
</td></tr>
<tr><td>Loudon et al. [31]</td><td>Home exercise x Physical therapy x Control</td><td>Balance and reach test</td><td>
<table>
<tr><td rowspan="2">limb-symmetric index (%)</td><td colspan="3">Reach test for 30s</td></tr>
<tr><td>Pre</td><td>8 weeks</td><td>Change (95%CI)</td></tr>
<tr><td>Control (n=11)</td><td>85.1</td><td>83.7</td><td>1.41 (-9.07 to 11.90)</td></tr>
<tr><td>Home exercise (n=9)</td><td>79.0</td><td>102.1</td><td>-23.09 (-34.67 to -11.50)</td></tr>
<tr><td>Physical therapy (n=9)</td><td>61.4</td><td>96.6</td><td>-35.25 (-46.84 to -23.67)</td></tr>
</table>
</td></tr>
<tr><td>Maryam et al. [105,106]</td><td>Kinesio taping on thigh x Kinesio taping on trunk</td><td>SEBT and reach test</td><td>
<table>
<tr><td></td><td colspan="2">Facilitatory (n=13)</td><td colspan="2">Inhibitory (n=13)</td></tr>
<tr><td></td><td>Pre</td><td>Post</td><td>Pre</td><td>Post</td></tr>
<tr><td>Anterior</td><td colspan="4" rowspan="5">not informed</td></tr>
<tr><td>Posteromedial</td></tr>
<tr><td>Posterolateral</td></tr>
<tr><td>Total</td></tr>
<tr><td>Reach test</td></tr>
</table>
</td></tr>
<tr><td>Miller et al. [117]</td><td>Hip kinesiotaping X Lumbopelvic mobilisations x Sham tape</td><td>Y-balance test</td><td>
<table>
<tr><td></td><td colspan="3">Kinesiotaping (n=?)</td><td colspan="3">Mobilization (n=?)</td><td colspan="3">Sham (n=?)</td></tr>
<tr><td></td><td>Pre</td><td>Post</td><td>3 days</td><td>Pre</td><td>Post</td><td>3 days</td><td>Pre</td><td>Post</td><td>3 days</td></tr>
<tr><td>Y-balance index (cm)</td><td colspan="9">not informed</td></tr>
</table>
</td></tr>
</table>

<table>
<tr>
<td>Motealleh et al. [62]</td>
<td>Physical therapy and Core neuromuscular training x Physical therapy</td>
<td>Y-balance test</td>
<td>
<table>
<tr><th></th><th colspan="2">Core training (n=14)</th><th colspan="2">Control (n=14)</th></tr>
<tr><th></th><th>Pre</th><th>4 weeks</th><th>Pre</th><th>4 weeks</th></tr>
<tr><td>Anterior (%)</td><td>0.62 (0.14)</td><td>0.70 (0.11)</td><td>0.63 (0.13)</td><td>0.71 (0.17)</td></tr>
<tr><td>Posteromedial (%)</td><td>0.68 (0.16)</td><td>0.85 (0.13)</td><td>0.75 (0.19)</td><td>0.78 (0.22)</td></tr>
<tr><td>Posterolateral (%)</td><td>0.66 (0.14)</td><td>0.81 (0.14)</td><td>0.65 (0.16)</td><td>0.77 (0.20)</td></tr>
</table>
</td>
</tr>
<tr>
<td>Motealleh et al. [108]</td>
<td>Lumbopelvic manipulation x Sham manipulation</td>
<td>SEBT</td>
<td>
<table>
<tr><th></th><th colspan="2">Manipulation (n=22)</th><th colspan="2">Sham (n=22)</th></tr>
<tr><th></th><th>Pre</th><th>Post</th><th>Pre</th><th>Post</th></tr>
<tr><td>Anterior (%)</td><td>67.09 (7.01)</td><td>69.22 (7.39)</td><td>66.59 (7.09)</td><td>65.81 (7.85)</td></tr>
<tr><td>Posteromedial (%)</td><td>71.04 (7.16)</td><td>71.40 (7.37)</td><td>71.81 (6.89)</td><td>71.36 (6.75)</td></tr>
<tr><td>Posterolateral (%)</td><td>70.45 (6.91)</td><td>71.09 (7.20)</td><td>70.95 (7.24)</td><td>70.77 (7.53)</td></tr>
</table>
* Immediate effect
</td>
</tr>
<tr>
<td>Ojaghi et al. [119]</td>
<td>McConnell tape x Elastic bandage</td>
<td>SEBT and single-legged stance for 20 seconds</td>
<td>
<table>
<tr><th rowspan="2">Mean only</th><th colspan="3">McConnell (n=17)</th><th colspan="3">Elastic bandage (n=17)</th></tr>
<tr><th>Pre</th><th>Post</th><th>Change</th><th>Pre</th><th>Post</th><th>Change</th></tr>
<tr><td>SEBT Anterior (cm)</td><td>99.95</td><td>103.82</td><td>3.876 (6.018)</td><td>98.74</td><td>99.82</td><td>1.088 (5.524)</td></tr>
<tr><td>SEBT Anteromedial (cm)</td><td>99.2</td><td>101.33</td><td>2.142 (5.322)</td><td>97.94</td><td>99.06</td><td>1.123 (5.630)</td></tr>
<tr><td>SEBT Medial (cm)</td><td>89.53</td><td>93.04</td><td>3.506 (7.529)</td><td>88.25</td><td>91.99</td><td>3.735 (7.072)</td></tr>
<tr><td>SEBT Posteromedial (cm)</td><td>84.35</td><td>87.41</td><td>3.059 (6.496)</td><td>81.90</td><td>86.61</td><td>4.713 (6.810)</td></tr>
<tr><td>SEBT Posterior (cm)</td><td>77.25</td><td>81.54</td><td>4.282 (5.677)</td><td>76.48</td><td>81.22</td><td>4.747 (5.826)</td></tr>
<tr><td>SEBT Posterolateral (cm)</td><td>75.91</td><td>79.05</td><td>3.142 (6.882)</td><td>75.36</td><td>78.10</td><td>2.739 (7.582)</td></tr>
<tr><td>SEBT Lateral (cm)</td><td>70.24</td><td>74.24</td><td>4.000 (7.131)</td><td>73.08</td><td>77.09</td><td>4.018 (8.169)</td></tr>
<tr><td>SEBT Anterolateral (cm)</td><td>86.62</td><td>89.92</td><td>3.302 (5.637)</td><td>86.54</td><td>87.66</td><td>1.114 (7.318)</td></tr>
<tr><td>CoP AP velocity (mm/s)</td><td>73.992</td><td>63.947</td><td>10.045 (18.458)</td><td colspan="3" rowspan="4">not informed</td></tr>
<tr><td>CoP ML velocity (mm/s)</td><td>60.004</td><td>57.584</td><td>2.420 (6.118)</td></tr>
<tr><td>CoP AP displacement (mm)</td><td>57.096</td><td>57.425</td><td>-0.328 (6.943)</td></tr>
<tr><td>CoP ML displacement (mm)</td><td>44.312</td><td>42.658</td><td>1.653 (7.469)</td></tr>
</table>
* Immediate effect
</td>
</tr>
</table>

<table>
<tr>
<td>Steinberg et al. [107]</td>
<td>Isometric exercises x Somatosensory training x Control</td>
<td>SEBT</td>
<td>
<table>
<tr><th></th><th colspan="2">Isometric (n=41)</th><th colspan="2">Somatosensory (n=28)</th><th colspan="2">Control (n=29)</th></tr>
<tr><th></th><th>Pre</th><th>12 weeks</th><th>Pre</th><th>12 weeks</th><th>Pre</th><th>12 weeks</th></tr>
<tr><td>SEBT anterior (%)</td><td>57.3 (5.7)</td><td>55.8 (5.6)</td><td>58.1 (5.5)</td><td>59.8 (5.5)</td><td>54.6 (6.3)</td><td>54.8 (5.3)</td></tr>
<tr><td>SEBT posteromedial (%)</td><td>85.3 (8.5)</td><td>85.0 (8.1)</td><td>86.6 (8.1)</td><td>91.9 (9.2)</td><td>86.0 (8.8)</td><td>87.9 (9.1)</td></tr>
<tr><td>SEBT posterolateral (%)</td><td>84.3 (9.8)</td><td>83.8 (10.0)</td><td>86.4 (8.9)</td><td>91.4 (8.9)</td><td>88.2 (9.2)</td><td>87.5 (9.2)</td></tr>
<tr><td>SEBT index (%)</td><td>89.2 (8.1)</td><td>87.8 (8.2)</td><td>92.5 (7.6)</td><td>95.4 (6.5)</td><td>91.7 (9.6)</td><td>90.7 (8.0)</td></tr>
</table>
</td>
</tr>
<tr>
<td>Sinaei et al. [120]</td>
<td>Facilitatory Kinesio taping x Inhibitory Kinesio taping</td>
<td>SEBT</td>
<td>
<table>
<tr><th></th><th colspan="2">Facilitatory (n=16)</th><th colspan="2">Inhibitory (n=16)</th></tr>
<tr><th></th><th>Pre</th><th>Post</th><th>Pre</th><th>Post</th></tr>
<tr><td>Anterior (cm)</td><td>82.49 (13.94)</td><td>89.58 (14.07)</td><td>80.57 (10.27)</td><td>83.87 (12.32)</td></tr>
<tr><td>Posteromedial (cm)</td><td>84.44 (14.51)</td><td>92.73 (18.73)</td><td>83.80 (11.31)</td><td>87.18 (11.83)</td></tr>
<tr><td>Posterolateral (cm)</td><td>80.36 (15.88)</td><td>86.93 (16.32)</td><td>77.88 (11.80)</td><td>83.75 (12.16)</td></tr>
<tr><td>Composite (cm)</td><td>82.43 (14.27)</td><td>89.75 (16.06)</td><td>80.75 (10.98)</td><td>84.93 (11.77)</td></tr>
</table>
</td>
</tr>
<tr>
<td>Song et al. [94]</td>
<td>Femoral tape x Controls</td>
<td>Anterior direction of SEBT</td>
<td>
<table>
<tr><th>n=16</th><th>Tape</th><th>Sham</th><th>No tape</th></tr>
<tr><td>SEBT anterior (%)</td><td>66.15 (4.64)</td><td>67.18 (3.74)</td><td>65.57 (4.83)</td></tr>
</table>
* Immediate effect
</td>
</tr>
<tr>
<td>Zarei et al. [110]</td>
<td>Dry needling and exercise x Exercise only</td>
<td>SEBT</td>
<td>
<table>
<tr><th></th><th colspan="3">Dry needling (n=20)</th><th colspan="3">Control (n=20)</th></tr>
<tr><th></th><th>Pre</th><th>4 weeks</th><th>6 weeks follow-up</th><th>Pre</th><th>4 weeks</th><th>6 weeks follow-up</th></tr>
<tr><td>Anterior (cm/cm)</td><td>0.76 (0.12)</td><td>0.89 (0.11)</td><td>0.90 (0.11)</td><td>0.76 (0.14)</td><td>0.81 (0.14)</td><td>0.81 (0.13)</td></tr>
<tr><td>Posterolateral (cm/cm)</td><td>0.74 (0.12)</td><td>0.83 (0.11)</td><td>0.85 (0.11)</td><td>0.74 (0.12)</td><td>0.78 (0.12)</td><td>0.77 (0.11)</td></tr>
<tr><td>Posteromedial (cm/cm)</td><td>0.67 (0.11)</td><td>0.77 (0.10)</td><td>0.78 (0.10)</td><td>0.66 (0.10)</td><td>0.69 (0.10)</td><td>0.70 (0.10)</td></tr>
</table>
</td>
</tr>
</table>

Abbreviations: KT = kinesiotaping, CoP = centre of pressure, AP = anteroposterior, ML = mediolateral, SEBT = Star Excursion Balance Test.

Supplementary material 8C. Individual data of the included studies for Q3 (mean±SD).

<table>
<tr><th>Study</th><th>Interventions</th><th>Balance Intervention</th><th>Results</th></tr>
<tr><td>Boitrago et al. [61]</td><td>Strength and Proprioceptive exercises x Education</td><td>Single-legged stance on stable and unstable surfaces</td><td>
<table>
<tr><td></td><td colspan="2">Exercise (n=30)</td><td colspan="2">Education (n=30)</td></tr>
<tr><td></td><td>Pre</td><td>6 weeks</td><td>Pre</td><td>6 weeks</td></tr>
<tr><td>NPRS (cm)</td><td>6.7 (1.7)</td><td>1.9 (1.8)</td><td>6.67 (2.3)</td><td>6.07 (2.2)</td></tr>
<tr><td>AKPS (points)</td><td>67.6 (15.2)</td><td>85.8 (12.3)</td><td>71.6 (1.11)</td><td>71.83 (11.8)</td></tr>
<tr><td>KOS-ADLS (points)</td><td>59.2 (14.2)</td><td>83 (15.5)</td><td>62.27 (19.9)</td><td>62 (18.9)</td></tr>
</table>
</td></tr>
<tr><td>Clark et al. [124]</td><td>Exercise, taping and education x Exercise and education x Taping and Education x Education only</td><td>Balance exercises using a trampet</td><td>
<table>
<tr><td></td><td colspan="3">Exercise + tape (n=20)</td><td colspan="3">Exercise (n=20)</td><td colspan="3">Tape (n=19)</td><td colspan="3">Education (n=21)</td></tr>
<tr><td></td><td>Pre</td><td>3 mo</td><td>12 mo</td><td>Pre</td><td>3 mo</td><td>12 mo</td><td>Pre</td><td>3 mo</td><td>12 mo</td><td>Pre</td><td>3 mo</td><td>12 mo</td></tr>
<tr><td>VAS (mm)</td><td>75.6 (32.6)</td><td>35.9 (28.7)</td><td>35.1 (45.1)</td><td>77.1 (44.4)</td><td>30.0 (39.9)</td><td>37.8 (43.4)</td><td>83.9 (39.8)</td><td>57.8 (38.7)</td><td>77.3 (62.8)</td><td>76.99 (41.8)</td><td>41.8 (40.6)</td><td>51.9 (53.8)</td></tr>
<tr><td>WOMAC (points)</td><td>25.2 (12.5)</td><td>11.5 (10.5)</td><td>14.8 (18.0)</td><td>23.7 (12.9)</td><td>10.0 (11.8)</td><td>15.6 (16.2)</td><td>33.4 (16.8)</td><td>20.9 (15.5)</td><td>27.6 (22.7)</td><td>28.7 (15.4)</td><td>13.8 (15.8)</td><td>22.0 (21.3)</td></tr>
</table>
* Interventions lasted 3 months
</td></tr>
<tr><td>Ebrahimi et al. [113]</td><td>Virtual reality training x Written education</td><td>Virtual games requiring single-legged stance</td><td>
<table>
<tr><td></td><td colspan="2">Virtual reality (n=12)</td><td colspan="2">Control (n=12)</td></tr>
<tr><td></td><td>Pre</td><td>8 weeks</td><td>Pre</td><td>8 weeks</td></tr>
<tr><td>VAS (mm)</td><td>5.786 (1.626)</td><td>1.714 (1.383)</td><td>5.929 (1.639)</td><td>3.071 (1.817)</td></tr>
<tr><td>AKPS (points)</td><td>68.929 (5.771)</td><td>88.357 (6.033)</td><td>69.786 (9.513)</td><td>83.000 (10.459)</td></tr>
<tr><td>Step-down test (rep)</td><td>9.691 (3.385)</td><td>15.119 (4.282)</td><td>9.857 (3.384)</td><td>13.357 (4.284)</td></tr>
</table>
</td></tr>
<tr><td>Emamvirdi et al. [125]</td><td>Exercise with valgus control instruction x Written instructions</td><td>Single-legged stance on stable and unstable surfaces</td><td>
<table>
<tr><td></td><td colspan="2">Exercise (n=32)</td><td colspan="2">Control (n=32)</td></tr>
<tr><td></td><td>Pre</td><td>6 weeks</td><td>Pre</td><td>6 weeks</td></tr>
<tr><td>VAS (cm)</td><td>6.1 (1.18)</td><td>3.1 (1.61)</td><td>6.0 (1.35)</td><td>6.1 (1.12)</td></tr>
<tr><td>Single hop test (cm)</td><td>112.43 (9.60)</td><td>140.12 (11.50)</td><td>118.60 (14.01)</td><td>117.50 (13.05)</td></tr>
<tr><td>Triple hop test (cm)</td><td>308.31 (58.44)</td><td>381.54 (33.40)</td><td>343.18 (42.66)</td><td>324.12 (35.22)</td></tr>
<tr><td>Crossover hop test (cm)</td><td>301.25 (14.63)</td><td>340.06 (18.95)</td><td>306.37 (40.47)</td><td>299.62 (36.25)</td></tr>
<tr><td>6m hop test (sec)</td><td>10.90 (0.60)</td><td>10.09 (0.62)</td><td>10.16 (0.44)</td><td>10.29 (0.67)</td></tr>
</table>
</td></tr>
<tr><td>Ferber et al. [122]</td><td>Hip and core exercises x Knee exercises</td><td>Double and single-legged stance on unstable surfaces</td><td>
<table>
<tr><td></td><td colspan="2">Hip/Core (n=111)</td><td colspan="2">Knee (n=88)</td></tr>
<tr><td></td><td>Pre</td><td>6 weeks</td><td>Pre</td><td>6 weeks</td></tr>
<tr><td>VAS (cm)</td><td>5.12 (1.66)</td><td>1.96 (1.92)</td><td>4.96 (1.66)</td><td>1.99 (2.05)</td></tr>
<tr><td>AKPS (points)</td><td>75.00 (9.74)</td><td>87.95 (11.26)</td><td>75.62 (9.81)</td><td>87.67 (10.53)</td></tr>
</table>
</td></tr>
</table>

<table>
<tr>
<td>Foroughi et al. [28]</td>
<td>**Stretching, strengthening and postural control exercises** x Stretching and strengthening exercises</td>
<td>Postural control training on unstable seat</td>
<td>
<table>
<tr><th></th><th colspan="3">Postural control (n=17)</th><th colspan="3">Control (n=16)</th></tr>
<tr><th></th><th>*Pre*</th><th>*4 weeks*</th><th>*3 months*</th><th>*Pre*</th><th>*4 weeks*</th><th>*3 months*</th></tr>
<tr><td>***NRS (cm)***</td><td>6.11 (1.21)</td><td>1.58 (0.93)</td><td>1.17 (0.95)</td><td>6.25 (1.18)</td><td>3.31 (1.13)</td><td>3.75 (0.93)</td></tr>
<tr><td>***AKPS (points)***</td><td>76.23 (4.77)</td><td>88.05 (4.75)</td><td>92.41 (4.80)</td><td>75.25 (5.10)</td><td>81.93 (5.79)</td><td>82.06 (5.99)</td></tr>
<tr><td>***Step-down test (rep)***</td><td>17.55 (2.81)</td><td>24.37 (3.35)</td><td>X</td><td>17.30 (3.36)</td><td>20.56 (4.11)</td><td>X</td></tr>
</table>
* Interventions lasted 4 weeks
</td>
</tr>
<tr>
<td>Loudon et al. [31]</td>
<td>**Home exercise** x **Physical therapy** x Control</td>
<td>Single-legged balance with open and closed eyes, and balance and reach exercise</td>
<td>
<table>
<tr><th>limb-symmetric index (%)</th><th colspan="3">***Control (n=11)***</th><th colspan="3">***Home exercise (n=9)***</th><th colspan="3">***Physical therapy (n=9)***</th></tr>
<tr><th></th><th>*Pre*</th><th>*8 weeks*</th><th>*Change (95%CI)*</th><th>*Pre*</th><th>*8 weeks*</th><th>*Change (95%CI)*</th><th>*Pre*</th><th>*8 weeks*</th><th>*Change (95%CI)*</th></tr>
<tr><td>***VAS (mm)***</td><td>37.7</td><td>34.8 (17.7)</td><td>3.55 (-3.88; 10.97)</td><td>36.1</td><td>20.4 (17.3)</td><td>15.72 (7.52; 23.93)</td><td>56.0</td><td>23.0 (16.2)</td><td>33.00 (24.79; 41.21)</td></tr>
<tr><td>***AKPS (points)***</td><td>68.8</td><td>71.2 (11.8)</td><td>-2.36 (-8.29; 3.56)</td><td>71.0</td><td>86.6 (11.2)</td><td>-15.56 (-22.11; -9.00)</td><td>60.8</td><td>84.3 (8.1)</td><td>-23.56 (-30.11; -17.00)</td></tr>
<tr><td>***Lunges (%)***</td><td>84.6</td><td>88.4</td><td>-3.68 (-17.49; 10.13)</td><td>84.0</td><td>97.4</td><td>-13.45 (-28.72; 1.82)</td><td>69.8</td><td>103.9</td><td>-34.18 (-49.44; -18.91)</td></tr>
<tr><td>***Step-down (%)***</td><td>78.6</td><td>78.0</td><td>0.68 (-12.06; 13.41)</td><td>74.8</td><td>100.5</td><td>-25.65 (-39.73; -11.57)</td><td>61.1</td><td>90.5</td><td>-28.70 (-42.78; 14.62)</td></tr>
<tr><td>***Leg press (%)***</td><td>79.0</td><td>79.8</td><td>-0.87 (-10.68; 8.95)</td><td>72.2</td><td>105.6</td><td>-33.41 (-44.27; -22.56)</td><td>59.4</td><td>100.7</td><td>-41.25 (-52.10; -30.39)</td></tr>
<tr><td>***Squat (rep)***</td><td>19.5</td><td>19.9 (3.7)</td><td>-0.36 (-2.40; 1.68)</td><td>19.0</td><td>22.8 (3.6)</td><td>-3.89 (-6.14; -1.63)</td><td>14.4</td><td>18.9 (3.7)</td><td>-4.44 (-6.70; -2.19)</td></tr>
</table>
% = limb-symmetric index
</td>
</tr>
<tr>
<td>Mahmoud and Kamel [114]</td>
<td>**Hip and balance exercise** x Hip exercise</td>
<td>Single-legged stance on stable and unstable surfaces</td>
<td>
<table>
<tr><th></th><th colspan="2">Balance (n=30)</th><th colspan="2">Control (n=30)</th></tr>
<tr><th></th><th>*Pre*</th><th>*4 weeks*</th><th>*Pre*</th><th>*4 weeks*</th></tr>
<tr><td>***VAS (cm)***</td><td>5.3 (1.7)</td><td>1.9 (0.9)</td><td>5.6 (2.0)</td><td>3.5 (1.5)</td></tr>
</table>
</td>
</tr>
<tr>
<td>Molgaard et al. [127]</td>
<td>Knee exercises x **Knee and foot exercise, plus foot orthoses**</td>
<td>Single-legged stance and double-legged stance on wobble board</td>
<td>
<table>
<tr><th></th><th colspan="3">Knee (n=15)</th><th colspan="3">Foot (n=17)</th></tr>
<tr><th></th><th>*Pre*</th><th>*4 months*</th><th>*12 months*</th><th>*Pre*</th><th>*4 months*</th><th>*12 months*</th></tr>
<tr><td>***KOOS Pain (points)***</td><td>68 (10)</td><td>69 (15)</td><td>74 (16)</td><td>64 (14)</td><td>78 (11)</td><td>79 (12)</td></tr>
<tr><td>***KOOS ADL (points)***</td><td>78 (11)</td><td>78 (15)</td><td>81 (15)</td><td>76 (10)</td><td>83 (10)</td><td>85 (12)</td></tr>
<tr><td>***KOOS Sport (points)***</td><td>46 (14)</td><td>50 (16)</td><td>50 (13)</td><td>45 (14)</td><td>54 (15)</td><td>60 (17)</td></tr>
</table>
* Interventions lasted 4 months
</td>
</tr>
</table>

<table>
<tr>
<td>Rabelo et al. [32]</td>
<td>Strength exercises x Strength and motor control exercises</td>
<td>Single-legged stance</td>
<td>
<table>
<tr><td></td><td colspan="4">Strengthening (n=17)</td><td colspan="4">Motor Control (n=17)</td></tr>
<tr><td></td><td>Pre</td><td>4 weeks</td><td>3 mo</td><td>12 mo</td><td>Pre</td><td>4 weeks</td><td>3 mo</td><td>12 mo</td></tr>
<tr><td>NPRS (cm)</td><td>6.6 (1.0)</td><td>2.2 (1.6)</td><td>3.0 (2.4)</td><td>2.2 (1.6)</td><td>6.1 (1.4)</td><td>2.0 (1.7)</td><td>1.7 (1.6)</td><td>1.3 (1.8)</td></tr>
<tr><td>AKPS (points)</td><td>67.5 (11.3)</td><td>83.7 (8.3)</td><td>83.3 (12.0)</td><td>84.8 (9.8)</td><td>67.1 (7.6)</td><td>85.8 (9.2)</td><td>91.4 (7.0)</td><td>89.0 (8.2)</td></tr>
</table>
* Interventions lasted 4 weeks
</td>
</tr>
<tr>
<td>Shadloo et al. [126]</td>
<td>Vibration training x Conventional training</td>
<td>Single-legged stance exercises in stable and unstable surfaces with open and closed eyes</td>
<td>
<table>
<tr><td></td><td colspan="3">Vibration (n=15)</td><td colspan="3">Control (n=15)</td></tr>
<tr><td></td><td>Pre</td><td>4 weeks</td><td>6 weeks</td><td>Pre</td><td>4 weeks</td><td>6 weeks</td></tr>
<tr><td>VAS (cm)</td><td>7.53 (2.13)</td><td>2.00 (1.66)</td><td>1.20 (0.94)</td><td>7.53 (1.45)</td><td>2.00 (1.54)</td><td>1.40 (1.50)</td></tr>
<tr><td>AKPS (points)</td><td>62.46 (8.25)</td><td>88.06 (3.12)</td><td>88.86 (3.56)</td><td>64.53 (7.49)</td><td>88.66 (3.01)</td><td>88.73 (3.03)</td></tr>
<tr><td>Leg press (rep)</td><td>10.8 (1.01)</td><td>17.6 (1.80)</td><td>18.8 (0.94)</td><td>11.07 (0.88)</td><td>17.26 (1.53)</td><td>19.13 (0.91)</td></tr>
</table>
* Interventions lasted 4 weeks
</td>
</tr>
<tr>
<td>Steinberg et al. [107]</td>
<td>Isometric exercises x Somatosensory training x Control</td>
<td>Single-legged stance exercises in stable and unstable surfaces with open and closed eyes</td>
<td>
<table>
<tr><td></td><td colspan="2">Isometric (n=41)</td><td colspan="2">Somatosensory (n=28)</td><td colspan="2">Control (n=29)</td></tr>
<tr><td></td><td>Pre</td><td>12 weeks</td><td>Pre</td><td>12 weeks</td><td>Pre</td><td>12 weeks</td></tr>
<tr><td>VAS (cm)</td><td>4.56 (1.79)</td><td>4.03 (2.73)</td><td>5.04 (2.13)</td><td>4.45 (2.18)</td><td>5.36 (1.98)</td><td>5.00 (2.23)</td></tr>
<tr><td>VAS PIT (cm)</td><td>4.64 (2.22)</td><td>3.36 (2.24)</td><td>4.83 (2.24)</td><td>3.17 (2.76)</td><td>4.51 (2.16)</td><td>6.15 (1.73)</td></tr>
</table>
</td>
</tr>
<tr>
<td>van Linschoten et al. [123]</td>
<td>Exercise therapy x Usual care</td>
<td>Balance exercises</td>
<td>
<table>
<tr><td></td><td colspan="3">Exercise (n=65)</td><td colspan="3">Usual care (n=66)</td></tr>
<tr><td></td><td>Pre</td><td>3 months</td><td>12 months</td><td>Pre</td><td>3 months</td><td>12 months</td></tr>
<tr><td>NRS rest (cm)</td><td>4.14 (2.3)</td><td>2.30 (2.5)</td><td>1.43 (2.2)</td><td>4.03 (2.3)</td><td>3.22 (2.8)</td><td>2.61 (2.9)</td></tr>
<tr><td>NRS activity (cm)</td><td>6.32 (2.2)</td><td>3.81 (2.9)</td><td>2.57 (2.9)</td><td>5.97 (2.3)</td><td>4.60 (3.0)</td><td>3.54 (3.38)</td></tr>
<tr><td>AKPS (points)</td><td>64.4 (13.9)</td><td>78.8 (15.5)</td><td>83.2 (14.8)</td><td>65.9 (15.2)</td><td>74.9 (17.6)</td><td>79.8 (17.5)</td></tr>
</table>
* Interventions lasted 3 months
</td>
</tr>
<tr>
<td>Yalfani et al. [128]</td>
<td>Sensorimotor exercise x No intervention</td>
<td>Single-legged stance exercises in stable and unstable surfaces with open and closed eyes</td>
<td>
<table>
<tr><td></td><td colspan="2">Sensoriomotor (n=16)</td><td colspan="2">No intervention (n=16)</td></tr>
<tr><td></td><td>Pre</td><td>12 weeks</td><td>Pre</td><td>12 weeks</td></tr>
<tr><td>Pain (cm)</td><td>6.00 (1.13)</td><td>2.66 (1.17)</td><td>7.00 (1.85)</td><td>6.53 (1.35)</td></tr>
</table>
</td>
</tr>
</table>

Abbreviations: NRS = numeric pain scale, AKPS = Anterior Knee Pain Scale, VAS = visual analogue scale, WOMAC = Western Ontario and McMaster Universities Osteoarthritis Index, KOOS = Knee injury and Osteoarthritis Outcome Score, ADL = Activities of Daily Living, KOS-ADLS = Knee Outcome Survey - Activities of Daily Living Scale.

*Interventions in bold indicate the group that applied balance exercises
